# In situ learning-based spin engineering of pulsed dynamic nuclear polarization

**DOI:** 10.1126/sciadv.aeh4467

**Published:** 2026-09-16

**Authors:** Filip V. Jensen, José P. Carvalho, Nino Wili, Asbjørn Holk Thomsen, David L. Goodwin, Lukas Trottner, Claudia Strauch, Anders Bodholt Nielsen, Niels Chr. Nielsen

**Affiliations:** ^1^ Interdisciplinary Nanoscience Center (iNANO) and Department of Chemistry, Aarhus University, Gustav Wieds Vej 14, DK-8000 Aarhus C, Denmark.; ^2^ Department of Mathematics, Aarhus University, Ny Munkegade 118, DK-8000 Aarhus C, Denmark.; ^3^ Institute for Stochastics and Applications, University of Stuttgart, Wankelstrasse 5, 70563 Stuttgart, Germany.; ^4^ Institut für Mathematik, Ruprecht-Karls-Universität Heidelberg, Mathematikon, Im Neuenheimer Feld 205, 69120 Heidelberg, Germany.

## Abstract

Pulsed dynamic nuclear polarization (DNP) is currently receiving substantial interest as a means to enhance the sensitivity of nuclear magnetic resonance (NMR) and magnetic resonance imaging by orders of magnitude. It has also received much attention as a central ingredient in many modalities of electron–spin–involved quantum sensing. Relative to spin engineering associated with NMR, the design of efficient pulsed DNP experiments with a broad experimental scope is challenged by large electron-nuclear spin systems, large electron–spin–involved interactions, and instrumental nonidealities and limitations. All of this may challenge traditional NMR-like theoretical and numerical pulse sequence engineering. Exploiting state-of-the-art instrumentation and taking advantage of the high sensitivity of DNP relative to NMR, we here demonstrate the use of combinations of Bayesian machine learning methods and constrained random walk procedures to design pulse sequences in situ, by experiments, directly on the spin systems responding to spectrometer instructions. For trityl and nitroxide samples, it is demonstrated that efficient broadband DNP pulse sequences can be designed in situ with experimental protocols benchmarked against in silico analogs.

## INTRODUCTION

Coherent spectroscopy has undergone a tremendous evolution in an increasing span of disciplines, since the pioneering magnetic resonance experiments in the 1940s (*1*–*8*). This has been driven by an intricate interplay between the development of increasingly advanced instrumentation and the exploitation of powerful analytical and numerical methods to design pulsed experiments while taking advantage of the gradually improving instrumentation. This process has been further fueled by emerging needs for information on the structures and dynamics of molecular systems in many disciplines. A prime example is nuclear magnetic resonance (NMR), which may be considered the “easy” case due to weak spin interactions and the associated long coherence lifetimes available for manipulation of nuclear spin systems. In the context of magnetic resonance, advanced pulse engineering is, to an increasing extent, being applied to methods involving electron spins, such as paramagnetic NMR, electron paramagnetic resonance (EPR), and dynamic nuclear polarization (DNP). DNP bridges NMR and EPR by transferring polarization from highly polarized electron spins to nuclear spins to boost the sensitivity by orders of magnitude and diversifying spectral information. The challenges encountered in EPR and DNP are the large electron–spin interactions, fast relaxation, and the mere size of the electron-nuclear spin systems, which may limit current experiment design principles and instrumental realization of optimal experiments.

Several design strategies may be pursued, including analytical [e.g., effective Hamiltonian (*9*–*15*)], numerical [e.g., optimal control (*16*–*21*)], and experimental [e.g., feedback control (*22*–*24*)] methods. All of these have advantages and disadvantages that depend heavily on the complexity of the spin systems to be addressed, including the number of spins, interaction strengths, coherence lifetimes, and instrumental capabilities. Realization of pulsed DNP, e.g., in the context of quantum sensing, definitely provides an example of a system much more complicated than typical NMR systems. An unpaired electron spin may interact much more strongly with the surroundings than nuclear spins, implying that typical electron-nuclear spin systems may consist of hundreds to thousands of spins, the coherence lifetimes are typically very short, and the instrumentation may be challenged by insufficient microwave (MW) irradiation power, large MW field inhomogeneity and pulse transients, and insufficient time resolution. In such cases, it is relevant to further explore and potentially reconsider the experiment design process. Both analytical effective Hamiltonian–based and numerical nonlinear optimization or optimal control methods are typically limited in the size and complexity of the spin systems that can be handled. In the latter case, this is simply due to insufficient capacity to store and operate with density matrices with dimensions scaling exponentially with the number of spins. The most obvious drawback of operating with too small spin systems is that they may be inaccurate or may not fully represent the spin systems manipulated and observed by the spectrometer. In this view, it may be interesting to reverse the situation, i.e., letting the spectrometer form the basis for an experiment-driven pulse sequence design procedure by reporting on the spin system for a representative molecular object, as recently explored in combination with optimal control in the context of EPR (*22*) and DNP (*24*). Accordingly, a feedback-loop setup may be used to develop experimental methods that respond optimally to the actual type of spin system and experimental conditions. Realizing in situ experiment design obviously calls for high experimental sensitivity and fast repeatability. This may be feasible for pulsed DNP. The challenge remaining is that experimental observables are typically much less detailed than the information content used in numerical gradient–based (*16*, *18*) or Krotov-type (*17*, *19*) optimal control procedures.

Taking DNP as a guinea pig to explore large spin–system in situ pulse sequence engineering, it is relevant to have (i) access to front-end instrumentation (ii), state-of-the-art pulse sequences, and (iii) molecular systems that are suitable for testing and are representative for targeted molecular systems. On the basis of pulsed EPR and DNP instrumentation following and extending the protocol of Doll and Jeschke (*25*), we have recently developed broadband DNP pulse sequences such as BEAM (*26*), PLATO (*27*), and constrained random walk (cRW)–OPT (*28*) which through the combined use of exact effective Hamiltonian theory (EEHT) (*11*, *12*), single-spin-vector effective Hamiltonian theory (SSV-EHT) (*13*–*15*), cRW (*28*), and nonlinear optimization and optimal control (*21*, *29*) have enabled realization of increasingly strong performance DNP in terms of the range of electron-spin offsets (width of the EPR line) covered. We note that chirped-pulse electron nuclear double-resonance (ENDOR) (*30*) and, in the context of coherent spectroscopy, DNP echoes (*31*) have also been developed, demonstrating the capability of the setup. The validity of such investigations as a test bench for experimental-driven optimization is reinforced by a remarkably good correspondence between theoretical-numerical and experimental data for well-characterized trityl-glycerol-water samples.

In this framework, we here investigate the use of a modern machine learning–based Bayesian probabilistic optimization approach to develop pulse sequences directly on the spectrometer to address large electron-nuclear spin systems and experimental conditions in the design process of DNP experiments. This method is chosen to exploit the signal intensity as the only directly available feedback information differing from the most typical full–density operator information used in numerical gradient– or Lagrangian-based optimal control optimization methods (*16*–*18*, *20*, *21*). By having a huge nuclear spin system involved as the receiver of the transfer process with direct incorporation of learning elements, our approach also sets itself apart from earlier EPR (*22*) and DNP chopped random basis optimal control (*24*) feedback-loop approaches.

The Bayesian method works on black-box functions, meaning that it can be applied as long as the objective (e.g., a measurable polarization transfer efficiency) can be evaluated. In addition to this, it is designed for objective functions (which, in our case, depend on amplitudes and phases of control pulses) that are expensive to evaluate. These attributes make it highly enticing for implementation in the context of pulsed experiments. Since Bayesian optimization does not have any gradients to guide the optimization, it can be beneficial to instead constrain the search space of the control variables. This allows a larger number of variables, in this case MW pulses, to be efficiently optimized. In our case, this constraint is a resonance condition derived from a cRW effective Hamiltonian model (*28*). The performance of the Bayesian optimization was tested for both numerical and experimental evaluations in the context of electron-offset compensating pulsed DNP for trityl and TEMPO nitroxide samples.

## RESULTS

### Bayesian in situ pulse sequence design

**Fig. 1.**
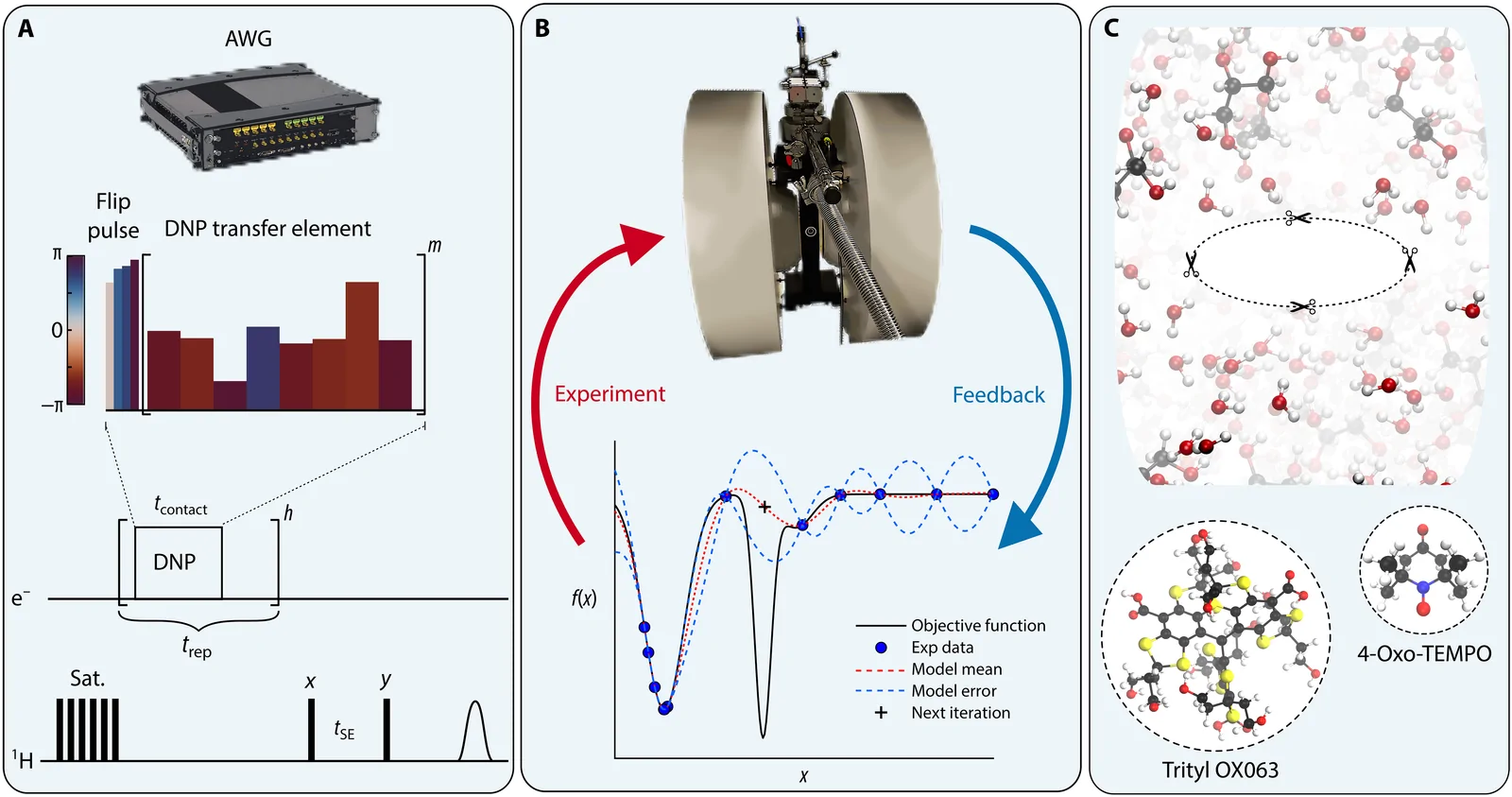
Setup for in situ pulse sequence design. Schematic representation of the experimental setup used for in situ Bayesian development of DNP pulse sequences. (**A**) The generation of amplitude- and phase-modulated pulses for an initial flip (or excitation) pulse and a periodic DNP transfer element which combined mediate electron to proton polarization transfer. The pulse sequences are generated using an AWG. The overall pulse program contains the initial flip pulse and the DNP transfer element at the MW channel for polarization transfer and an RF channel containing initial saturation pulses and a solid echo for proton signal detection. (**B**) Part of the spectrometer magnet and the ENDOR/EPR probe for MW/RF pulsing and detection of signals from an inserted frozen radical-containing sample. The spectrometer is controlled by a Bayesian optimization algorithm in a feedback-loop setup receiving response from the experiment and outputing the control variables for the next experiment. (**C**) The sample is a frozen solution containing in our example trityl OX063 or 4-Oxo-TEMPO radicals.

In situ experiment design involves a close interplay between the controlling optimization software, the instrument and its operating system, and the sample providing the response signal to evaluate. This forms the vehicle for iterative pulse sequence development in an artificial intelligence feedback-loop setup. The core principle of our proposed setup is illustrated in Fig. 1, involving instrumentation, pulse sequence, learning-based feedback control, and a sample with a representative spin system. First, it is crucial that the DNP instrument, in a stand-alone setup, is capable of realizing advanced DNP pulse sequences to drive polarization efficiently from an unpaired electron spin to surrounding protons. A key ingredient in the process is a fast AWG to transmit shaped pulse sequences to the sample as illustrated in Fig. 1A, with the overall pulse sequence sketched in the lower part of the panel. Here, it should be noted that what we optimize is the DNP element (DNP box in Fig. 1A) with the experiment additionally involving repeated pumping of polarization to, and via spin diffusion through, the large nuclear spin system. The performance of the system is clearly important for our exploration of in situ optimization versus state-of-the-art pulsed DNP experiments, designed analytically and/or numerically. One may argue that, in later more general practical applications, the in situ design protocol may be even more useful in exploiting instrumental setups, e.g., with lower power and time digitization. The DNP experiment is invoked on a sample in the spectrometer (i.e., in an EPR/ENDOR resonator located in the magnet), providing feedback to the Bayesian optimization, as illustrated in Fig. 1B. The samples, and thereby their “real-life” representative spin systems, used in this study are illustrated in Fig. 1C. This involves trityl OX063 or 4-Oxo-TEMPO radicals in a deuterated-glycerol-H_2_O-D_2_O solutions frozen at 80 K. We note that the feedback-loop setup in Fig. 1 is used also with pulse sequences generated by Monte Carlo (random) generation of pulse sequences, for reference, and with a constrained variant of Bayesian optimization using cRW procedures (*28*) to improve convergence in case of well-defined resonances (vide infra).

The X-band DNP spectrometer (9.7 GHz for electrons, 14.8 MHz for ^1^H) is controlled by MATLAB (*32*), which readily enables integration of Bayesian optimization protocols also available in MATLAB. The Bayesian optimization operates directly on the experimental feedback DNP signal. For the narrow-EPR-line trityl sample, it uses multiple measurements taken at different electron–spin offsets, whereas for the broad-EPR-line nitroxide sample, it uses a single measurement taken at the carrier frequency. The method operates through learning, without guidance from gradients or other constraints. This may require a relatively large number of evaluations to reach optimal performance depending on the number of variables in the optimization. In practical applications, as demonstrated below, it appears advisable to use a relatively low number of variables. In the interest of fast optimization, we, in addition to this Bayesian stand-alone approach, explored combinations with cRW (*28*). This method provides a fast way to generate sequences fulfilling a DNP resonance condition (vide infra) to limit the search space of the Bayesian optimization. The latter approach may be useful in cases where dominant small-spin-system interactions are expected to be present, releasing the focus of the Bayesian optimization from the large-spin-system network of interactions. Details on the flow of operations in the Bayesian optimization are given in Materials and Methods. A description of the Bayesian implementation in MATLAB for pulse sequence optimization, along with an explanation for constraining the pulse optimization, is provided in the Supplementary Text and figs. S1 and S2.

When optimizing multivariate functions, in our case with a complicated impact on the DNP transfer performance, it is relevant to address the difference between Bayesian optimization and other commonly used nonlinear optimization methods such as simplex (Nelder-Mead) methods (*33*), gradient methods (*34*), simulated annealing (*35*), and evolutionary algorithms (*36*). In the present context, the observable is the intensity accumulated in large spin systems over a broad offset range and with contributions coming from direct and many indirect (spin diffusion type) polarization transfer pathways. Although the objective may be the same, the learning process is different. The key feature of Bayesian optimization is a global surrogate model based on all available observations and with explicit experimental variance. Conventional simplex or gradient following methods are local in operation and typically do not explicitly handle variance. In addition to this, gradient methods require gradient estimation, such as finite difference, to work as a zero-order optimization (i.e., when only function values are accessible). Simulated annealing and evolutionary algorithms are global methods, but instead of using a surrogate model, they work by perturbing an element or population from the search space. The distinction of Bayesian optimization relative to such methods may be considered using a simple definition of learning as the ability to accurately predict the next experiment evaluation. This definition is useful because Bayesian optimization, as a global optimizer, will attempt to survey the search space, resulting in many observations with suboptimal results. This could be considered a deficiency of the method, but if the model predicts these observations as being suboptimal, it should instead be considered part of the learning process. With this distinction, one may argue that simplex methods, simulated annealing, and evolutionary algorithms do not incorporate this mode of learning as they use no predictive model. We should note that some gradient methods, such as trust region methods (*37*, *38*), do use a predictive model, but it is only local compared with the global model in Bayesian optimization. The global and predictive nature of the Bayesian method may offer a substantial advantage in the optimization of pulse sequences, particularly those addressing unknown spin systems, instrument-related effects across multiple transfer steps, and noise in the feedback involved in the optimization. This applies in particular when the set of observables for the iterative evaluation is small. We should note, however, that the rapid development of machine learning algorithms may alter this picture and may lead to more efficient learning-based methods than explored in this initial study with well-established Bayesian protocols.

### Optimization of the DNP transfer pulse sequence for trityl

**Fig. 2.**
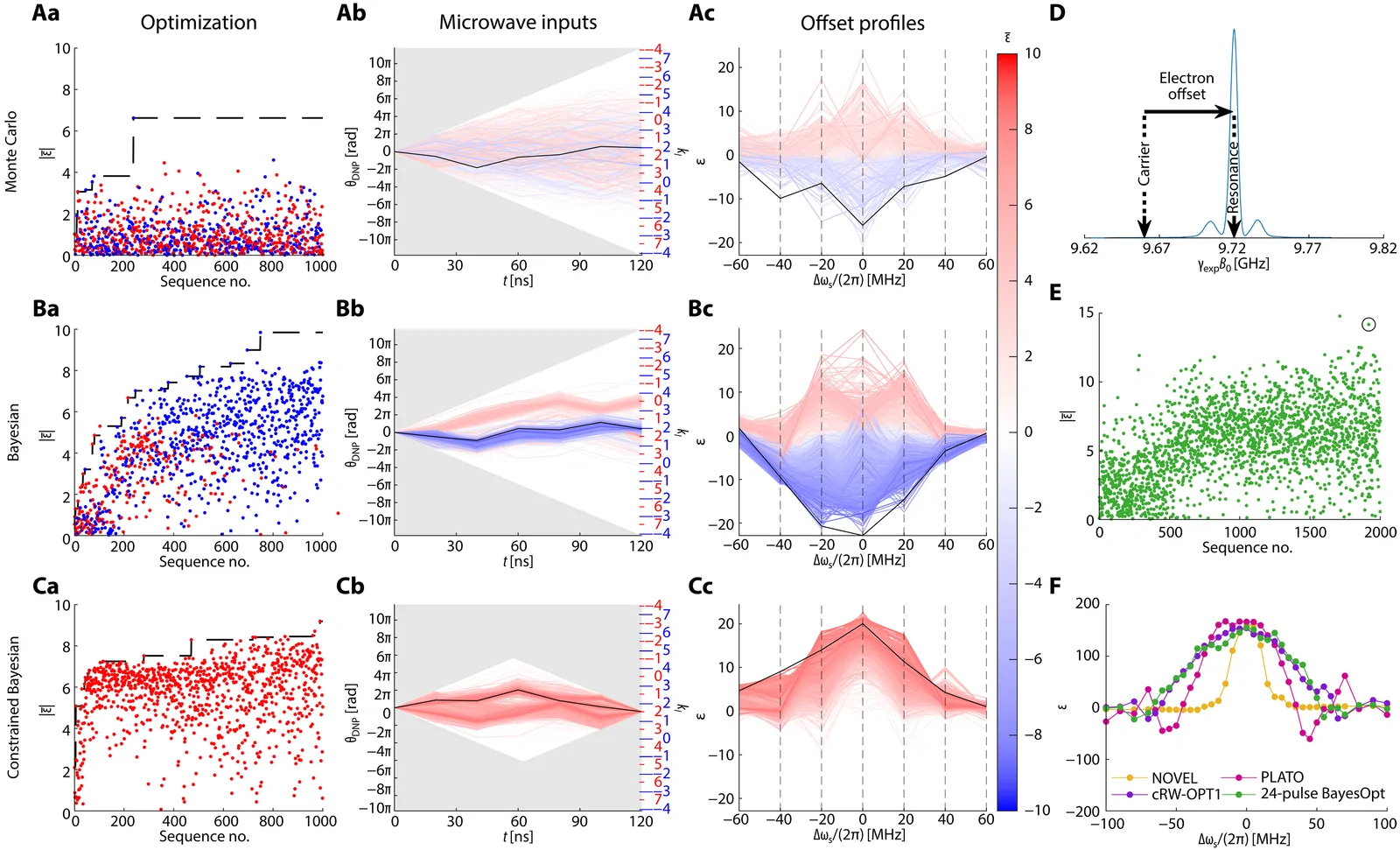
Performance of Bayesian optimization of DNP transfer element using trityl. In situ optimization of the DNP transfer element in Fig. 1A.  (**A** to **C**) Results for three optimization methods: (A) Monte Carlo, (B) Bayesian, and (C) constrained Bayesian (1000 evaluations each, 51 hours of optimization). (**Aa** to **Ca**) Absolute average enhancement $|\bar{\epsilon }|$ for the offset profile; red/blue dots represent match to DQ/ZQ resonances. (**Ab** to **Cb**) Accumulated nutation angle during each of the evaluated sequences. Colored numbers reflect *k*_*I*_values for DQ (red) and ZQ (blue) resonances according to Eq. 1. (**Ac** to **Cc**) Electron-spin offset profile. In columns 2 and 3, the color is weighted by the absolute average enhancement. The best sequences are marked in black. (**D**) Different electron-spin offsets were realized by placing the MW carrier at different frequencies with respect to the EPR resonance of trityl. (**E**) Constrained Bayesian optimization (2000 evaluations, 115 hours) for a 24-pulse DNP transfer element (black circle marks the sequence with best offset profile when repeated with higher resolution in electron-spin offset). (**F**) Offset profiles for the 24-pulse BayesOpt sequence marked with a black circle in (E) compared with NOVEL, PLATO, and cRW-OPT1. The sequences to be optimized used 6 [(A) to (C)] and 24 [(E) and (F)] pulses (modulation time tm = 120 ns; 49-MHz maximal MW field strength; initial flip pulse of amplitude 47 MHz and duration 5 ns; see Fig. 1A). The measurements were performed at 80 K using trityl OX063 in glycerol-d5 [(A) to (E)] and glycerol-d8 (F). The DNP transfer element was repeated 1 to 30 [(A) to (C)] and 6 [(E) and (F)] times. The buildup time was 2 [(A) to (C) and (E)] and 64 (F) s. Offset profiles for trityl in glycerol-d_5_ can be found in fig. S3. Settings for Bayesian optimizations are provided in tables S1 and S2.

As a first example, demonstrating the relative performance of in situ Bayesian optimization of pulsed DNP experiments, we optimize the DNP transfer element (using a prior fixed strong 90° excitation pulse) for a sample of 5 mM trityl OX063 in a H_2_O:D_2_O:glycerol-d_5_ solution (1:3:6 by volume) at 80 K. Trityl radicals are well characterized in terms of electron-nuclear spin interactions, and they have been used extensively in dissolution-DNP (*39*) and as probe for evaluating the performance of pulsed DNP experiments (*40*). The latter applies to experiments spanning from the simple NOVEL (*41*, *42*) sequence to the more recent and advanced PLATO (*27*) and cRW-OPT (*28*) methods. This facilitates direct comparison to state-of-the-art DNP pulse sequences, which, for this well-known system, may be designed analytically and numerically with a remarkably good match to experimental data (*26*–*29*, *42*–*46*). An important aspect is that trityl, through the relatively narrow EPR resonance (see Fig. 2D), enables direct optimization and evaluation of pulse sequences in terms of electron–spin resonance offset.

To demonstrate in situ DNP pulse sequence development, Fig. 2 illustrates different aspects of optimizations with different protocols using the trityl radical system described above. Our aim is broadband DNP, which for the narrow-resonance (see Fig. 2D) trityl sample is invoked by adding the response from experiments executed with electron-spin resonance at different offsets; specifically, 0, ±20, ±40, and ±60 MHz. For the optimization, we explore a DNP transfer element with 6 *x*-phase pulses each of 20-ns duration with up to 30 repetitions of this element and limiting the MW nutation frequency to a maximum of 49 MHz. The three explored optimization protocols are Monte Carlo (i.e., random MW amplitudes), Bayesian optimization, and constrained Bayesian as illustrated in panels a to c, respectively. In the latter case, we use cRW to limit evaluations to pulse sequences fulfilling a DNP resonance. Using this setup, the columns in Fig. 2 illustrate, for each method, the outcome of an optimization with 1000 evaluations (i.e., different MW pulse sequences). The left column (panels 1) displays the absolute value of the DNP enhancement (ϵ; enhancement relative to direct excitation of the ^1^H spins) averaged over the specified electron-spin offsets for the 1000 pulse sequences with the dashed line accumulating the highest transfer efficiency. The second column (panels 2) shows the accumulated nutation angle during the *t*_m_ = 120-ns pulse sequence element with one trajectory for each of the 1000 pulse sequence elements, where the electron-spin offset is set to zero. The accumulated nutation angle is defined as $\theta^{\text{DNP}}=\sum_{i=1}^{6}\omega_{i}^{\text{MW}}\Delta t$ with $\omega_{i}^{\text{MW}}$ being the amplitude of the *x*-phase pulse *i*, the amplitude can be positive and negative, and Δt = 20 ns the length of each pulse. The ticks and numbers in red and blue to the right of the trajectories mark the angles corresponding to a zero-quantum (ZQ, blue) and double-quantum (DQ, red) resonance using the definition (in angular units)

$$\theta^{\text{DNP}}=\pm \left(\omega_{0I}t_{m}-k_{I}2\pi \right)$$ (1)

Here, the + and − signs correspond to DQ and ZQ, respectively, $\omega_{0I}$ denotes the Larmor frequency of the protons spins, $\omega_{m}=2\pi /t_{m}$ is the modulation frequency of the MW pulse sequence element, and $k_{I}$ is an integer (the colored numbers correspond to different $k_{I}$ values) (*28*). A more detailed description of these parameters and the resonance condition is given in Materials and Methods. The third column (panels 3) shows the detected enhancements ϵ for the 1000 pulse sequences as a function of electron-spin resonance offset with the dashed lines indicating the offsets used in the optimization. We note that sequences matching or approaching a DQ resonance have positive intensity (marked in red in correspondence with the angle trajectories in proximity of a DQ resonance) and, likewise, sequences matching or approaching a ZQ resonance are associated with negative intensity (marked in blue).

Addressing specifically the Monte Carlo (i.e., random pulse amplitudes within the constraints of the maximum pulse amplitude) optimization in Fig. 2A, it appears that, among 1000 sequences, a relatively equal mixture of pulse sequences in proximity of ZQ or DQ resonances over a very broad span of $k_{I}$ values is found. Many of those, however, do not match perfectly, resulting in destructive ZQ/DQ overlap or low transfer due to MW inhomogeneity (vide infra). This is evidenced by the enhancement factors in Fig. 2Aa, with the maximum approaching 7, but the average/SD being 1.0/0.87, and is also seen from the offset profiles in Fig. 2Ac.

Substantially better results are, as demonstrated in Fig. 2B, obtained by optimization of 1000 pulse sequences using machine learning Bayesian optimization of feedback-loop intensities from DNP experiments. It is clear that the sequences rapidly improve through the Bayesian optimization, reaching a maximum enhancement factor of 10 and average/SD values of 4.1/2.1 for all sequences and 5.4/1.8 for the last 200 evaluations. It is seen here that the sequences induced by optimization concentrate around the two $k_{I}$ values, 0 (DQ) and 2 (ZQ) (see Fig. 2Bb), and provide more intense/broadband profiles (see Fig. 2Bc). It is evident that Bayesian optimization results from a learning process with many of the sequences being trials and thereby likely associated with low enhancement (see Fig. 2Ba), while it searches for the best sequences. The best sequences are represented by the points with highest integrated enhancement factors. In this particular case, a relatively high fraction of the best pulse sequences is also of ZQ nature.

It is observed that the Bayesian optimizations find the best sequences for particular values of $k_{I}$. This finding can be related to our previously established knowledge that DNP pulse sequences with finite, but not too large effective fields work best as they are not excessively influenced by MW inhomogeneity (*27*, *28*). This motivates the combination of Bayesian optimization with constraints toward low values of $\theta^{\mathrm{DNP}}$. This may be achieved by constraining the search space of the Bayesian optimization by cRW procedures as described earlier (*28*) in combination with EEHT (*11*) and nonlinear optimization. An example of the performance of such constrained Bayesian optimization, with all pulse sequences constrained to fulfill the DQ resonance condition $k_{I}$ = 2, is illustrated in Fig. 2C. The enhancement factor plot (Fig. 2Ca) shows exclusively DQ behavior and a much faster convergence to high transfer efficiencies. The effect of constraining the optimization to the $k_{I}$ = 2 resonance condition is clearly visible in Fig. 2Cb with the result of broadband sequences with high enhancement as seen in Fig. 2Cc. We should note that, upon close inspection, the Bayesian and constrained Bayesian optimizations in this particular case provide the best sequence in the unconstrained Bayesian optimization with the accumulated angle $\theta^{\text{DNP}}$ of 1.2 rad (71°) being 0.2 rad (10°) below the $k_{I}$ = 2 ZQ resonance at 1.4 rad (81°). This deviation may be ascribed to effects from electron-spin offset and MW inhomogeneity. It is emphasized that this particular sequence has a low effective field, indirectly reinforcing our statement that large effective fields render the pulse sequences more susceptible to MW inhomogeneity, which in this practical setup is substantial (*27*, *28*).

To further document the important finding that in situ optimization finds sequences least susceptible to MW inhomogeneity effects, fig. S4 maps the enhancement factor as a function of $\theta^{\text{DNP}}$ for all optimizations in Fig. 2. It is clearly evident that the best sequences are associated with the DQ $k_{I}$ = 2 resonance characterized by a linear field of $\theta^{\text{DNP}}/t_{m}$ = 1.87 MHz. This happens with less probability for the Monte Carlo optimization but occurs clearly for the unconstrained Bayesian optimization. The latter provides two bands with the largest enhancement seen for the ZQ $k_{I}$ = 2 resonance and around half enhancement for the DQ $k_{I}$ = 0 resonance, with a linear field of $\theta^{\text{DNP}}/t_{m}$ = 14.8 MHz being identical to the less broadband NOVEL experiment. We should also direct attention to fig. S5, illustrating the distribution of sequences in Fig. 2 and their enhancements as a function of the number of repetitions of the DNP transfer element. All of them provide good sequences except the one to four repetitions with the overall transfer element being too short for the present pseudosecular hyperfine coupling to ensure good polarization transfer.

For further testing of the Bayesian optimization approach, the experimental optimizations presented in Fig. 2 (Aa to Cc) were also investigated with an in silico approach. Here, the evaluation of a given pulse sequence was not based on the experimental transfer performance but rather on the performance of a simulated polarization transfer for a two-spin e–${}^{1}$H system. Additional information on numerical simulations is presented in Materials and Methods. The numerical analysis is presented in fig. S6 with corresponding optimization settings in table S3. By comparison of the in silico and in situ approaches, it is seen that the features are similar but with the trend that the experimental results provide more broadband performances. This can, to a certain extent, be ascribed to the linewidth of the EPR line (Fig. 2D) which for the numerical calculations is assumed infinitely narrow.

With these comparative performance evaluations at hand, we proceeded with constrained Bayesian optimization of pulse sequences with more pulses. In silico and in situ constrained Bayesian optimization using 6, 12, and 24 pulses for the transfer element are presented in figs. S7 and S8 with corresponding optimization settings in tables S4 and S5, respectively. From these data, it is evident that by increasing the number of pulses, the performance of the transfer elements in terms of bandwidth does get better. However, the cost of increasing the number of variables/pulses for the Bayesian optimization is that it takes more evaluations to find good hits. We direct attention to optimization of 24-pulse DNP elements as illustrated in Fig. 2E, leading to an enhancement factor of 15 (averaged over the selected offset points). The broadband excitation profile of this 24-pulse DNP pulse sequence (pulse sequence, henceforth denoted BayesOpt, is given in table S6) was then compared with NOVEL, PLATO, and cRW-OPT1 pulse sequences in Fig. 2F. The sequences provide enhancement factors approaching 180 and the average enhancements, when integrated over the offset range from −60 to 60 MHz, of 98 (cRW-OPT1), 35 (NOVEL), 69 (PLATO), and 96 (BayesOpt), respectively, for a trityl OX063 glycerol-d_8_ sample (64-s buildup). We note that by using the Bayesian in situ experiment design protocol, we found pulse sequences with similar broadband excitation profile as the previously state-of-the-art analytical-numerical designed cRW-OPT1 sequence (*28*). This demonstrates that, on this well-characterized trityl OX063 system, in situ Bayesian optimization enables design of pulse sequences with comparable bandwidth to those designed with the best methods developed so far. This validates the usefulness of in situ Bayesian experimental optimization, providing a strong case for optimization on systems without similar knowledge of the involved spin system. This may lead to superior pulse sequences and, importantly, increase spin engineering insight into the function of DNP experiments in complex spin systems and/or nonideal instrumental circumstances.

### Optimization of the excitation pulse of the DNP experiment for trityl

**Fig. 3.**
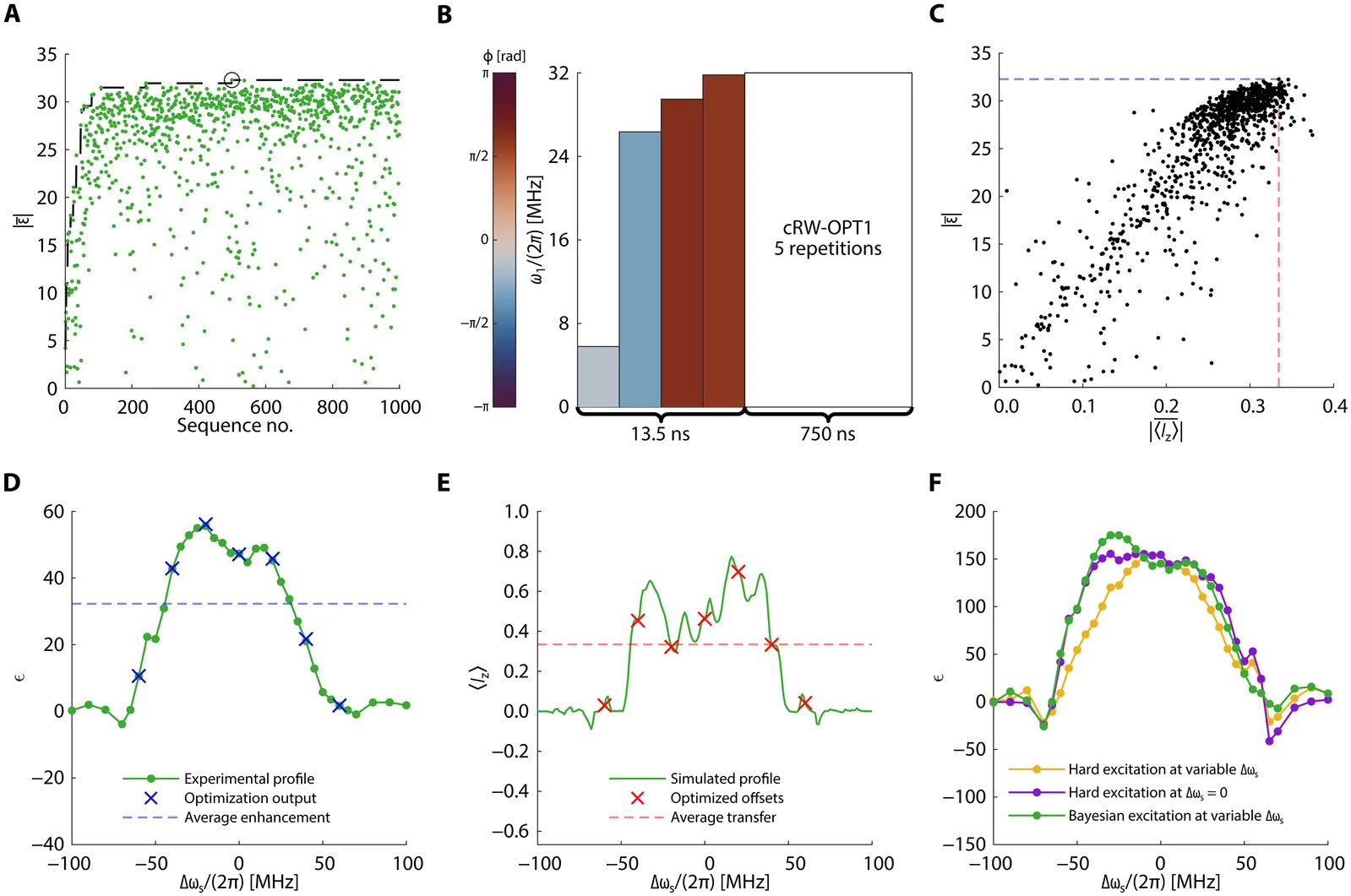
In situ and in silico comparison of flip pulse optimization for DNP using trityl. (**A**) Bayesian in situ optimization of a four-pulse excitation sequence with flexible total duration of 5 to 20 ns (all pulses equal length, amplitude up to 45 MHz, free phase) followed by cRW-OPT1 DNP transfer (five repetitions; 5-s buildup) using trityl OX063 in glycerol-d_5_. The optimization involved 1000 evaluations with a total runtime of 98 hours. (**B**) The best four-pulse excitation sequence [marked by circle in (A); table S7] followed by cRW-OPT1 DNP transfer. (**C**) Comparison of simulated (horizontal) and experimental (vertical) enhancements for all sequences in (A). (**D**) Offset profile for the best excitation sequence. Blue crosses show the enhancements found during optimization, green dots show repetition of the experiment for a finer grid of offsets, and the dashed line shows the average. (**E**) Simulated profile of the best excitation sequence. Crosses show simulated transfer at the optimized offsets, and the dashed line shows the average. Note that the dashed lines in (C) correspond to the best sequence as shown in (D) and (E). (**F**) Offset profiles for cRW-OPT1 using the best excitation sequence and a hard excitation pulse (amplitude 32 MHz). The carrier of the hard excitation pulse was either the same as for the DNP transfer sequence or fixed at the trityl resonance. These experiments used trityl OX063 in glycerol-d_8_ and a buildup time of 64 s. Settings for the Bayesian optimizations are given in table S8.

The most common (*29*, *44*, *45*, *47*) DNP transfer elements mediate transverse spin-locked electron-spin $S_{x}$ to proton-spin $I_{z}$ polarization transfer. This implies that a typically short and intense excitation pulse is required to generate transverse *x*-phase electron-spin coherence before polarization transfer via spin locking. Recently, in the development of highly broadband DNP experiments, such as the cRW-OPT pulses sequences (*28*), we demonstrated that the initial pulse may impede the broadband capability of the overall experiment. Hence, for transverse spin-lock DNP, it is relevant to address optimization of the initial excitation pulse as well as the DNP transfer element. To demonstrate diversity in the use of in situ experimental Bayesian pulse sequence optimization, Fig. 3 illustrates optimization of the phases and amplitudes of an initial four-pulse excitation sequence. The optimization allowed flexibility of the total duration to be between 5 and 20 ns to optimally match DNP transfer with a previously developed 30-pulse (150 ns total, 5-ns pulses) cRW-OPT1 broadband DNP transfer element (*28*). This implies that the optimization considers both excitation and transfer while only optimizing the former element. The optimized target pulse element is not necessarily a perfect broadband π/2 pulse but a pulse that works optimally with the specific cRW-OPT1 element in generating the best transfer from electron to nuclear spin polarization. In this case, the Bayesian optimization was unconstrained.

Figure 3A reports on 1000 evaluations of an in situ Bayesian optimization of a four-pulse excitation pulse, with green dots representing the transfer efficiency of each sequence and the dashed black line representing the fast establishment of a large DNP enhancement factor for the combined excitation pulse and cRW-OPT1 pulse sequence. The pulse amplitudes could reach a maximum of 45 MHz, the phase of each pulse was allowed to vary freely, and the total length of the excitation pulse sequence could vary between 5 and 20 ns. The optimal excitation pulse sequence, represented by the black circle, is illustrated in Fig. 3B concatenated with the cRW-OPT1 pulse sequence (repeated 5 times). In Fig. 3C, we investigate the correlation between average enhancement factors obtained experimentally (vertical) and numerically (horizontal) for the 1000 excitation pulse sequences evaluated in Fig. 3A. A perfect match between simulations and experiments would yield a straight-line correlation and would indicate that the two-spin system indeed does represent part of the spin system. Deviation from a linear correlation, as seen, illustrates that the in situ optimization captures either spin-system or instrumental aspects that are not included in the theoretical/numerical model used for the simulations.

A similar analysis was also carried out for the sequences from Fig. 2 (A to C and E) with results provided in fig. S9. In this case, however, direct comparison may be complicated by the observation that experimental polarization transfer is often faster than in small-spin system simulations. For the analysis in Fig. 3C, this aspect is handled by using a hard–flip pulse experiment to optimize the experimental contact time (5 repetitions, 750 ns) and the simulated contact time (11 repetitions, 1650 ns). However, since the transfer element changes between experiments in Fig. 2, it is difficult to establish the contact time best matching each experiment. This aspect is illustrated in fig. S9 correlating simulations using different scalings of the contact time with experimental data. In all cases, it is difficult to find a convincing correlation. One notable observation is that the 24-pulse BayesOpt sequence identified in Fig. 2E and analyzed in Fig. 2F does not perform so well in any of the simulations (marked with green circle in fig. S9). This could be ascribed to insufficient modeling of MW inhomogeneity or to too sparse sampling of electronspin offsets in the simulations. To investigate this in more detail, the electron offset and MW inhomogeneity profile of the sequence was subject to a more detailed simulation as provided in fig. S10. These simulations reveal that the simulations are not capable of reaching the experimentally observed performance even within a wide range of MW nutation frequencies. This indicates that the simulation does not sufficiently describe the details of the experiment. This finding is highly interesting as in situ pulse sequence development may provide important insight into the quality of models used for numerical simulations and in silico experiment design. A more detailed analysis of these aspects will be a subject to further analysis.

Figure 3D illustrates the experimental broadband profile of the combined optimized excitation pulse sequence and cRW-OPT1 DNP transfer pulse sequence. The pulse sequence is evaluated at the offset points used in the optimization marked by blue crosses and repetition of the experiments with a finer grid of offset values represented by the green dots and line. This demonstrates that optimization with relatively few points covers well the full span of electron-spin offsets. This may be naturally ascribed to the 10- to 15-MHz linewidth of the trityl radical, the effect of which may be rationalized by folding of the offset profile with the shape of the trityl EPR line (see Fig. 2D). The offset profiles in Fig. 3E represent numerical simulations of the optimal pulse sequence calculated for the offsets used in the optimization as well as for a finer set of offset points. The two-spin simulations indeed validate the broadband behavior identified experimentally; however, with more pronounced intensity variations, which may be ascribed to insufficient modeling of the true spin dynamics by an electron-nuclear spin-pair system, as already hinted at by Fig. 3C. Figure 3F compares cRW-OPT1 measurements conducted using either the Bayesian optimized excitation pulse or single-pulse (amplitude, 45 MHz; length, 5 ns) excitation. The single-pulse excitation was executed in two ways: With the carrier frequency of the excitation pulse on-resonance (i.e., $\Delta \omega_{S}/\left(2\pi \right)$ = 0) or, experimentally more relevant, with the same carrier frequency for the excitation pulse and the transfer element (i.e., $\Delta \omega_{S}/\left(2\pi \right)$) varied throughout the offset plot. This last approach was also used for the Bayesian four-pulse sequence. It is evident that the Bayesian excitation pulse effectively prevents losses at the outer parts of the offset profile as achieved by the single-pulse matching the “idealized” case, where the excitation pulse remains always on-resonance (i.e., $\Delta \omega_{S}/\left(2\pi \right)$ = 0). This is obviously important for implementation for systems with broad EPR lines.

### Optimization on TEMPO

**Fig. 4.**
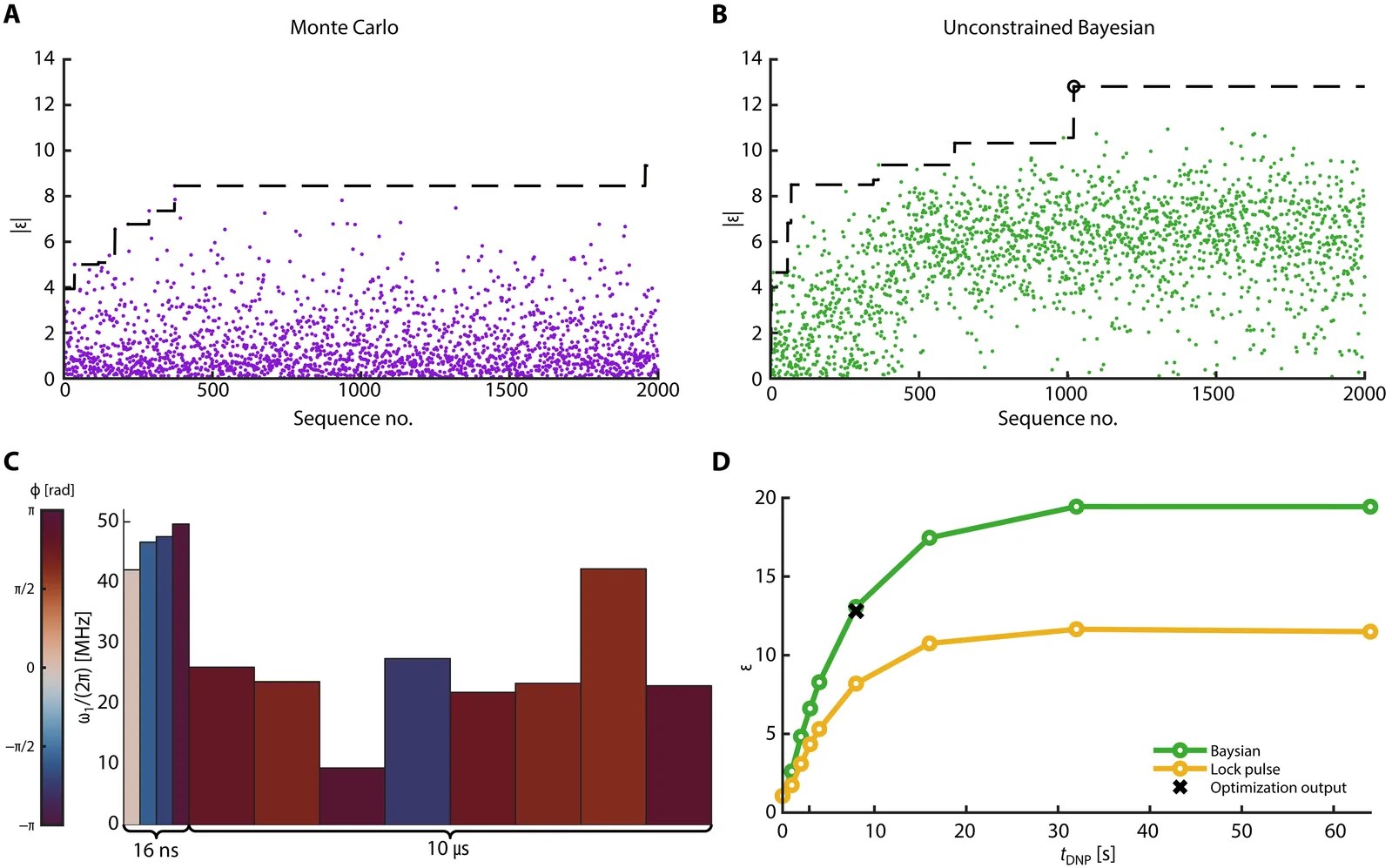
Bayesian optimization of DNP using TEMPO. In situ experimental Monte Carlo and Bayesian optimization of excitation pulse and polarization transfer elements of pulsed DNP experiments for a 2 mM sample of 4-Oxo-TEMPO. (**A**) Evaluated enhancements for Monte Carlo optimization of control variables (2000 evaluations, 42 hours). (**B**) Evaluated enhancements for an unconstrained Bayesian optimization of control variables (2000 evaluations, 49 hours). (**C**) The best pulse sequence is marked by the circle in (B). The duration of the excitation pulses was 4 ns each, and transfer pulses were 1250 ns each (the time axis is not to scale). The pulse sequence is given in table S11. (**D**) Buildup curve for the best sequence found using Bayesian optimization compared with the buildup for a hard excitation pulse of 52 MHz for 5.5 ns followed by a spin-lock pulse of constant amplitude of 31.2 MHz for 10 μs. The cross shows the enhancement measured during the optimization with a buildup time of 8 s. Settings for the Bayesian optimizations are given in table S12.

While the narrow EPR line of trityl and its well-studied spin dynamics (*26*–*29*, *42*–*46*) make this sample ideal for investigating broadband DNP transfer, it simultaneously makes it a less interesting use-case for DNP transfer in more typical EPR cases that are influenced by larger hyperfine couplings, electron-spin coupling to different nuclear spin species, and broad EPR resonances. Therefore, we explored the use of learning-based in situ experimental design of DNP pulse sequences for a more challenging system, where current analytical-numerical design strategies may not yet suffice due to difficulties in establishing good models for complicated electron-nuclear spin dynamics. Accordingly, Fig. 4 provides an exploratory Bayesian optimization of DNP for the 2 mM nitroxide-radical 4-Oxo-TEMPO in a dimethyl sulfoxide (DMSO)-d_6_:D_2_O:H_2_O solution (6:3:1 by volume) at 80 K. In this case, the unpaired electron is strongly coupled to ^14^N (spin *I* = 1) and further to numerous ^1^H spins implying that it is not straightforward to assume the same resonance conditions as used in the constrained Bayesian approach demonstrated above.

Initial in situ tests were performed to determine the overall structure of the pulse sequence to be optimized, e.g., trial setups to determine whether initial flip pulses were desirable or the number of pulses to be used for efficient Bayesian optimizations. These exploratory data are presented in fig. S11 with corresponding Bayesian settings in tables S9 and S10. On the basis of these results, DNP pulse sequences involving both the excitation pulse and the subsequent DNP transfer element were optimized using unconstrained Bayesian optimization and compared with Monte Carlo (random) optimization. The excitation pulse involved four pulses with a maximum amplitude of 49 MHz, free phase, and a total length of the excitation pulse sequence of 5 to 20 ns, while the DNP pulse sequence element comprised 8 equally long pulses spanning 10 μs with free phase variation and maximum amplitude of 52 MHz. We note that this transfer element is not repeated, and thereby, the sequence representing the testing object in this case is very different from the nanosecond-scale pulse sequences used above for the case of trityl. We also note that this aspect, and with this which method will be best in general terms, will be subject to further exploration. It is, however, evident from Fig. 4 that Bayesian optimization (Fig. 4B) provides better sequences (enhancement factor of 13) than those resulting from a similar 2000-sequence Monte Carlo optimization (Fig. 4B, enhancement factor of 9).

The best Bayesian optimized pulse sequence (corresponding to the circle in Fig. 4B) is shown in Fig. 4C. Comparison of this sequence with a spin-lock pulse sequence with MW amplitude 31 MHz is illustrated in Fig. 4D revealing a gain in sensitivity by 70% when using the Bayesian-optimized DNP pulse sequence. Three points should be noted here: (i) the spin-lock sequence used a larger amplitude than NOVEL. However, as seen in fig. S12, quite similar efficiency is observed at the true NOVEL condition, (ii) the broadband DNP pulse sequences did not work better than the spin-lock sequence in this case, and (iii) for the purpose of illustration, we deliberately chose a low concentration of TEMPO to focus on solid-effect DNP rather than the higher concentration typically generating DNP by the cross effect. These aspects will independently be subject to further investigation.

## DISCUSSION

We have in this work explored the use of learning-based optimization methods to develop magnetic resonance pulse sequences in situ on a spectrometer. Our target has been DNP using Bayesian-based learning methods in a feedback-loop control system using a home-built X-band pulsed DNP/EPR spectrometer with fast AWG’s and strong MW amplification. This setup was mimicked by a corresponding numerical optimization protocol based on density operator calculations.

The first target was trityl OX063 in a “DNP juice” solution, which represents a widely used system for method development and applications of DNP. Various pulsed DNP experiments have recently been developed and characterized for trityl, which here represents a “model system” to explore Bayesian-based learning procedures for in situ method development. For this system, we could identify: (i) Monte Carlo–based in situ pulse sequence optimization is capable of producing good pulse sequences, but (ii) the association with Bayesian learning procedures provides better sequences faster, and (iii) both faster convergence and overall better performing sequences can be obtained by limiting the search space for the Bayesian learning by cRW procedures. The latter operate by constraining the prediction model–based search to sequences fulfilling ZQ or DQ resonances. Remarkably good pulse sequences were found, with similar performance to the best sequences developed for this well-characterized system by analytical and numerical methods. The Bayesian approach clearly found sequences that were broadband in terms of electron–spin offset and efficiently compensated for experimental MW inhomogeneity. Furthermore, it also identified that the pulse sequence performance can be improved beyond the limits imposed by a simple two-spin system. This is supported by deviations from a linear correlation between experimental data and numerical calculations for the experimentally developed pulse sequences. Overall, the experiments on the trityl system may be considered a proof of concept for in situ learning-based pulse sequence optimization.

Taking a more challenging system where solid-effect-type pulse sequences may be less efficient, as here represented by the low-concentration (2 mM) sample of the 4-Oxo-TEMPO nitroxide, we demonstrated improvements of the DNP enhancement by 70% relative to the NOVEL experiment. The overall transfer efficiencies are not impressive, but these initial experiments serve as a compelling case for the use of in situ learning-based methods to further develop pulsed DNP experiments and for potential inspiration to analytical-numerical design protocols. The proposed in situ pulse sequence design may find applications in wider ranges of magnetic resonance, including liquid- and solid-state NMR, EPR, and the DNP approach demonstrated here may be of considerable interest for electron–spin–based quantum sensing and information technology.

## MATERIALS AND METHODS

### Experimental setup

In situ experimental optimization and evaluation of pulsed DNP experiments were carried out on a home-built X-band pulsed EPR/DNP spectrometer [based on the design of Doll *et al.* (*25*)] equipped with a Keysight M8190A 14-bit/8GSa and Zürich Instruments HDWAG4 16-bit/2.4 GSa (Zürich Instruments, Zürich, CH) arbitrary waveform generators on electron and nuclear spins, respectively, a 2-kW Applied Systems Engineering 176 X TWT MW amplifier, a SpinCore GX-5 iSpin NMR console (SpinCore Technologies Inc., Gainesville, FL), a 300-W NanoNord RF amplifier (NanoNord A/S, Aalborg, Denmark), and a Bruker MD4 electron-nuclear double resonance probe (Bruker BioSpin, Rheinstetten, DE) extended with an external tuning and matching circuit.

**Fig. 5.**
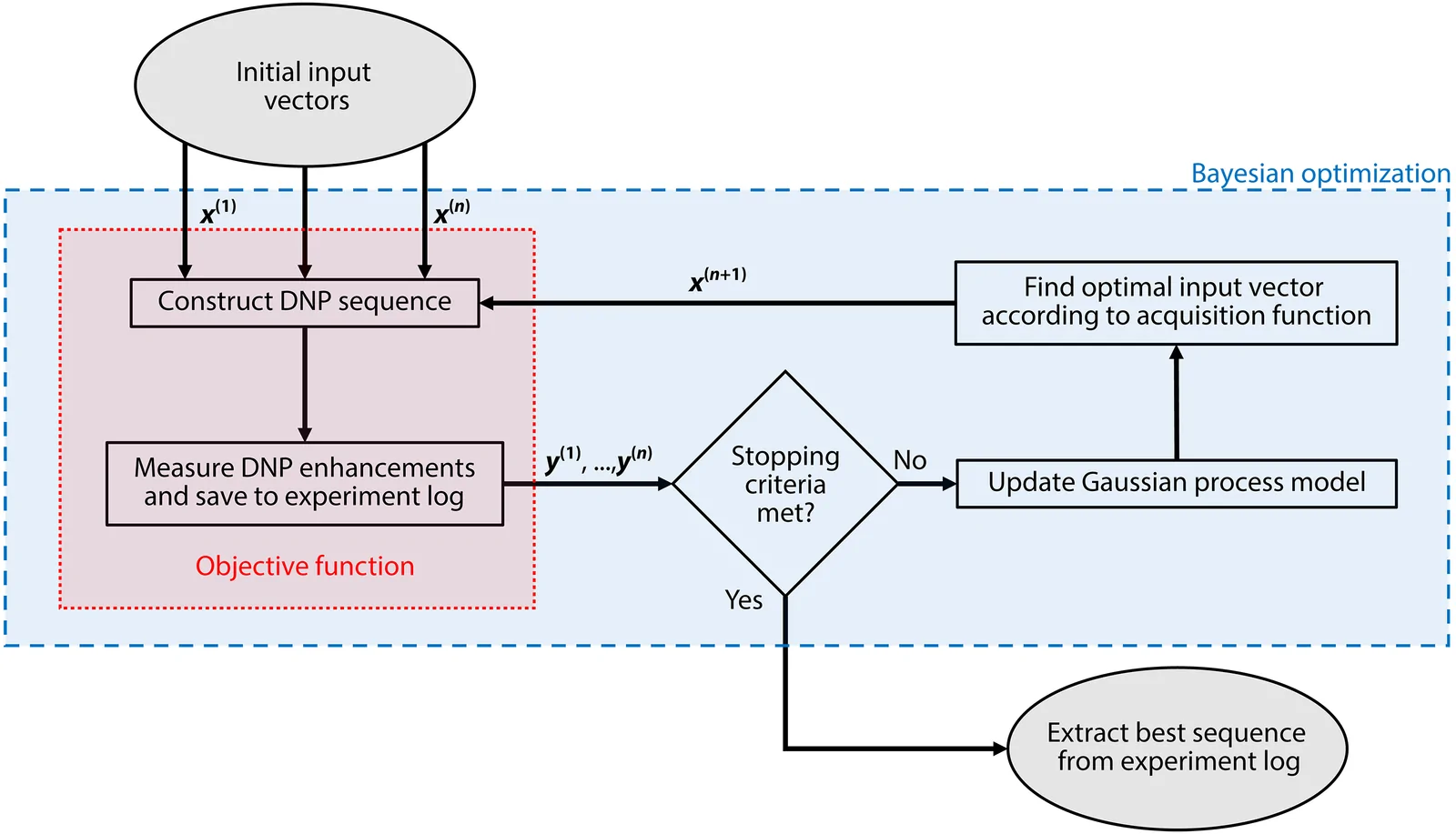
Flow diagram for in situ Bayesian optimization. Diagram showing the procedure used for in situ Bayesian learning-based optimization. First, a set of random input vectors is given to the objective function. Each of these vectors is then used to construct a pulsed DNP sequence, which is executed on the experimental setup. Each sequence and the resulting DNP enhancements are stored in an experiment log for future reference. After the initial input vectors have been evaluated, a Gaussian process model is initialized from the experiment log, and the chosen type of acquisition function is optimized on the basis of this model. The (approximate) optimizer of the acquisition function is then used as the input vector for the next evaluation of the objective function, completing the feedback loop. When certain user-defined conditions, such as number of iterations, are met, the loop is set to stop, and, subsequently, the best sequence can be extracted from the experiment log.

All experiments used the pulse sequence in Fig. 1A, basically involving a MW channel for polarization transfer and an RF channel for proton detection. The pulse sequence initially saturates ^1^H with a set of *S* = 11 pulses of duration 1.32 μs separated by $\tau_{\text{sat}}$ = 1 ms. The ^1^H signal was detected using a solid-echo sequence $\pi /2-t_{\mathrm{SE}}-\pi /2$ with $t_{\text{SE}}$ = 25 μs; RF pulses typically used an RF field strength of 227.3 kHz, corresponding to a π/2 pulse time of 1.10 μs. Each experiment used a repetition time of $t_{\text{rep}}$ = 2 ms. The pulse sequence and signal detection were executed as part of the optimization flow diagram shown in Fig. 5. Experimental polarization enhancements (denoted ϵ) are defined by the ratio between the DNP-enhanced signal intensity and the thermal equilibrium signal intensity. It is noted that we for easier comparison of offset plots with positive transfer efficiencies defined the sign of the enhancement factor for the DQ sequences in Fig. 2F so that at $\Delta \omega_{S}$ = 0 it is positive (i.e., inverted the sign). To be consistent, this sign inversion were used in all figures. For each experimental session, a series of calibrations was done to determine the MW resonator profile (maximum available MW field strength), with a representative example given in fig. S13.

An EPR field sweep of the three samples is presented in fig. S14. Experimental evaluations of $T_{1}$ relaxation properties for electron and proton spins of trityl OX063 in glycerol-d_5_ and glycerol-d_8_ and 4-Oxo-TEMPO in DMSO-d_6_ samples are presented in fig. S15.

### Samples

Experiments were conducted at 80 K on samples of 5 mM trityl OX063 in a H_2_O:D_2_O:glycerol-d_5_ solution (1:3:6 by volume; note the higher concentration of protons in this system relative to the commonly used DNP juice, which, due to its higher concentration of ^1^H, has lower overall polarization transfer), 5 mM trityl OX063 in a H_2_O:D_2_O:glycerol-d_8_ solution (1:3:6 by volume; with a lower concentration of ^1^H), and 2 mM 4-Oxo-TEMPO in DMSO-d_6_:D_2_O:H_2_O solution (6:3:1 by volume).

### Bayesian optimization

Pulsed DNP sequences were optimized in situ on the spectrometer with a feedback-loop control shown in Fig. 5 using Bayesian optimization, which was originally developed as a black-box optimization method in machine learning. Bayesian optimization is inspired by Bayesian statistical methods to determine a probabilistic surrogate model of the objective function (*48*, *49*). The objective feedback DNP enhancements *f(***x***)* are obtained from a pulse sequence, which is in turn determined from an input vector **x** of variables as described in the Supplementary Text and figs. S1 and S2. Given the underlying stochastic dynamics of the spin system and instrumentation, we model the observed feedback DNP enhancement for a given input vector **x** stochastically as y=f[x]+ε for independent Gaussian noise, $\varepsilon ∼N\left\{0,\sigma^{2}\right\}$, where the SD σ>0 summarizes the measurement error of the experimental setup. The Bayesian approach to this regression model now postulates to encode our uncertainty about the feedback model *f* by treating it as a stochastic object. To this end, we first put a rather uninformative prior distribution on *f* and then sequentially update our beliefs on its nature from obtained experimental evidence $y^{\left(i\right)}=f\left[x^{\left(i\right)}\right]+\varepsilon^{\left(i\right)}$, i=1,…,n, for implemented input vectors $x^{\left(1\right)},\ldots ,x^{\left(n\right)}$. Specifically, we put a Gaussian process prior (*50*) on the objective function *f* such that before collecting experimental evidence, the feedback is modeled to be drawn from a multivariate normal distribution

$$f\left[x^{\left(1\right)}\right],\ldots ,f\left[x^{\left(n\right)}\right]∼N\left\{M,\Sigma \right\}$$ (2)

where **M** is a mean vector with elements $M_{i}=\mu_{0}\left[x^{\left(i\right)}\right]$ and Σ is a covariance matrix with elements $\Sigma_{\mathrm{ij}}=\Sigma_{0}\left[x^{\left(i\right)},x^{\left(j\right)}\right]$. The functions $\mu_{0}$ and $\Sigma_{0}$ are called the mean function and kernel, respectively, and are chosen before the optimization. For the observed feedback DNP enhancements $y^{\left(i\right)}=f\left[x^{\left(i\right)}\right]+\varepsilon^{\left(i\right)}$ on the spectrometer, we therefore obtain the Gaussian prior

$$y^{\left(1\right)},\ldots ,y^{\left(n\right)}∼N\left\{M,\Sigma +\sigma^{2}𝕀\right\}$$ (3)

Our implementation of Bayesian optimization used a built-in MATLAB function *bayesopt* that uses a constant mean function and the ARD Matérn 5/2 kernel (*51*).

The optimization is initialized by a number of Monte Carlo evaluations; in our case, 4. The feedback is used to determine the hyperparameters used for the prior in Eq. 2. We can then predict the feedback $f\left[x^{\left(n+1\right)}\right]$ at an arbitrary input vector $x^{\left(n+1\right)}$ by calculating its posterior distribution given evaluations $y^{\left(1\right)},\ldots ,y^{\left(n\right)}$ and corresponding input vectors $x^{\left(1\right)},\ldots ,x^{\left(n\right)}$ using Bayes’ formula. This conditional distribution is explicitly given by

$$\begin{array}{c} f\left[x^{\left(n+1\right)}\right]|y^{\left(1\right)},\ldots ,y^{\left(n\right)},x^{\left(1\right)},\ldots ,x^{\left(n\right)}\\ ∼N\left\{\mu_{n}\left[x^{\left(n+1\right)}\right],\sigma_{n}^{2}\left[x^{\left(n+1\right)}\right]\right\} \end{array}$$ (4)

where the expectation and variance functions $\mu_{n}$ and $\sigma_{n}^{2}$ are computable from $\mu_{0}$, $\Sigma_{0}$, σ, the input vectors $x^{\left(1\right)},\ldots ,x^{\left(n\right)}$, and the corresponding noisy DNP enhancements $y^{\left(1\right)},\ldots ,y^{\left(n\right)}$ (*50*). To determine the next input vector to evaluate, an acquisition function $\text{Ac}\left[x^{\left(n+1\right)}\right]$ is used as a way to specify which attributes are desired for the posterior in Eq. 4. Specifically, it is designed to balance an inherent exploration-exploitation trade-off, thus chosen to be increasing in the posterior expectation $\mu_{n}\left[x\right]$ to exploit the learned information, *f*, in the first *n* steps while also including a penalty term involving the SD, $\sigma_{n}\left[x\right]$, to promote further exploration in the search space. An (approximate) optimizer $x^{\left(n+1\right)}$ of the acquisition function is then determined and translated to give the pulse sequence of the next experiment. This procedure is applied recursively to select the next experiment on the basis of all previous experiments and feedback until a chosen stopping criterion is met. In our case the criteria was a maximal number of evaluations, and after this the best DNP enhancement and its corresponding pulse sequence could be extracted from the experiment log. An in silico constrained Bayesian optimization using four different acquisition functions is shown in fig. S16 with settings given in tables S13 to S16. On the basis of this analysis, it was decided to use the expected-improvement-plus acquisition function further on in this work.

The time cost of the Bayesian optimization can be described both empirically and theoretically. In the present study, the fraction of the duration that was spent on experiment evaluation was 99% for Figs. 2 (B and C) and 3A, 94% for Fig. 2E, and 86% for Fig. 4B. This reveals that most of the time was spent on experiment evaluation but also that the remaining overhead of the Bayesian optimization can vary substantially. For a more efficient implementation of the Bayesian optimization, it would be important to reduce the experiment time by lowering the number of scans. This may, however, decrease the signal to noise ratio and thereby degrade the quality of the input to the Bayesian optimizer (*52*). It would be interesting to investigate in more detail the trade-off between time cost and performance of the Bayesian optimization, which will be subject to independent investigations along with exploration of other machine learning protocols. On the Bayesian algorithm itself, a majority of the overhead is attributed to the Gaussian process modeling that scales as *O*(*N*^3^), where *N* is the number of previous evaluations, and optimization of the acquisition function, which scales rapidly with the dimension of the search space (*50*, *53*). In addition to this, the number of evaluations needed for the Bayesian optimization to find an appropriate optimum also scales exponentially with the dimension of the search space (*54*).

To examine the performance of Bayesian optimization, we apply our definition of learning as the ability to accurately predict the feedback $f\left[x^{\left(n+1\right)}\right]$. For each sequence evaluation (except the first) a Gaussian process was fit to all previous evaluations and used to predict the feedback as described (vide infra). This was then compared with the experimental feedback to determine the quality of the prediction. The results of such analysis for the optimizations in Figs. 3 and 4 are seen in figs. S17 and S18, respectively. Figure S17C demonstrates a clear improvement in the predictions after 100 evaluations. From fig. S17B, it can also be seen that Bayesian optimization deliberately evaluates sequences with low predicted mean because they have a high SD. Learning is, however, not as evident in fig. S18C, which only displays slight improvement in the predictions. A highly relevant distinction between the two optimizations is the dimension of the search space. The optimization in fig. S17A (same as in Fig. 3A) used four pulses of arbitrary amplitude and phase and a variable common duration, resulting in a nine-dimensional search space. For comparison, the optimization in fig. S18A (same as in Fig. 4B) used 12 pulses of arbitrary amplitude and phase with the first 4 pulses having a variable common duration, resulting in a 25-dimensional search space. This difference in dimension may very well be the reason for the less visible ability to accurately predict the feedback in fig. S18.

### Resonances and cRW

As a gradient-free learning algorithm, Bayesian optimization will typically be implemented without any constraints to freely modulate the DNP contact pulse sequence. In the interest of optimization speed, it may, however, be useful to guide or constrain the optimizations if the system to be optimized can be associated with variables that rationally can be used to constrain the search and learning process. In the case of the DNP transfer, as illustrated by the trajectories in Fig. 2Bb, the best solutions may approach ZQ or DQ resonances, as defined in Eq. 1. While this is to be expected only in cases with dominant two-spin interactions, as may be the case for the trityl OX063 system using relatively short DNP transfer times, it may be relevant to explore the potential of adding constraints to Bayesian optimization, to improve speed of convergence, while knowing that this spin–system–wise may constrain the search space. We apply, as an example, the cRW procedure described recently in combination with EEHT-driven nonlinear optimization of DNP pulse sequences (*28*). This involves constraining the search to MW pulse sequences whose accumulated nutation angle $\theta^{\text{DNP}}$ fulfills Eq. 1. This condition relates to the spin dynamics of the DNP experiment as described in the following.

In the simple case of a hyperfine-coupled electron (S)–nuclear (I) spin-pair system the Hamiltonian may, in the electron-spin rotating frame, be expressed as

$$H=\omega_{0I}I_{z}+\Delta \omega_{S}S_{z}+S_{z}\left({\mathrm{AI}}_{z}+{\mathrm{BI}}_{x}\right)+H_{\text{MW}}\left(t\right)$$ (5)

where $\omega_{0I}$ is the nuclear Larmor frequency, $\Delta \omega_{S}=\omega_{S}-\omega_{\text{MW}}^{\text{carrier}}$ is the offset of the electron spin relative to the MW carrier at frequency $\omega_{\text{MW}}^{\text{carrier}}$, *A* is the secular hyperfine coupling, *B* is the pseudosecular hyperfine coupling, and $H_{\text{MW}}\left(t\right)$ is the Hamiltonian of the MW irradiation in the rotating frame.

In the cyclic interaction frame of both the electron and nuclear spins, it is possible, by SSV-EEHT (*15*), for a pulse sequence element with modulation time $t_{m}$ to recast the Hamiltonian as (*26*, *27*, *31*, *55*)

$$\begin{array}{c} \tilde{H}=\sum_{\kappa =x,y,z}\sum_{k_{S}=-\infty }^{\infty }a_{\mathrm{\kappa z}}^{\left(k_{S}\right)}e^{{\mathrm{ik}}_{S}\omega_{m}t}{\tilde{S}}_{\kappa }\left[AI_{z}+\frac{B}{2}\left(e^{ik_{I}\omega_{m}t}I^{+}+e^{-ik_{I}\omega_{m}t}I^{-}\right)\right]\\ -\omega_{\text{eff}}^{\left(S\right)}{\tilde{S}}_{z}+\omega_{\text{eff}}^{\left(I\right)}I_{z} \end{array}$$ (6)

where $\omega_{m}=2\pi /t_{m}$ is the angular modulation frequency of the pulse sequence, $k_{I}$ is an integer, ${\tilde{S}}_{\kappa }$ is the effective field–frame electron-spin operators, $I_{z}$, $I^{+}$, and $I^{-}$ are nuclear-spin operators, and $a_{\mathrm{\kappa z}}^{\left(k_{S}\right)}$ are Fourier coefficients induced by the applied frame transformations. The last two terms describe the effective field for the electron and nuclear spins.

From Eq. 6, it is evident that matching of values $k_{S}=\pm k_{I}$ may lead to time-independent Hamiltonian components. Taking the first-order average Hamiltonian, it becomes evident that resonances occur when the nuclear-spin effective field $\omega_{e\text{ff}}^{\left(I\right)}=\omega_{0I}-k_{I}\omega_{m}$ matches the electron-spin effective field, $\omega_{\text{eff}}^{\left(S\right)}$, which lead to the resonance condition

$$\omega_{0I}-k_{I}\omega_{m}=\mp \omega_{\text{eff}}^{\left(S\right)}$$ (7)

with $k_{I}=\text{round}\left(\omega_{0I}/\omega_{m}\right)$. The negative and positive signs correspond to ZQ and DQ resonances, respectively, leading to ${\tilde{S}}_{z}\to I_{z}$ (ZQ) or ${\tilde{S}}_{z}\to -I_{z}$ (DQ) polarization transfer.

The condition in Eq. 7 implies that ZQ or DQ resonances, and thereby DNP transfer, are obtained whenever the accumulated MW nutation angle $\theta^{\text{DNP}}$ matches the nutation angle of the nuclear spin $\omega_{0I}t_{m}-k_{I}2\pi$ as outlined in Eq. 1. This feature can be used to constrain the space of solutions for the Bayesian optimization by simply requiring that the pulse sequences in the optimization fulfill this condition, as described in more detail in the Supplementary Text. We note that the first-order effective Hamiltonian through the Fourier coefficients also provides information about the scaling factors for the ZQ and DQ Hamiltonians and thereby the time needed for efficient polarization transfer as described elsewhere (*26*, *27*, *31*, *55*).

### Numerical simulations

Numerical simulations were carried out in MATLAB (*32*) using a simple two-spin model to numerically support and describe Bayesian optimization for the trityl sample. The simulations involved density matrix evaluations under the influence of the Hamiltonian in Eq. 5. The offset $\Delta \omega_{S}$ was a variable parameter within the range of offsets chosen in the experiments. The proton Larmor frequency, $\omega_{0I}/\left(2\pi \right)$ corresponding to operation at X-band ≈ 14.8 MHz, was set by the experiment calibration. The secular and pseudosecular hyperfine coupling constants *A* and *B*, respectively, were calculated in a point-dipole model with an electron-proton distance of *r* = 4.5 Å and a polar angle of β = 45°. No powder averaging was used, as only a proportional polarization transfer is needed, as elaborated on in (*55*, *56*). The formulas used for the hyperfine coupling constants are $A=-\frac{\mu_{0}}{4\pi }\frac{\hbar \gamma_{S}\gamma_{I}}{r^{3}}\left[3{\text{cos}}^{2}\left(\beta \right)-1\right]$ and $B=-\frac{\mu_{0}}{4\pi }\frac{\hbar \gamma_{S}\gamma_{I}}{r^{3}}\frac{3}{2}\text{sin}\left(2\beta \right)$, where $\mu_{0}$ is the vacuum permeability, ℏ is the reduced Planck constant, $\gamma_{S}$ and $\gamma_{I}$ are gyromagnetic ratios for electron and proton, respectively, *r* is the electron-proton distance, and β is the polar angle between them. The MW irradiation $H_{\text{MW}}\left(t\right)$ was provided as a piecewise function using the amplitude, phase, and duration parameters for each experimental sequence. To translate the experimental MW amplitudes into frequencies, the maximal nutation frequency was measured and used as a reference for linear scaling. This maximal nutation was assumed constant for each optimization, but as seen previously from the nutation spectra, this is not always the case. (*57*) Accordingly, the simulations in Fig. 3 (E and F) used MW inhomogeneity averaging using the profile in table S17.

## Supplementary Materials

### This PDF file includes:

Supplementary Text Figs. S1 to S20 Tables S1 to S17 References

## References

[1] F. Bloch, Nuclear induction. Phys. Rev. 70, 460–474 (1946).

[2] E. L. Hahn, Spin echoes. Phys. Rev. 80, 580–594 (1950).

[3] N. F. Ramsey, A molecular beam resonance method with separated oscillating fields. Phys. Rev. 78, 695–699 (1950).

[4] N. A. Kurnit, I. D. Abella, S. R. Hartmann, Observation of a photon echo. Phys. Rev. Lett. 13, 567–568 (1964).

[5] J. H. Freed, Theory of saturation and double-resonance effects in ESR spectra. J. Chem. Phys. 43, 2312–2332 (1965).

[6] A. Abragam, *The Principles of Nuclear Magnetism*, International Series of Monographs on Physics (Clarendon Press, 1961).

[7] R. R. Ernst, G. Bodenhausen, A. Wokaun, *Principles of Nuclear Magnetic Resonance in One and Two Dimensions* (Oxford Univ. Press, 1990).

[8] A. Schweiger, G. Jeschke, *Principles of Pulse Electron Paramagnetic Resonance* (Oxford Univ. Press, 2001).

[9] U. Haeberlen, J. S. Waugh, Coherent averaging effects in magnetic resonance. Phys. Rev. 175, 453–467 (1968).

[10] M. Hohwy, N. C. Nielsen, Systematic design and evaluation of multiple-pulse experiments in nuclear magnetic resonance spectroscopy using a semi-continuous Baker–Campbell–Hausdorff expansion. J. Chem. Phys. 109, 3780–3791 (1998).

[11] T. S. Untidt, N. C. Nielsen, Closed solution to the Baker-Campbell-Hausdorff problem: Exact effective Hamiltonian theory for analysis of nuclear-magnetic-resonance experiments. Phys. Rev. E 65, 021108 (2002). 10.1103/PhysRevE.65.021108 11863504

[12] D. Siminovitch, T. Untidt, N. C. Nielsen, Exact effective Hamiltonian theory. II. Polynomial expansion of matrix functions and entangled unitary exponential operators. J. Chem. Phys. 120, 51–66 (2004). 15267261 10.1063/1.1628216

[13] R. Shankar, M. Ernst, P. K. Madhu, T. Vosegaard, N. C. Nielsen, A. B. Nielsen, A general theoretical description of the influence of isotropic chemical shift in dipolar recoupling experiments for solid-state NMR. J. Chem. Phys. 146, 134105 (2017). 28390347 10.1063/1.4979123

[14] A. B. Nielsen, M. R. Hansen, J. E. Andersen, T. Vosegaard, Single-spin vector analysis of strongly coupled nuclei in TOCSY NMR experiments. J. Chem. Phys. 151, 134117 (2019). 31594312 10.1063/1.5123046

[15] A. B. Nielsen, N. C. Nielsen, Accurate analysis and perspectives for systematic design of magnetic resonance experiments using single-spin vector and exact effective Hamiltonian theory. J. Magn. Reson. Open 12-13, 100064 (2022).

[16] N. Khaneja, T. Reiss, C. Kehlet, T. Schulte-Herbrüggen, S. J. Glaser, Optimal control of coupled spin dynamics: Design of NMR pulse sequences by gradient ascent algorithms. J. Magn. Reson. 172, 296–305 (2005). 15649756 10.1016/j.jmr.2004.11.004

[17] I. I. Maximov, Z. Tošner, N. C. Nielsen, Optimal control design of NMR and dynamic nuclear polarization experiments using monotonically convergent algorithms. J. Chem. Phys. 128, 184505 (2008). 18532824 10.1063/1.2903458

[18] Z. Tošner, T. Vosegaard, C. Kehlet, N. Khaneja, S. J. Glaser, N. C. Nielsen, Optimal control in NMR spectroscopy: Numerical implementation in SIMPSON. J. Magn. Reson. 197, 120–134 (2009). 19119034 10.1016/j.jmr.2008.11.020

[19] I. I. Maximov, J. Salomon, G. Turinici, N. C. Nielsen, A smoothing monotonic convergent optimal control algorithm for nuclear magnetic resonance pulse sequence design. J. Chem. Phys. 132, 084107 (2010). 20192290 10.1063/1.3328783

[20] D. L. Goodwin, I. Kuprov, Modified Newton-Raphson GRAPE methods for optimal control of spin systems. J. Chem. Phys. 144, 204107 (2016). 27250279 10.1063/1.4949534

[21] J. P. Carvalho, D. L. Goodwin, N. Wili, A. B. Nielsen, N. C. Nielsen, Optimal control design strategies for pulsed dynamic nuclear polarization. J. Chem. Phys. 162, 054111 (2025). 39902705 10.1063/5.0244723

[22] D. L. Goodwin, W. K. Myers, C. R. Timmel, I. Kuprov, Feedback control optimisation of ESR experiments. J. Magn. Reson. 297, 9–16 (2018). 30326343 10.1016/j.jmr.2018.09.009

[23] G. De Paëpe, P. Hodgkinson, L. Emsley, Improved heteronuclear decoupling schemes for solid-state magic angle spinning NMR by direct spectral optimization. Chem. Phys. Lett. 376, 259–267 (2003).

[24] A. Marshall, T. Reisser, P. Rembold, C. Müller, J. Scheuer, M. Gierse, T. Eichhorn, J. M. Steiner, P. Hautle, T. Calarco, F. Jelezko, M. B. Plenio, S. Montangero, I. Schwartz, M. M. Müller, P. Neumann, Macroscopic hyperpolarization enhanced with quantum optimal control. Phys. Rev. Res. 4, 043179 (2022).

[25] A. Doll, G. Jeschke, Wideband frequency-swept excitation in pulsed EPR spectroscopy. J. Magn. Reson. 280, 46–62 (2017). 28579102 10.1016/j.jmr.2017.01.004

[26] N. Wili, A. B. Nielsen, L. A. Völker, L. Schreder, N. C. Nielsen, G. Jeschke, K. O. Tan, Designing broadband pulsed dynamic nuclear polarization sequences in static solids. Sci. Adv. 8, eabq0536 (2022). 35857520 10.1126/sciadv.abq0536 PMC9286509

[27] A. B. Nielsen, J. P. A. Carvalho, D. L. Goodwin, N. Wili, N. C. Nielsen, Dynamic nuclear polarization pulse sequence engineering using single-spin vector effective Hamiltonians. Phys. Chem. Chem. Phys. 26, 28208–28219 (2024). 39498786 10.1039/d4cp03041a

[28] A. B. Nielsen, J. P. Carvalho, N. Wili, F. V. Jensen, D. L. Goodwin, T. S. Untidt, Z. Tošner, N. C. Nielsen, Controlling effective Hamiltonians: Broadband pulsed dynamic nuclear polarization by constrained random walk and non-linear optimization. J. Chem. Phys. 163, 144111 (2025). 41065154 10.1063/5.0287050

[29] J. Carvalho, A. B. Nielsen, D. L. Goodwin, N. Wili, N. C. Nielsen, Longitudinal pulsed dynamic nuclear polarization transfer via periodic optimal control. J. Phys. Chem. Lett. 17, 2517–2523 (2026). 41730128 10.1021/acs.jpclett.5c03708

[30] J. Stropp, N. Wili, N. C. Nielsen, D. Klose, Increased sensitivity in electron–nuclear double resonance spectroscopy with chirped radiofrequency pulses. Magn. Reson. 6, 33–42 (2025). 10.5194/mr-6-33-2025 PMC12247087 40657611

[31] N. Wili, A. B. Nielsen, J. P. Carvalho, N. C. Nielsen, Observation of dynamic nuclear polarization echoes. Sci. Adv. 10, eadr2420 (2024). 39423270 10.1126/sciadv.adr2420 PMC11488531

[32] MATLAB, *version 24.2.0 (R2024b)* (The MathWorks Inc., 2024).

[33] J. A. Nelder, R. Mead, A simplex method for function minimization. Comput. J. 7, 308–313 (1965).

[34] W. Press, S. Teukolsky, W. Vetterling, B. Flannery, *Numerical Recipes: The Art of Scientific Computing* (Cambridge Univ. Press, ed. 3, 2007).

[35] S. Kirkpatrick, C. D. Gelatt, M. P. Vecchi, Optimization by simulated annealing. Science 220, 671–680 (1983). 17813860 10.1126/science.220.4598.671

[36] Y. Jin, *Evolutionary Algorithms* (Physica-Verlag HD, 2003), pp. 49–71.

[37] D. C. Sorensen, Newton’s method with a model trust region modification. SIAM J. Numer. Anal. 19, 409–426 (1982).

[38] S. M. Goldfeld, R. E. Quandt, H. F. Trotter, Maximization by quadratic hill-climbing. Econometrica 34, 541–551 (1966).

[39] J. H. Ardenkjaer-Larsen, On the present and future of dissolution-DNP. J. Magn. Reson. 264, 3–12 (2016). 26920825 10.1016/j.jmr.2016.01.015

[40] G. Mathies, S. Jain, M. Reese, R. G. Griffin, Pulsed dynamic nuclear polarization with trityl radicals. J. Phys. Chem. Lett. 7, 111–116 (2016). 26651876 10.1021/acs.jpclett.5b02720 PMC4935761

[41] A. Henstra, P. Dirksen, J. Schmidt, W. Wenckebach, Nuclear spin orientation via electron spin locking (NOVEL). J. Magn. Reson. 77, 389–393 (1988).

[42] S. K. Jain, G. Mathies, R. G. Griffin, Off-resonance NOVEL. J. Chem. Phys. 147, 164201 (2017). 29096491 10.1063/1.5000528 PMC5659863

[43] K. O. Tan, C. Yang, R. T. Weber, G. Mathies, R. G. Griffin, Time-optimized pulsed dynamic nuclear polarization. Sci. Adv. 5, eaav6909 (2019). 30746482 10.1126/sciadv.aav6909 PMC6357739

[44] V. S. Redrouthu, G. Mathies, Efficient pulsed dynamic nuclear polarization with the X-inverse-X sequence. J. Am. Chem. Soc. 144, 1513–1516 (2022). 35076217 10.1021/jacs.1c09900

[45] V. S. Redrouthu, S. Vinod-Kumar, G. Mathies, Dynamic nuclear polarization by two-pulse phase modulation. J. Chem. Phys. 159, 014201 (2023). 37403849 10.1063/5.0153053

[46] S. A. Jegadeesan, Y. Zhao, G. M. Smith, I. Kuprov, G. Mathies, Simulation of pulsed dynamic nuclear polarization in the steady state. J. Chem. Phys. 163, 034111 (2025). 40667727 10.1063/5.0283196

[47] I. Schwartz, J. Scheuer, B. Tratzmiller, S. Müller, Q. Chen, I. Dhand, Z.-Y. Wang, C. Müller, B. Naydenov, F. Jelezko, M. B. Plenio, Robust optical polarization of nuclear spin baths using Hamiltonian engineering of nitrogen-vacancy center quantum dynamics. Sci. Adv. 4, eaat8978 (2018). 30182060 10.1126/sciadv.aat8978 PMC6118411

[48] P. I. Frazier, A tutorial on Bayesian optimization. arXiv:1807.02811 [stat.ML] (2018).

[49] S. Greenhill, S. Rana, S. Gupta, P. Vellanki, S. Venkatesh, Bayesian optimization for adaptive experimental design: A review. IEEE Access 8, 13937–13948 (2020).

[50] C. E. Rasmussen, C. K. I. Williams, *Gaussian Processes for Machine Learning* (The MIT Press, 2005).

[51] J. Snoek, H. Larochelle, R. P. Adams, “Practical Bayesian optimization of machine learning algorithms” in *Proceedings of the 26th International Conference on Neural Information Processing Systems—Volume 2, NIPS'12*. (Curran Associates Inc., 2012), pp. 2951–2959.

[52] B. Letham, B. Karrer, G. Ottoni, E. Bakshy, Constrained Bayesian optimization with noisy experiments. Bayesian Anal. 14, 495–519 (2019).

[53] H. B. Moss, D. S. Leslie, J. Gonzalez, P. Rayson, GIBBON: General-purpose information-based Bayesian optimisation. J. Mach. Learn. Res. 22, 1–49 (2021).

[54] M. Binois, N. Wycoff, A survey on high-dimensional gaussian process modeling with application to bayesian optimization. ACM Trans. Evol. Learn. Optim. 2, 1–26 (2022).

[55] J. P. Carvalho, A. B. Nielsen, E. Baligács, N. Wili, N. C. Nielsen, Bridging dynamic nuclear polarization and solid-state NMR dipolar recoupling: From static single crystal to spinning powders. J. Phys. Chem. Lett. 16, 4363–4371 (2025). 40272255 10.1021/acs.jpclett.5c00617

[56] D. L. Goodwin, J. P. Carvalho, A. B. Nielsen, N. Wili, A. Brinkmann, T. Vosegaard, Z. Tošner, N. C. Nielsen, Extending numerical simulations in SIMPSON: Electron paramagnetic resonance, dynamic nuclear polarisation, propagator splitting, pulse transients, and quadrupolar cross terms. J. Magn. Reson. Open 27, 100218 (2026).

[57] R. Gupta, G. Hou, T. Polenova, A. J. Vega, RF inhomogeneity and how it controls CPMAS. Solid State Nucl. Magn. Reson. 72, 17–26 (2015). 26422256 10.1016/j.ssnmr.2015.09.005 PMC4674349

[58] Matlab documentation on Bayesian Optimization Algorithm; https://se.mathworks.com/help/stats/bayesian-optimization-algorithm.html#bvaz8tr-1.

[59] A. D. Bull, Convergence rates of efficient global optimization algorithms. J. Mach. Learn. Res. 12, 2879–2904 (2011).

[60] M. A. Gelbart, J. Snoek, R. P. Adams, Bayesian optimization with unknown constraints. arXiv:1403.5607 [stat.ML] (2014).

