# Supplementary material for: In situ learning-based spin engineering of pulsed dynamic nuclear polarization
Source: Sci Adv. 2026 Sep 16;12(38):eaeh4467. doi: 10.1126/sciadv.aeh4467 (PMC13580563; doi:10.1126/sciadv.aeh4467)
Supplement: Supplementary file 1 [file sciadv.aeh4467_sm.pdf]

Science Advances
AAAS

Supplementary Materials for

In situ learning-based spin engineering of pulsed dynamic nuclear polarization

Filip V. Jensen *et al.*

Corresponding author: Anders Bodholt Nielsen, abn@chem.au.dk; Niels Chr. Nielsen, ncn@chem.au.dk

Sci. Adv. **12**, eaeh4467 (2026)
DOI: 10.1126/sciadv.aeh4467

This PDF file includes:

Supplementary Text
Figs. S1 to S20
Tables S1 to S17
References

Supplementary Text

The following supplementary text provides more detailed information on the implementation of the Bayesian optimization integrated with the home-built pulsed DNP spectrometer (see main text) via the feedback-loop control scheme in Fig. 5 (main text) and the constrained random walk (cRW) (*28*) used to constrain the Bayesian operational space. Furthermore, the supplementary materials (text, figures, and tables) provide details on calibrations, optimizations, parameters, and supplementary numerical Bayesian optimizations (*in silico*, mimicking *in situ* experimental procedures). The MATLAB (*32*) code used for *in silico* Bayesian optimization is also provided.

1 Bayesian implementation for DNP optimizations

This section describes the Bayesian procedures, including variable definitions and the options for cRW constraints. The options are integral in the provided Bayesian optimization code and descriptive for the performed experimental and numerical optimizations as described in Supplementary Figures and Supplementary Tables.

1.a Bayesian algorithm in Matlab

To perform Bayesian optimization for efficient DNP transfer, we have in this study used the Bayesian optimization package `bayesopt` implemented in the Matlab programming environment. (*32*) The Bayesian was implemented with the following instruction:

```
bayesopt(objectiveFunction ...
    ,variableDescriptions ...
    ,'AcquisitionFunctionName',options.acFunc ...
    ,'IsObjectiveDeterministic',options.deterministic ...
    ,'NumSeedPoints',4 ...
    ,'MaxObjectiveEvaluations',MaxEvals);
```

where the arguments (in red) and general settings for the Bayesian optimization are defined below.

Defining `objectiveFunction` in MATLAB for DNP optimization

The objective function for the Bayesian optimization is developed to enable communication with the spectrometer via the feedback-loop control procedure in Fig. 5 (main text). It receives control variables x from `bayesopt`, runs the experiments, saves the experiment settings and results to the experiment log, and calculates the absolute mean of enhancements.

```
objectiveFunction = @(x) -abs(mean(bayesopt_wrapper(x,options)));
```

inside `bayesopt_wrapper` the control variables are used to determine the amplitude and phase modulations of the MW irradiation based on **`options`**. It then runs the pulse sequence for the chosen offsets and outputs DNP enhancements as a vector

```
function [enhancements] = bayesopt_wrapper(x,options)
% Determine DNP sequence from variables
[excitationSeq,transferSeq] = DNP_sequence(x,options);
% Run DNP experiments and save to file
filepath = run_DNP(excitationSeq,transferSeq,options);
% Evaluate enhancements
reference = process_reference();
enhancements = get_DNP_enhancement(filepath,reference);
end
```

Definition of `options` in MATLAB for DNP optimization

The implementation of `bayesopt` is controlled via a set of **`options`**. Here a short explanation for each option is given, and in Tables S1 to S5, S8 to S10 and S12 to S16, the settings of these options are listed for each of

the presented optimizations.

`options.acFunc` specifies the acquisition function Ac used in the Bayesian optimization. `bayesopt` has 4 built-in acquisition functions, discounting an acquisition function based on evaluation time: "expected-improvement", "expected-improvement-plus", "lower-confidence-bound" and "probability-of-improvement" as described in more detail in the underlying Matlab documentation. (*32, 58–60*) If acFunc is specified as “monte-carlo”, it implies that a Monte Carlo optimization was used instead of the Bayesian.

`options.deterministic` specifies if the objective function is deterministic. If set to "false", noise σ is included in the Gaussian process model. This option was set to "true" for numerical optimizations and "false" for experimental optimizations. Exceptions include the experimental optimizations in Figs. 2A-C and S8, in which the objective function was treated as deterministic. One may, in retrospect, argue if this setting was optimal, but the low noise of the experimental model implies that this initial choice should not make a notable difference.

`options.nu1max` is the maximal MW amplitude $\omega_1/(2\pi)$ in MHz. For the experimental models, this value was determined from the resonator profile as shown in Fig. S13.

`options.nuI` is the nuclear Larmor $\omega_I/(2\pi)$ frequency in MHz.

`options.eOffsets` are the evaluated electron-spin offsets $\Delta\omega_S/(2\pi)$ in MHz. `bayesopt_wrapper` will output an enhancement for each of these offsets.

`options.transPulseNum` is the number of pulses in the transfer element.

`options.transAmpScale` is the range of possible MW amplitudes for pulses in a transfer element in units of maximal nutation frequency $\omega_{max}/(2\pi)$ before potential constraints are taken into account.

`options.transPhase` is the phase ϕ of the pulses in a transfer element in radians. If a range is specified, `bayesopt` optimizes the phase of each pulse individually within the given range.

`options.transPulseTime` is the duration of each pulse in a transfer element in ns.

`options.RWorder` is the order in which the accumulated nutation angles θ of the transfer element are fixed. "forward" fixes the angles in chronological order, "backtrack" fixes the angles in reverse order, and "factorize" fixes the angle that is most distant in time to currently fixed angles. These aspects are discussed in

Supplementary Text Section 1.b and visualized in Fig. S1.

options.`constrained` specifies if the transfer elements are constrained by a resonance condition. If set to "false", the Bayesian optimization will freely vary pulse amplitudes, phases and durations within the allowed ranges. If set to "true", the Bayesian will instead vary the parameters of a cRW and construct the transfer element as explained in Section 1.b. In the present work, only constraints for optimization of transfer elements on trityl OX063 were implemented. In principle other constraints could be derived and implemented in similar fashion.

options.`DNPres` is the DNP resonance chosen for the potential constraint, specified by "ZQ" or "DQ" and the value of k_I.

options.`DNPoffset` is the angular offset in degrees of the accumulated nutation angle with respect to the chosen DNP resonance. If a range is specified, the Bayesian optimization decides how close the transfer element should be to the resonance within the given range.

The following **options** are specific to the numerical simulations:

options.`excitation` defines amplitudes, phases and durations of the excitation sequence. It can be set to "ideal" to circumvent the initial excitation pulse and instead initialize the density matrix as S_x.

options.`maxContact` is the maximal contact time for the simulated transfer in ns. The simulation repeats the transfer element at zero electron-spin offset until a maximal proton polarization is found or until the maximal contact time is expended.

options.`epDist` is the distance between the electron and proton in Å.

options.`polAngles` is the number of polar angles used in the powder average, distributed equally from 0 to $\pi/2$ radians but omitting 0.

options.`MWinhom` specifies if the simulation should use MW amplitude inhomogeneity averaging given in Table S17.

The following **options** are specific to the experimental implementation:

options.B0 is the static magnetic field in T.

options.nuS is carrier frequency for the maximum of the electron resonance spectrum.

options.NMRpulseTime is the duration of the $\pi/2$ pulse in the solid-echo. In addition, the duration of the saturation pulses is set to 1.2 times this duration.

options.excitePulseNum is the number of pulses in the excitation sequence.

options.exciteAmpScale is the MW amplitude of the excitation pulses in units of maximal nutation frequency. If a range is specified, bayesopt optimizes the amplitude of each pulse individually within the given range.

options.excitePhase is the phase of the excitation pulses. If a range is specified, bayesopt optimizes the phase of each pulse individually within the given range.

options.excitePulseTime is the duration of each excitation pulse. If a range is specified, bayesopt optimizes the duration within the given range with all pulses having the same duration.

options.transRepeat is the number of repetitions of the transfer element within a single DNP contact. If a range is specified, bayesopt optimizes the number of repetitions within the given range.

options.transContact is the total contact time of the transfer sequence in ns and can be specified instead of **options**.transPulseTime. In this case the duration of the transfer pulses is set such that the contact time comes closest to the specified value with the given number of repetitions. If a range is specified, bayesopt optimizes the contact time within the given range.

options.buildup is the DNP buildup time t_{DNP} for a single DNP experiment.

It is noted that in all cases where pulse durations were varied, the result was rounded to the nearest 0.125 ns, since this is the minimal digitization for the AWG (see Materials and Methods, main text) set to a 8 GHz sampling rate.

Defining variableDescriptions in MATLAB for feedback-loop *in situ* DNP optimization

To run bayesopt a set of **variableDescriptions** have to be defined as a vector of the built-in function

`optimizableVariable(variableName,range)`. It defines which values are allowed for each variable in x and will therefore have the same number of elements. Its structure and ranges will, however, depend on the optimization setup.

If the contact time was set to be optimized, `optimizableVariable("t_contact",options.transContact)` was used as part of **`variableDescriptions`**. Likewise, if the repetitions of the transfer element were to be optimized, `optimizableVariable("n_rep",options.transRepeat,"Type","integer")` was used as part of **`variableDescriptions`**, where the `"Type","integer"` input specifies that the number of repetitions must be an integer. The rest of **`variableDescriptions`** describe the amplitudes and phases of the excitation and transfer pulses in the extent they were to be optimized.

For Bayesian optimizations without a resonance constraint, **`variableDescriptions`** is just defined as a vector containing the variables and their range defined by **`options`**. In this case the structure of the input vector x will be

$$x = \left[\omega_1^{(ex,1)}/\omega_{max}, \phi^{(ex,1)}, ..., \omega_1^{(tr,1)}/\omega_{max}, \phi^{(tr,1)}, ..., t_{contact}, n_{rep}\right]$$

where $\omega_1/(2\pi)$ is the MW nutation frequency, ϕ the phase, t_{contact} the contact time, and n_{rep} the number of transfer element repetitions. The superscripts (ex, i) and (tr, i) signify the i'th excitation and transfer pulse, respectively.

1.b Constrained random walk

When the transfer element was constrained by a resonance condition, the **`variableDescriptions`** were instead used for a cRW. The cRW (*28*) describes the progression of accumulated nutation angles $\theta_0, ..., \theta_p$ at time point $t_0, ..., t_p$, where p is the number of pulses in the transfer element, $t_0 = 0$ and $\Delta t = t_{i+1} - t_i$ is the duration of a single pulse, which was in our case chosen to be constant. When the cRW has been determined, the corresponding nutation frequencies of the pulses can be calculated using a difference quotient

$$\omega_1^{(tr,i)} = \frac{\theta_i - \theta_{i-1}}{\Delta t} \quad \text{for} \quad i \in \{1, \ldots, p\} \quad . \tag{S1}$$

To construct the cRW, the accumulated nutation angles have to obey the constraints of maximal MW nutation frequency, ω_{max}, and the DNP resonance,

$$\theta_0 = 0 \quad , \tag{S2}$$

$$\left|\frac{\theta_i - \theta_j}{t_i - t_j}\right| \leq \omega_{max} \quad \text{for} \quad i \neq j \in \{0, \ldots, p\} \quad , \tag{S3}$$

$$\left|\theta_p - \theta^{\text{DNP}}\right| \leq \Delta \quad , \tag{S4}$$

where θ^{DNP} is given by the resonance condition in Eq. (1) and Δ is the maximal angular offset from resonance.

Initially, only θ_0 is fixed while the rest of the angles can be set within a range

$$\theta_i = (\theta_{i,max} - \theta_{i,min})x_i + \theta_{i,min} \quad , \tag{S5}$$

where $x_i \in [0, 1]$ is the i'th variable in x and $\theta_{i,max}$ and $\theta_{i,min}$ are the maximal and minimal allowed angle for θ_i, respectively.

The allowed values for θ_i will depend on the value of nearby angles θ_j that have already been fixed as described by Eq. (S3). Therefore, the resulting cRW, and consequently the pulse sequence, will depend on the order in which the angles θ_i are set. This is illustrated in Fig. S1 for the three different orders used in the present publication. "forward" fixes the angles in chronological order, "backtrack" fixes the angles in reverse order, and "factorize" fixes the angle that is most distant in time to the currently fixed angles. To calculate the allowed values for θ_i, only the closest fixed angle to the left $\theta_{L(i)}$ and to the right $\theta_{R(i)}$ is needed

$$\theta_{i,max} = \min\left\{\theta_{L(i)} + \omega_{max}(t_i - t_{L(i)}),\ \theta_{R(i)} + \omega_{max}(t_{R(i)} - t_i)\right\} \tag{S6}$$

$$\theta_{i,min} = \max\left\{\theta_{L(i)} - \omega_{max}(t_i - t_{L(i)}),\ \theta_{R(i)} - \omega_{max}(t_{R(i)} - t_i)\right\} \quad . \tag{S7}$$

If there are no fixed angles to the right of θ_i, Eq. (S4) must instead be taken into account

$$\theta_{i,max} = \min\left\{\theta_{L(i)} + \omega_{max}(t_i - t_{L(i)}),\ \theta_{DNP} + \Delta + \omega_{max}(t_p - t_i)\right\} \tag{S8}$$

$$\theta_{i,min} = \max\left\{\theta_{L(i)} - \omega_{max}(t_i - t_{L(i)}),\ \theta_{DNP} - \Delta - \omega_{max}(t_p - t_i)\right\} \quad . \tag{S9}$$

To illustrate how this works, Fig. S2 walks through the process of constructing the cRW shown in Fig. S1C. From Eqs. (S5-S9), the accumulated nutation angles can be determined from the input vector x and the **options** which then gives the pulse sequence by Eq. (S1). In the end the structure of the input vector x for optimization of a p-pulse constrained transfer element was

$$x = \left[x_1, ..., x_p, n_{\text{rep}}\right] \quad ,$$

where the n_{rep} variable is added only if the number of repetitions is being optimized.

The reason for defining these alternative cRW orders is that the straightforward approach of fixing the angles in chronological order often results in a final nutation angle near the boundaries of the DNP resonance in Eq. (S4). The effect of choosing between the different RW orders and the different acquisition functions has been investigated numerically in Fig. S16. From this figure, it was found that the "factorize" cRW order performed best and for the optimization in Fig. 2E (main text), this order was selected along with the acquisition function "expected-improvement-plus".

1.c Bayesian optimization MATLAB code

In this section, we provide the Matlab code used for *in silico* Bayesian optimization, noting that the corresponding experimental code depends specifically on the software steering our home-built pulsed DNP/EPR spectrometer and as such is not relevant to deploy generally. The presented code, however, illustrates all relevant aspects of the Bayesian optimization.

- Optimization setup script.

```
%% Physical inputs
options.nuI = 14.8; % nuclear zeeman
options.nu1max = 32; % maximal irradiation strength
options.epDist=4.5; % proton-electron distance in Angstrom
%% Orientations and offsets
options.polarAngles=20; % Number of orientations from 0 to pi/2
options.eOffsets = -60:0.5:60; % electron offsets to be analyzed
options.MWinhom = false;
%% Flip definitions
```

```
options.excitation = 'ideal';
%% Transfer definitions
options.transPulseNum = 24;
options.transPulseTime = 5;
options.maxContact = 10e3;
options.DNPres = {'DQ',2};
options.DNPoffset = 5;
options.RWorder = "forward";
%% bayesopt options
options.aqFunc = "expected-improvement-plus";
MaxEvals = 1000;
rng('shuffle')
%% Optimization
variableDescriptions = [];
for i = 1:options.transPulseNum
    variableDescriptions = [variableDescriptions,optimizableVariable...
    (['x' num2str(i)],[0,1])];
end
objectiveFunction = @(x) -abs(mean(numeric_bayesopt_wrapper(x,options)));
inputs = {objectiveFunction,variableDescriptions ...
    ,'AcquisitionFunctionName',options.aqFunc ...
    ,'IsObjectiveDeterministic',true ...
    ,'MaxObjectiveEvaluations',MaxEvals ...
    ,'Verbose',1 ...
    ,'PlotFcn',{} ...
    ,'OutputFcn',@saveToFile ...
    };
bayesopt(inputs{:});
```

- Objective function for numerical implementation.

```
function [output] = numeric_bayesopt_wrapper(x,options)
if istable(x)
    x = table2array(x);
end
if strcmp(options.excitation,'ideal')
    flip = zeros(3,0);
else
    flip = options.excitation;
end
transfer = construct_pulse_sequence(x,options);
output = numeric_DNP(flip,transfer,options);
end
```

- Code for calculating transfer element.

```
function [transfer] = transfer_element(x,options)
transfer = zeros(3,options.transPulseNum);
%% Determine pulsetime
transfer(3,:) = options.transPulseTime * ones(1,options.transPulseNum);
%% Find matching
effrot = options.nuI*sum(transfer(3,:))/1000;
if strcmp(options.DNPres{1},'DQ')
    thetaDNP = 2*pi*(effrot-options.DNPres{2});
elseif strcmp(options.DNPres{1},'ZQ')
    thetaDNP = -2*pi*(effrot-options.DNPres{2});
else
    error([options.DNPres{1} ' is not a valid transfer.'])
end
if abs(thetaDNP)-options.DNPoffset*pi/180>2*pi*options.nu1max*sum(transfer(3,:))
    error('requested overall flip angle physically impossible. lower kIa and/or change ZQ/DQ')
end
%% Generate cRW
theta = generate_cRW(x,thetaDNP+options.DNPoffset*pi/180*[-1,1],transfer(3,:),options.nu1max,options.RWorder,[-Inf,Inf]);
%% Calculate pulse sequence
amps = diff(theta)/(2*pi)./transfer(3,:)*1000/options.nu1max;
amps(isnan(amps)) = 0;
transfer(1,:) = abs(amps);
transfer(2,:) = pi*(1-sign(amps))/2;
end
```

- Code for constructing cRW.

```
    function [theta] = generate_cRW(x,target,pulsetimes,maxfreq,RWorder,MinMax)
%Convert from nanoseconds to microseconds
pulsetimes = pulsetimes/1000;
if isscalar(maxfreq)
    posfreq = maxfreq;
    negfreq = maxfreq;
else
    posfreq = maxfreq(2);
    negfreq = maxfreq(1);
end
%% Checks
if isscalar(target)
    target = [target,target];
end
if nargin < 5
    RWorder = "forward";
end
if nargin < 6
    MinMax = [-Inf,Inf];
```

```
end
if target(1) > target(2)
    error(['The␣target␣range␣[' num2str(target(1)) ',' num2str(target(2)) ']␣must␣be␣increasing.' ])
end
if MinMax(1) > MinMax(2)
    error(['The␣bounds␣[' num2str(MinMax(1)) ',' num2str(MinMax(2)) ']␣must␣be␣increasing.' ])
end
if MinMax(1) > 0 || MinMax(2) < 0
    error(['The␣starting␣point␣0␣must␣be␣within␣the␣bounds:␣[' num2str(MinMax(1)) ',' num2str(MinMax(2)) ']'])
end
if target(2) < MinMax(1) || target(1) > MinMax(2)
    error(['Cannot␣reach␣target␣range␣of␣[' num2str(target(1)) ','...
    num2str(target(2)) ']␣within␣bounds:␣[' num2str(MinMax(1)) ',' num2str(MinMax(2)) ']'])
end
if target(2) < -2*pi*sum(pulsetimes)*negfreq || target(1) > 2*pi*sum(pulsetimes)*posfreq
    error(['Cannot␣reach␣target␣range␣of␣[' num2str(target(1)) ',' ...
    num2str(target(2)) ']␣rad␣within␣' num2str(sum(pulsetimes)) '\mus␣with␣a␣maximal␣nutation␣of␣'...
 num2str(negfreq) '/' num2str(posfreq) '␣MHz'])
end
%% Picking flip angles during the pulse sequence consistent with experiment options
pulsenum = length(pulsetimes);
theta = zeros(1,pulsenum+1);
maxi = MinMax(2)*ones(1,pulsenum);
mini = MinMax(1)*ones(1,pulsenum);
switch RWorder
    case 'forward'
        for j = 1:pulsenum
            maxi(j) = min([theta(j)+2*pi*posfreq*pulsetimes(j),target(2)+2*pi*negfreq*sum(pulsetimes(j+1:end)),maxi(j)]);
            mini(j) = max([theta(j)-2*pi*negfreq*pulsetimes(j),target(1)-2*pi*posfreq*sum(pulsetimes(j+1:end)),mini(j)]);
            theta(j+1) = mini(j) + x(j)*(maxi(j)-mini(j));
        end
    case 'backtrack'
        maxi(end) = min([2*pi*posfreq*sum(pulsetimes),target(2),maxi(end)]);
        mini(end) = max([-2*pi*negfreq*sum(pulsetimes),target(1),mini(end)]);
        theta(end) = mini(end) + x(end)*(maxi(end)-mini(end));
        for j = fliplr(1:pulsenum-1)
            maxi(j) = min([theta(j+2)+2*pi*posfreq*pulsetimes(j+1),2*pi*negfreq*sum(pulsetimes(1:j)),maxi(j)]);
            mini(j) = max([theta(j+2)-2*pi*negfreq*pulsetimes(j+1),-2*pi*posfreq*sum(pulsetimes(1:j)),mini(j)]);
            theta(j+1) = mini(j) + x(j)*(maxi(j)-mini(j));
        end
    case 'factorize'
        count = 1;
        maxi(end) = min([2*pi*posfreq*sum(pulsetimes),target(2),maxi(end)]);
        mini(end) = max([-2*pi*negfreq*sum(pulsetimes),target(1),mini(end)]);
        theta(end) = mini(end) + x(end)*(maxi(end)-mini(end));
        factors = factor(pulsenum);
```

```
        product = [1,cumprod(factors)];
        for j = 1:length(factors)
            secsize = pulsenum/product(j);
            partsize = secsize/factors(j);
            for sec = 1:product(j)
                endindex = secsize*sec+1;
                for part = 1:factors(j)-1
                    startindex = secsize*(sec-1)+(part-1)*partsize+1;
                    pulseindex = secsize*(sec-1)+part*partsize;
                    maxi(pulseindex) = min([theta(startindex) + 2*pi*posfreq*sum(pulsetimes(startindex:pulseindex)), ...
                        theta(endindex) + 2*pi*negfreq*sum(pulsetimes(pulseindex+1:endindex-1)), ...
                        maxi(pulseindex)]);
                    mini(pulseindex) = max([theta(startindex) - 2*pi*negfreq*sum(pulsetimes(startindex:pulseindex)), ...
                        theta(endindex) - 2*pi*posfreq*sum(pulsetimes(pulseindex+1:endindex-1)), ...
                        mini(pulseindex)]);
                    theta(pulseindex+1) = mini(pulseindex) + x(pulseindex)*(maxi(pulseindex)-mini(pulseindex));
                    count = count+1;
                end
            end
        end
    otherwise
        error(['Error in pulse sequence construction: ' RWorder ' is not a valid amplitude type.'])
end
end
```

- Code for numerical simulation of DNP on a 2-spin system.

```
function protonPolarization = numeric_DNP(flip,transfer,options)
%% Set polar angles and MW inhomogeneity
if options.polarAngles == 1
    pol_ang = pi/4;
else
    pol_ang = linspace(0,pi/2,options.polarAngles+1);
    pol_ang(1) = [];
end
if options.MWinhom
    inhom = [0.65 0.0484168
        0.70 0.052932
        0.75 0.0586464
        0.80 0.0662188
        0.85 0.0769463
        0.90 0.0938953
        0.95 0.12731
        1.00 0.32881
        1.05 0.146825];
else
```

```
    inhom = [1,1];
end
%% Physical operators and constants
X = 0.5*[0,1;
    1,0];
Y = 0.5i*[0,-1;
    1,0];
Z = 0.5*[1,0;
    0,-1];
Sx = kron(X,eye(2));
Sy = kron(Y,eye(2));
Sz = kron(Z,eye(2));
Ix = kron(eye(2),X);
Iz = kron(eye(2),Z);
if strcmp(options.excitation,'ideal')
    flip = zeros(3,0);
    rho0 = Sx; % Set starting state
else
    rho0 = Sz; % Set starting state
end
detect = Iz; % Set detection operator
mu0=1.256637062120000e-06;
gfree = 2.002319304362560;
bmagn = 9.274010078300000e-24;
g1H = 5.585694680000000;
planck = 6.626070150000000e-34;
nmagn = 5.050783746100000e-27;
T = mu0/(4*pi)*gfree*bmagn*g1H*nmagn*1/(options.epDist*1e-10)^3/planck/1e6;
%% Run flip and prepare transfer for zero offset
% scaling for different orientations
weights=sin(pol_ang); weights=weights/sum(weights);
buildup = 0;
rho = cell(options.polarAngles,size(inhom,1));
rho(:) = {rho0};
Uround = cell(options.polarAngles,size(inhom,1));
Uround(:) = {eye(size(Sz))};
for i = 1:options.polarAngles
    % Nuclear Larmor term and hyperfine coupling
    A=T*(3*cos(pol_ang(i))^2-1);
    B=3*sin(pol_ang(i))*cos(pol_ang(i))*T;
    H0 = options.nuI*Iz+A*Sz*Iz+B*Sz*Ix;
    for j = 1:size(inhom,1)
        for ip = 1:size(flip,2) % Run flip pulse sequence
            H1 = flip(1,ip)*inhom(j,1)*options.nu1max*( Sx*cos(flip(2,ip))+Sy*sin(flip(2,ip)) );
            % propagator for flip pulse
            Uflip = expm(-1i*2*pi*(H0+H1)*flip(3,ip)*1e-3);
```

```
            rho{i,j} = Uflip*rho{i,j}*Uflip';
        end
        buildup=buildup + weights(i)*inhom(j,2)*real(trace(detect*rho{i,j})/trace(detect*detect));
        % propagator for transfer element
        for ip = 1:size(transfer,2)
            H1 = transfer(1,ip)*inhom(j,1)*options.nu1max*( Sx*cos(transfer(2,ip))+Sy*sin(transfer(2,ip)) );
            Uround{i,j} = expm(-1i*2*pi*(H0+H1)*transfer(3,ip)*1e-3)*Uround{i,j};
        end
    end
end
count = 0;
while ~exist('nrep')
    count = count+1;
    buildup = [buildup,0];
    for i = 1:options.polarAngles
        for j = 1:size(inhom,1)
            rho{i,j} = Uround{i,j}*rho{i,j}*Uround{i,j}';
            buildup(end) = buildup(end) + weights(i)*inhom(j,2)*real(trace(detect*rho{i,j})/trace(detect*detect));
        end
    end
    if abs(buildup(end)) < buildup(end-1)
        nrep = count-1;
    elseif (count+1)*sum(transfer(3,:)) > options.maxContact
        [~,count] = max(abs(buildup));
        nrep = count-1;
    end
end
%% Run offset flip and transfer
profiles = zeros(length(options.eOffsets),nrep+1);
offzero = find(options.eOffsets == 0);
profiles(offzero,:) = buildup(1:nrep+1);
offind = 1:length(options.eOffsets);
offind(offzero) = [];
for i = 1:options.polarAngles
    % Nuclear Larmor and hyperfine coupling
    A=T*(3*cos(pol_ang(i))^2-1);
    B=3*sin(pol_ang(i))*cos(pol_ang(i))*T;
    H0 = options.nuI*Iz+A*Sz*Iz+B*Sz*Ix;
    for j = 1:size(inhom,1)
        for o = offind
            rho = rho0; % Set starting state
            for ip = 1:size(flip,2) % Run flip pulse sequence
                H1 = options.eOffsets(o)*Sz ...
                    + flip(1,ip)*inhom(j,1)*options.nu1max*( Sx*cos(flip(2,ip))+Sy*sin(flip(2,ip)) );
                % propagator for flip pulse
                Uflip = expm(-1i*2*pi*(H0+H1)*flip(3,ip)*1e-3);
```

```
                rho = Uflip*rho*Uflip';
            end
            % propagator for transfer element
            Uround = eye(size(Sz));
            for ip = 1:size(transfer,2)
                H1 = options.eOffsets(o)*Sz ...
                    + transfer(1,ip)*inhom(j,1)*options.nu1max*( Sx*cos(transfer(2,ip))+Sy*sin(transfer(2,ip)) );
                Uround = expm(-1i*2*pi*(H0+H1)*transfer(3,ip)*1e-3)*Uround;
            end
            for istep = 1:nrep
                profiles(o,istep)=profiles(o,istep) + weights(i)*inhom(j,2)*real(trace(detect*rho)/trace(detect*detect));
                rho = Uround*rho*Uround';
            end
            profiles(o,end)=profiles(o,end) + weights(i)*inhom(j,2)*real(trace(detect*rho)/trace(detect*detect));
        end
    end
end
protonPolarization = profiles(:,end)';
end
```

2 Description of supplementary numerical optimizations

To investigate the performance of various implementations of Monte Carlo and Bayesian pulse sequence optimizations, we conducted a series of *in silico* optimizations as illustrated in Figs. S6, S7 and S16.

2.a *In silico* pulse sequence optimizations

More specifically, to compare with *in situ* experimental optimizations and investigate the influence of different optimization procedures and number of pulses, we conducted the following *in silico* optimizations. These were typically faster than the experimental optimizations and provided more direct information on specific determinants in the optimizations.

- Fig. S6: Comparison of different optimization methods for DNP with 6-pulse elements to compare directly with the *in situ* optimizations in Fig. 2 (main text). Qualitatively the *in silico* and *in situ* optimizations display similar trends, however, with the difference that the numerically optimized sequences

are less broadbanded which may ascribed partly to that the *in situ* optimizations automatically folds with the lineshape of the EPR line of trityl OX063. We note that the cRW-OPT pulse sequences (*28*) numerically were better in this respect. The parameters used for the optimizations are given in Table S3.

- Fig. S7: Comparing cRW constrained Bayesian optimization with different numbers of pulses. As Bayesian optimization, in terms of speed and needed degrees of optimization freedom, depends on the number of variables in the optimization, we explored *in silico* the performance of 1000 constrained Bayesian evaluations for cases of 6, 12, and 24 pulses. Inspecting, the transfer efficiency integrated over offset (as in Fig. 2, main text), it appears that 24 pulses challenge convergence while both 6 and 12 pulses provide reasonable efficiencies and offset profiles. We note that the experimental evaluation samples a larger spin system and more instrumental errors than included in the numerical (included MW inhomogeneity, see below) mimic. The parameters used for the optimizations are given in Table S4.

- Fig. S16: Exploration of different acquisition functions and cRW settings. From this analysis, we established optimal parameters for the *in situ* optimizations as discussed in Supplementary Text Section 1.a. The parameters used for the optimizations are given in Tables S13 to S16.

2.b MW inhomogeneity profile

To reproduce the best possible experimental conditions by numerical simulations, the simulations in Fig. 3E-F take into account an MW inhomogeneity profile as listed in Table. S17. The specific profile was established in a power model as described by Gupta et al. (*57*)

3 Description of supplementary experimental data

To support the experimental data for Monte Carlo and Bayesian optimization, we present here supplementary data in form of calibrations, parameter evaluations, and additional optimizations.

3.a Calibrations and background experiments

To conduct *in situ* optimization of broadband DNP experiments and compare with reference experiments, we initially calibrated parameters for the investigated samples on the DNP spectrometer. The parameters also served as input to numerical simulations.

- Fig. S13: Nutation curves (in time and frequency domain) and resonator profiles recorded as part of optimization of experimental parameters.
- Fig. S14: EPR resonance profiles recorded by field sweep for the two trityl OX063 samples with difference solvent deuteration, as well as for 4-Oxo-TEMPO.
- Fig. S15: Proton and electron T_1 relaxation profiles for the two trityl OX063 samples and 4-Oxo-TEMPO recorded as part of optimization of experimental parameters.

3.b Pulse sequence optimizations and pulse sequence analysis

Pulse sequence optimizations involved:

- Fig. S3: Experimental offset profiles recorded for trityl OX063 in glycerol-d_5 for the Bayesian optimized DNP transfer pulse sequence in Fig. 2E relative to NOVEL, PLATO, and cRW-OPT1 DNP transfers (to compare with Fig. 2F using glycerol-d_8 in the solvent).
- Fig. S4: Analysis of the distribution of enhancement factors for the optimizations in Fig. 2 (main text) as function of the accumulated nutation angle θ^{DNP}. The aim of this analysis is to explore the ability of *in situ* Bayesian optimization to cope with the challenge of MW inhomogeneity which should favor good sequences with small accumulated nutation angles. Indeed, the best sequences in Fig. S4A (Monte Carlo) and Fig. S4B centers around resonances with k_I values of 2 (ZQ and DQ) providing the highest transfer. Less efficient, but still highly populated, sequences are also found around the k_I=0 band. In particular the former illustrates (as also clear from the trajectories in Fig. 2, main text) that unconstrained

Bayesian finds sequences coping with MW inhomogeneity. This is not effectively captured by the Monte Carlo optimizations. In the constrained optimizations, some flexibility around the resonance is observed.

- Fig. S5: Analysis of the distribution of enhancement factors for the optimizations in Fig. 2 (main text) as function of the number of repetitions of the DNP transfer element. The number of elements was allowed to vary from 1 to 30 in the optimizations.

- Fig. S8: Analysis of *in situ* constrained cRW Bayesian optimizations on trityl OX063 with glycerol-d_5 using different number of pulses in the 120 ns DNP transfer element.

- Fig. S9: Comparison between simulated and experimental performance of the pulse sequences tested in Figs. 2A-C,E. Each of the four panels use different scaling of the simulated contact time.

- Fig. S10: Simulation of the polarization transfer in a 2-spin model for the 24-pulse BayesOpt sequence found in Fig. 2. The transfer is given for different scalings of the MW pulse amplitude, for different electron spin offsets and for four different contact times.

- Fig. S11: Experimental analysis of pulse sequence elements (excitation pulse and transfer element) for DNP on 2 mM 4-Oxo-TEMPO in DMSO-d_6. The analysis explores *in situ* Bayesian optimizations for different pulse sequences, leading to the setup used in Fig. 4.

- Fig. S12: Experimental optimization of contact time and MW amplitude for NOVEL-type constant amplitude spin-lock DNP for 2 mM 4-Oxo-TEMPO in DMSO-d_6 relating to the experiments in Fig. 4. It is noted that highest transfer is obtained at higher MW amplitude than used for NOVEL (14.8 MHz) while good transfer is also obtained at the NOVEL condition.

- Fig. S17: Analysis of the predicted feedback for the optimization of the initial flip pulse as shown in Figure 3A. The model error is found by comparing the predicted feedback to experiment feedback.

- Fig. S18: Analysis of the predicted feedback for the optimization of the initial flip pulse as shown in Fig. 4B. The model error is found by comparing the predicted feedback to experiment feedback.

- Fig. S19: Experimental offset profile recorded for trityl OX063 in glycerol-d_5 for the Bayesian optimized 4-pulse excitation pulse followed by a cRW-OPT1 DNP transfer element (repeated 5) times along with comparison of ^{1}H NMR spectra recorded with and without DNP with the optimal 4-pulse-excitation-cRW-OPT1 DNP sequence.

- Fig. S20: Experimental offset profiles recorded for trityl OX063 in glycerol-d_5 for the Bayesian optimized 4-pulse excitation pulse sequence combined with cRW-OPT1 (5 repetions) DNP transfer pulse sequence in Fig. 3B relative to NOVEL, PLATO, and cRW-OPT1 DNP transfers (to compare with Fig. 3F using glycerol-d_8 in the solvent).

4 Optimized pulse sequences

The optimal pulse sequences obtained by *in situ* Bayesian optimization is represented by DNP transfer sequences for trityl and TEMPO radicals and excitation pulse for trityl as listed below with reference to figures in the main text.

4.a Optimized DNP transfer pulse sequences

The DNP transfer pulse sequences resulting from *in situ* Bayesian optimization is given in Tables S6 and S11 as specified below

- Table S6: Pulse sequence resulting from constrained Bayesian optimization of a 24-pulse DNP transfer element (t_m = 120 ns) obtained by optimization on trityl OX063 in glycerol-d_5 as represented in Fig. 2E (main text).

- Table S11: Pulse sequence resulting from constrained Bayesian optimization of a 8-pulse DNP transfer element (t_m = 10 μs; each pulse 1250 ns) obtained by optimization on a 5 mM sample of 4-Oxo-TEMPO in DMSO-d_6 as represented in Fig. 4B (main text).

4.b Optimized excitation pulses

- Table S7: Optimal 4-pulse excitation pulse combined with cRW-OPT1 DNP transfer (repeated 5 times) optimized for trityl OX063 in glycerol-d_5 from Fig. 3A.

5 Parameters for optimizations

The optimizations presented in this work, both *in situ* (experimentally) and *in silico* (numerically) involved setting of optimization parameters which are presented in a series of tables with description of their specific connection to the figures presented in main text and in Supplementary Figures.

5.a *in situ* optimizations

Parameters used for *in situ* optimizations on trityl OX063 in glycerol-d_5 and 2 mM 4-Oxo-TEMPO in DMSO-d_6. In the list below we specify which sample (Trityl or TEMPO) and figure the parameters corresponds to with more details given in the specific table.

- Table S1: Trityl, Fig. 2A-C
- Table S2: Trityl, Fig. 2E
- Table S5: Trityl, Fig. S8
- Table S8: Trityl, Fig. 3A
- Table S9: TEMPO, Fig. S11A,B
- Table S10: TEMPO, Fig. S11C,D
- Table S12: TEMPO, Fig. 4

5.b *in silico* optimizations

The numerical optimization used spin-pair parameters matching trityl with the tables presenting parameters used in optimizations relating to the marked figure.

- Table S3: Fig. S6
- Table S4: Fig. S7
- Table S13: Fig. S16(Aa-Ca)
- Table S14: Fig. S16(Ab-Cb)
- Table S15: Fig. S16(Ac-Cc)
- Table S16: Fig. S16(Ad-Ad)

Supplementary Figures

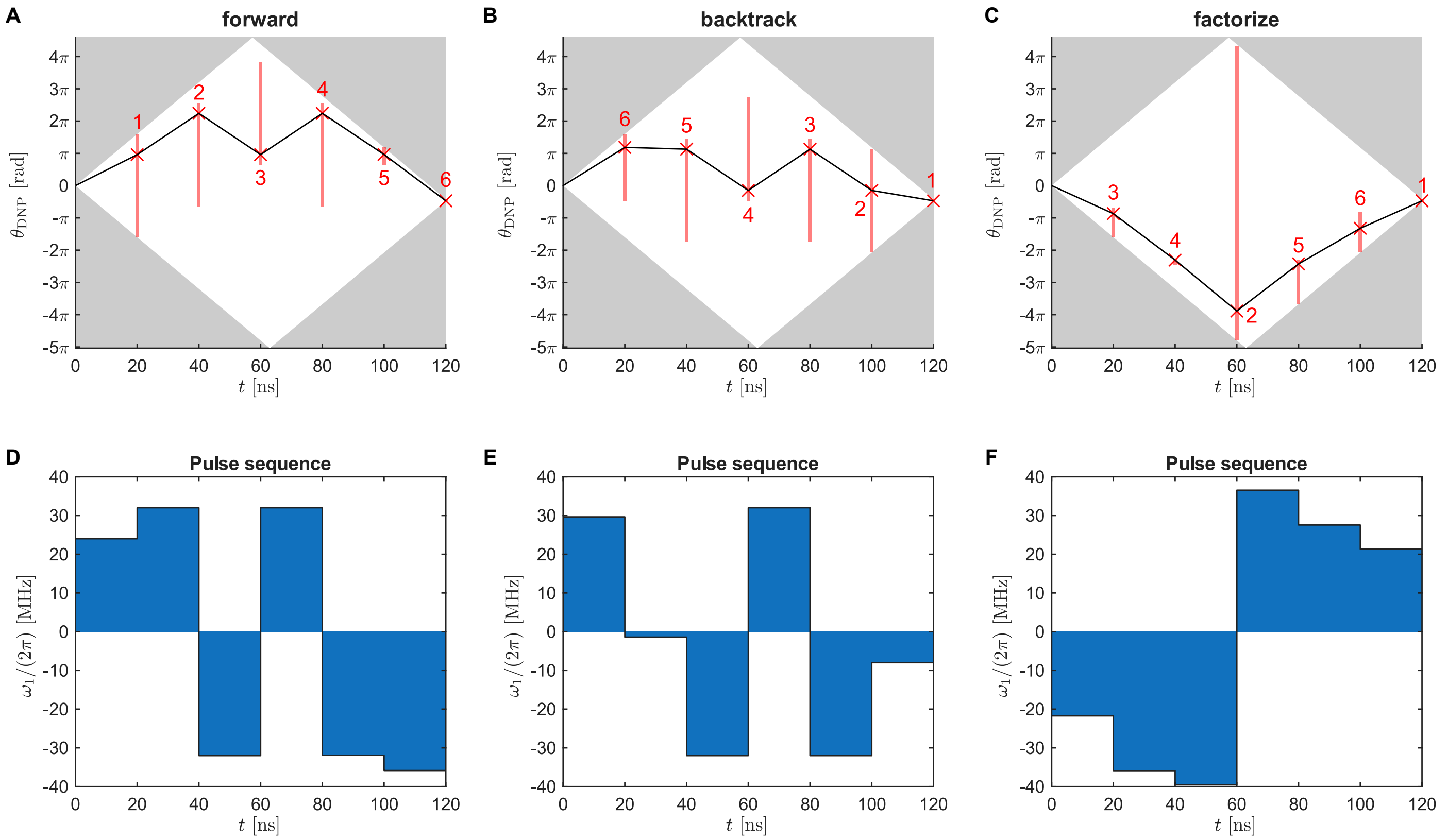

Figure S1: **Construction of pulse sequences for different cRW orders.**

(A-C) Three different ways of constructing the cRW. The red crosses show the resulting accumulated nutation angles. The numbers indicate the order in which the angles were fixed, and the vertical bars show the available range. All three cRW's used the input vector $x = [0.8, 0.9, 0.1, 0.9, 0.6, 0.1]$, 6 pulses each of 20 ns duration, 14.8 MHz proton Larmor frequency, 40 MHz maximal nutation frequency, and the DQ, $k_I = 2$ DNP resonance with -5 to 5 degrees DNP offset. (A) Angles are fixed in chronological order. (B) Angles are fixed in reverse order. (C) Angles that are most distant in time to any currently fixed angles are fixed first. (D-F) Corresponding pulse sequence for each of the cRW's given as $\pm x$-phase amplitudes.

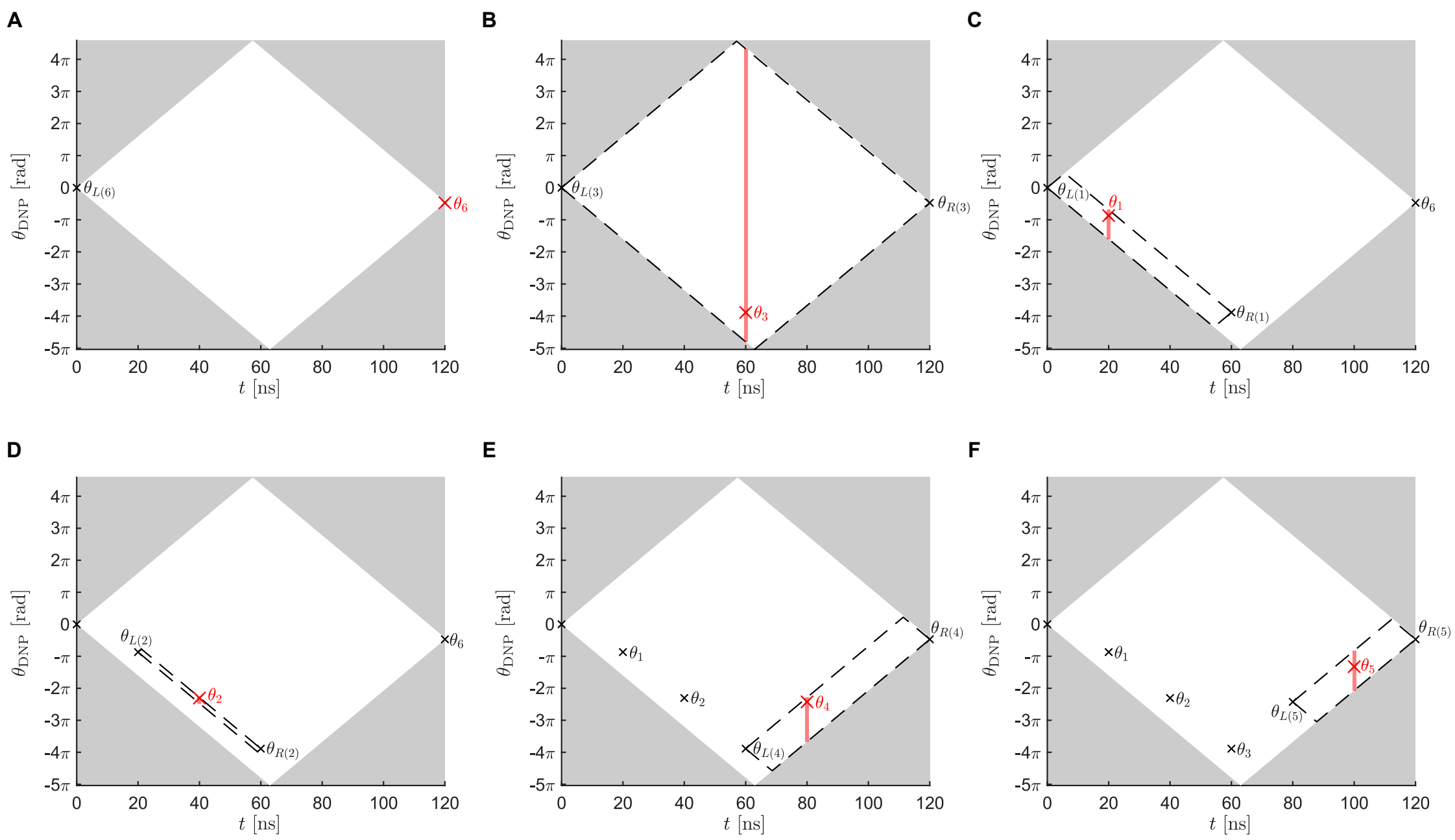

Figure S2: **Detailed example of cRW pulse sequence construction.**

A walkthrough of how to construct the cRW seen in Fig. S1C using the input vector $x = [0.8, 0.9, 0.1, 0.9, 0.6, 0.1]$ and the "factorize" order where most distant angles are fixed first. In all panels the slope of the dashed line corresponds to maximal positive or negative nutation frequency of 40 MHz. (A) Only the first angle is fixed and therefore θ_6 is furthest away in time. Since it only has a fixed angle to the left $\theta_{L(6)} = \theta_0$, it uses Eqs. (S8) - (S9) to determine the allowed range (only 10° wide and too small to see on figure). The angle θ_6 is placed 10% up this range according to $x_6 = 0.1$. (B) The first and last angles are fixed so θ_3 is furthest away. The nearest fixed angles are $\theta_{L(3)} = \theta_0$ and $\theta_{R(3)} = \theta_6$ and Eqs. (S6) - (S7) determine the allowed range (red line). The angle θ_3 is placed 10% up this line according to $x_3 = 0.1$. (C) All undetermined angles are 20 ns from a fixed angle so in this case the first of them θ_1 is chosen. The nearest fixed angles are $\theta_{L(1)} = \theta_0$ and $\theta_{R(1)} = \theta_3$ and Eqs. (S6) - (S7) determine the allowed range (red line). The angle θ_1 is placed 80% up this line according to $x_1 = 0.8$. (D) Next θ_2 is chosen. The nearest fixed angles are $\theta_{L(2)} = \theta_1$ and $\theta_{R(2)} = \theta_3$ and Eqs. (S6) - (S7) determine the allowed range (red line, barely visible). The angle θ_2 is placed 90% up this line according to $x_2 = 0.9$. (E) Next θ_4 is chosen. The nearest fixed angles are $\theta_{L(4)} = \theta_3$ and $\theta_{R(4)} = \theta_6$ and Eq. (S6) - (S7) determine the allowed range (red line). The angle θ_4 is placed 90% up this line according to $x_4 = 0.9$. (F) The last angle θ_5 is chosen. The nearest fixed angles are $\theta_{L(5)} = \theta_4$ and $\theta_{R(5)} = \theta_6$ and Eqs. (S6) - (S7) determine the allowed range (red line). The angle θ_5 is placed 60% up this line according to $x_5 = 0.6$.

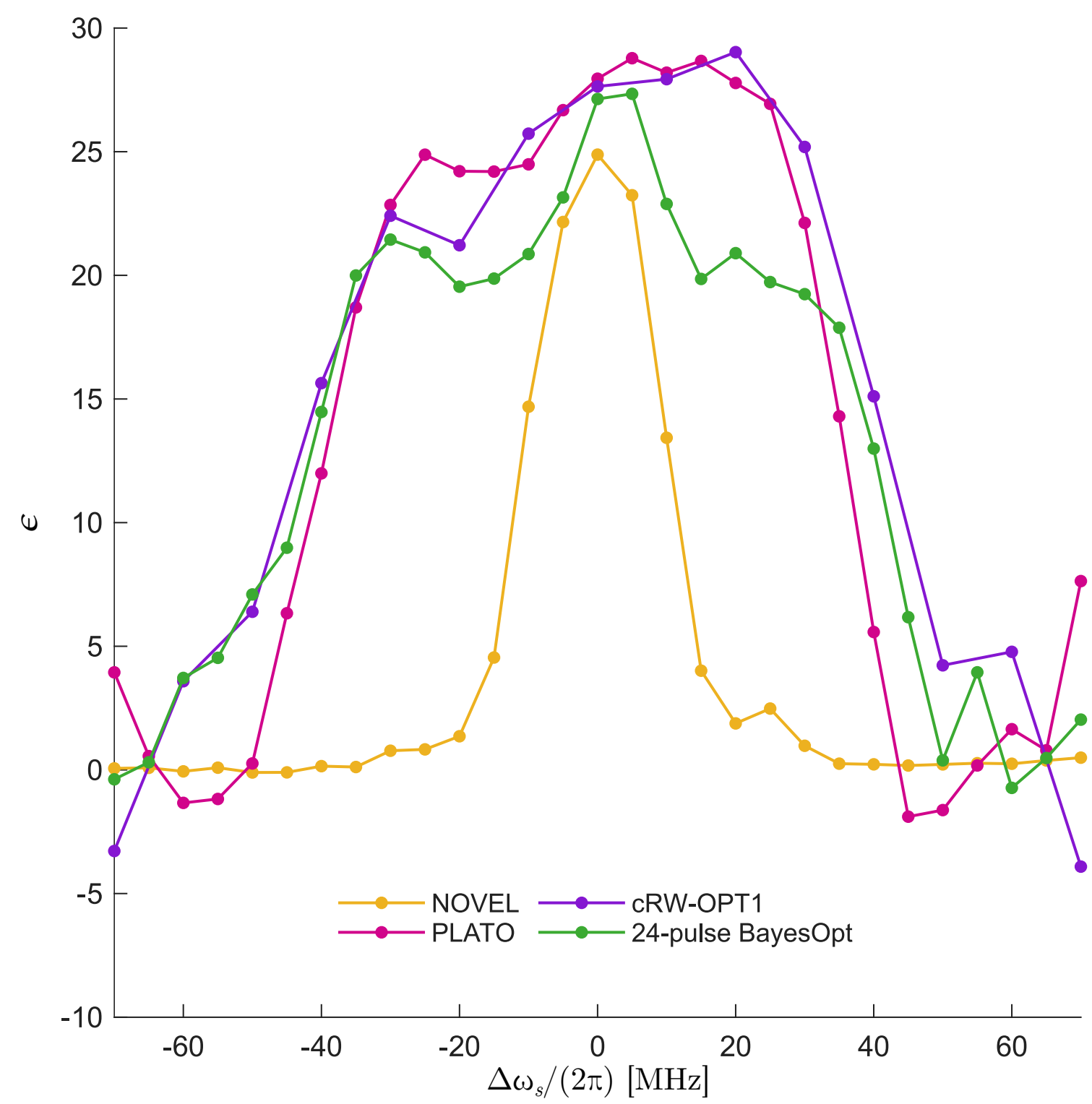

Figure S3: **Offset profiles for different DNP transfer elements using trityl in glycerol-d_5.**

Experimental *in situ* polarization enhancements as a function of electron-spin offset using the same pulse sequences employed in Fig. 3F but on a sample of trityl in glycerol-d_5 and with 5 s buildup time.

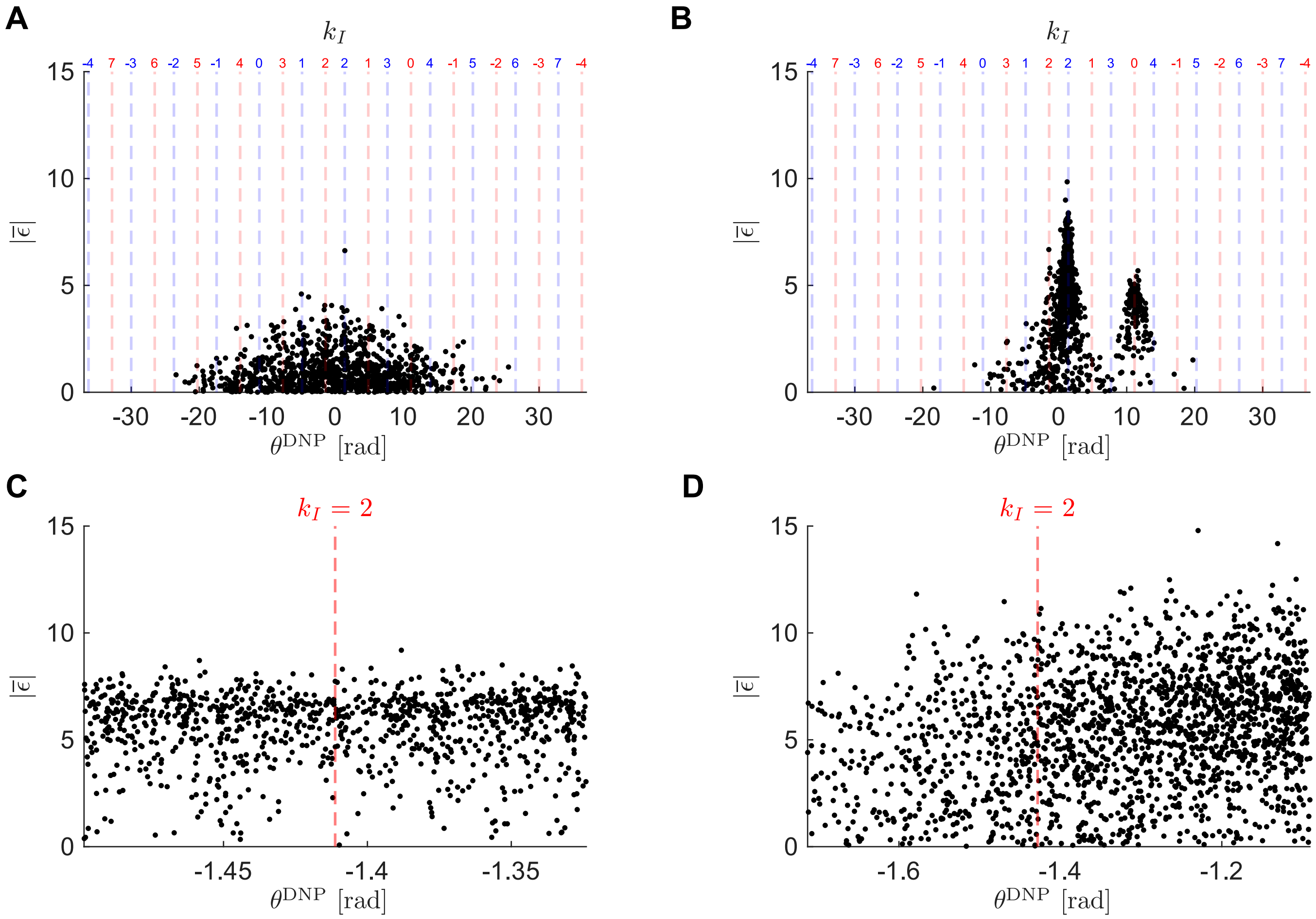

Figure S4: **DNP resonance analysis.**

Enhancement factors for the *in situ* optimized DNP sequences in Figs. 2 Aa-Ca,e, as a function of the accumulated nutation angle θ^{DNP} of the transfer element for the given sequence. In each panel, the nutation angle axis spans the allowed range of effective angles which in the constrained optimizations centers around the selected resonance. The optimizations were: (A) Monte Carlo, (B) Bayesian, (C) constrained Bayesian, and (D) constrained Bayesian with refined options (see Table S2).

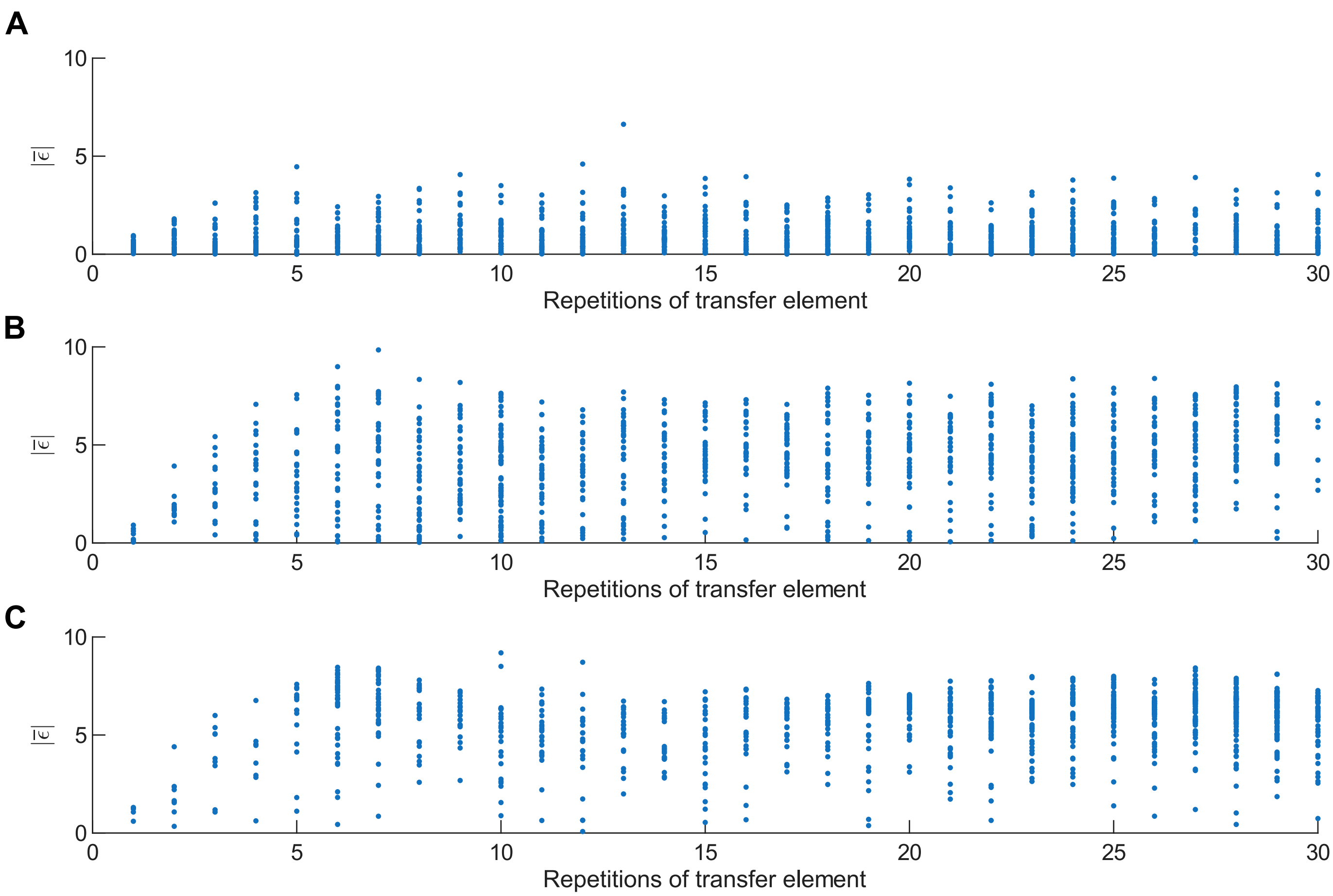

Figure S5: **Analysis of transfer element repetitions.**

Enhancement factors for the DNP transfer element pulse sequences found in Figs. 2 Aa-Ca as a function of the number of times the DNP transfer element was repeated for the given sequence. The optimizations were, as specified in Fig. 2: (A) Monte Carlo, (B) Bayesian, and (C) constrained Bayesian.

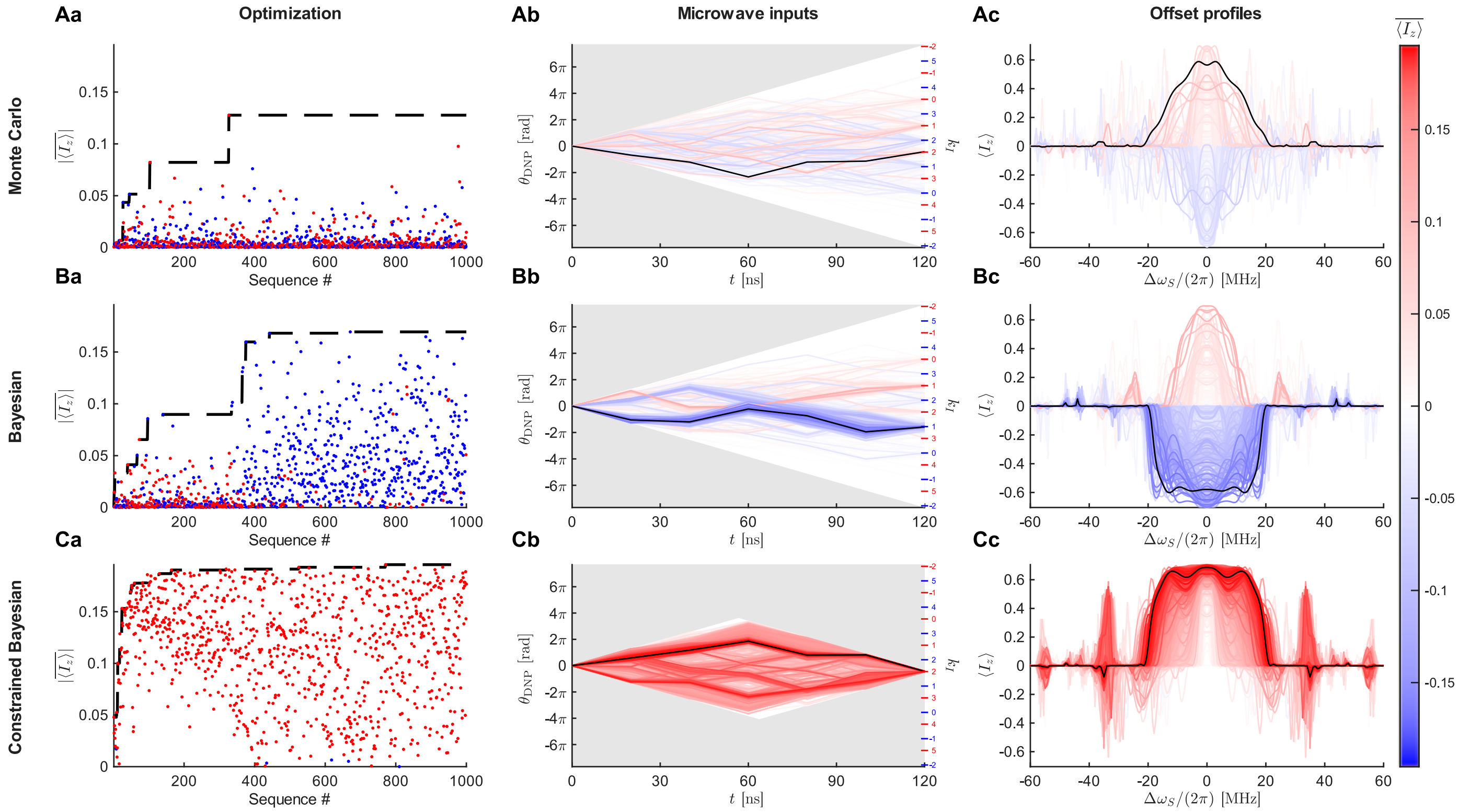

Figure S6: **Performance of *in silico* Bayesian optimization.**

Comparison of three different optimization methods by *in silico* optimization: (A) Monte Carlo, (B) Bayesian, and (C) constrained Bayesian. The layout is similar to Fig. 2A-C). (Aa-Ca) Absolute average polarization $|\overline{\langle I_z \rangle}|$ of the offset profile for each sequence, with red and blue dots representing match to DQ and ZQ resonances. (Ab-Cb) Accumulated nutation angle during transfer for each of the evaluated sequences. Colored numbers reflects k_I values for DQ (red) and ZQ (blue). (Ac-Cc) Offset profiles of the DNP polarization with the electron spin offset ranging from -60 to 60 MHz. In columns 2) and 3) the color is weighted by the absolute average enhancement. The best sequence in each optimization is marked in black. All results represent simulations for an electron-proton spin-pair with a distance of 4.5 Å, an initial density matrix S_x, a nuclear Larmor frequency 14.8 MHz, and a maximal MW nutation frequency of 32 MHz. A powder average was made using 20 polar angles between 0 and π radians. Each pulse sequence element consisted of 6 $\pm x$-phase pulses each of duration of 20 ns, which were repeated until the most efficient transfer was reached. The acquisition function for the Bayesian optimization in (B) and (C) was "expected-improvement-plus". For the constraint in (C) the cRW order "forward" was used with a DNP offset between $-5°$ and $5°$. Parameters for the optimizations are listed in Table S3.

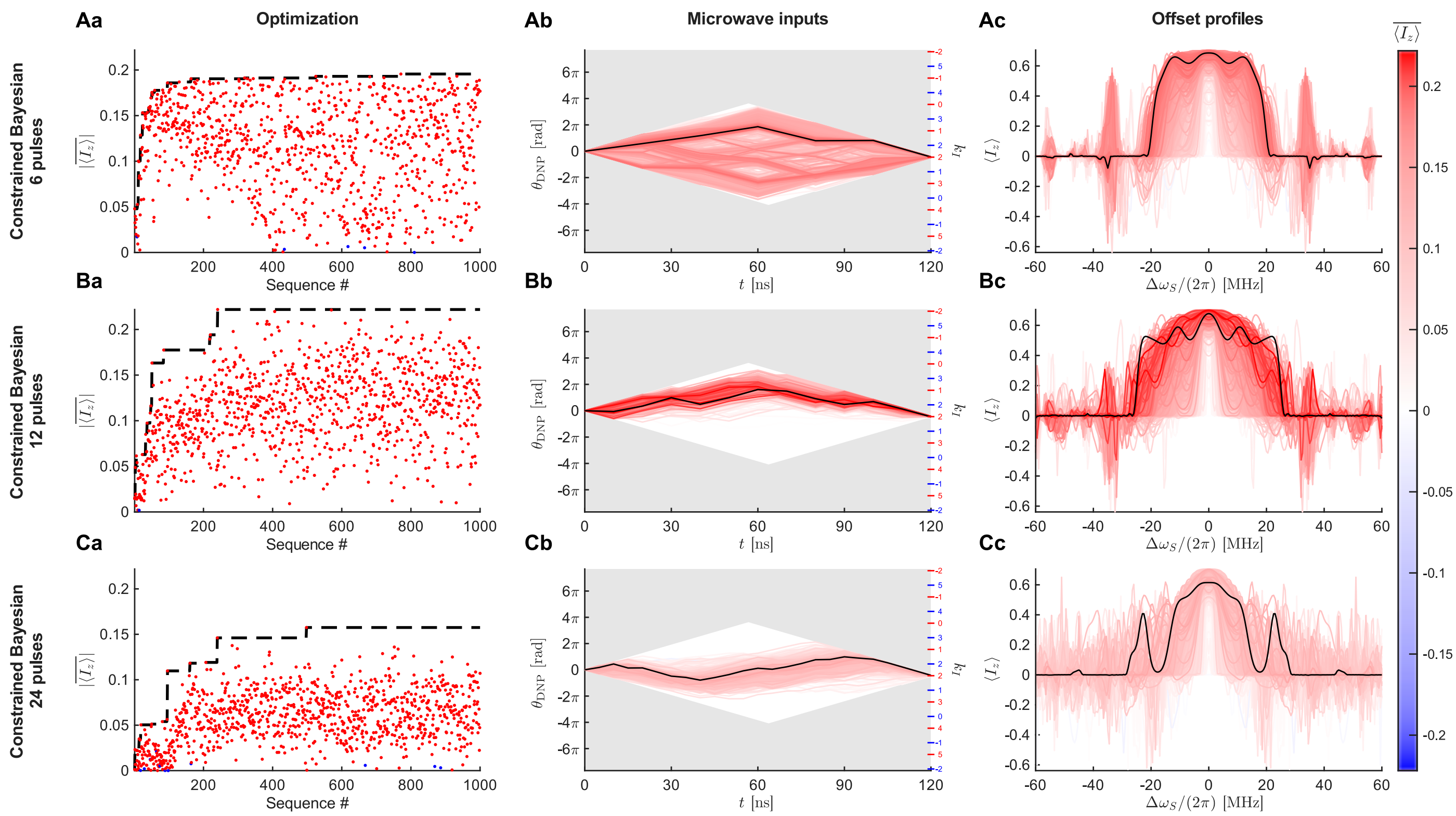

Figure S7: ***In silico* performance of constrained Bayesian optimization for different number of pulses.**

The results from three *in silico* constrained Bayesian optimizations using different numbers of pulses: (A) 6 pulses, (B) 12 pulses, and (C) 24 pulses. In all cases, the modulation time is fixed to 120 ns. Otherwise, the layout and settings are the same as in Fig. S6C. Parameters for the optimizations are listed in Table S4.

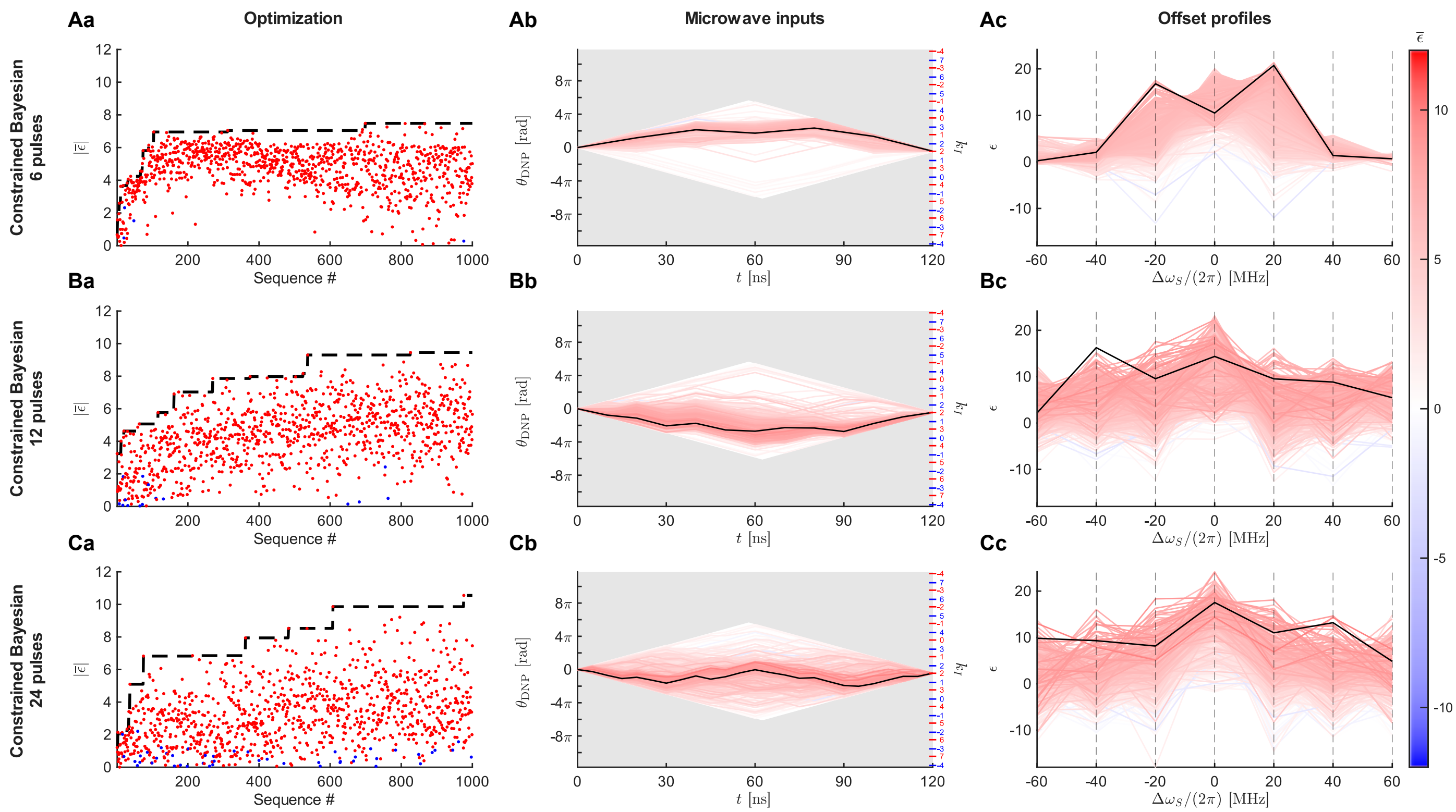

Figure S8: ***In situ* performance of constrained Bayesian optimization for different number of pulses.** Experimental *in situ* constrained Bayesian optimizations of the DNP transfer for trityl OX063 with glycerol-d_5 using DNP transfer elements with different numbers of pulses: (A) 6 pulses, (B) 12 pulses, and (C) 24 pulses. In all three cases, the modulation time is fixed at 120 ns. Here the constraint followed the "factorize" cRW order, but otherwise the layout and settings are the same as in Fig. 2C. Parameters for the optimizations are listed in Table S5.

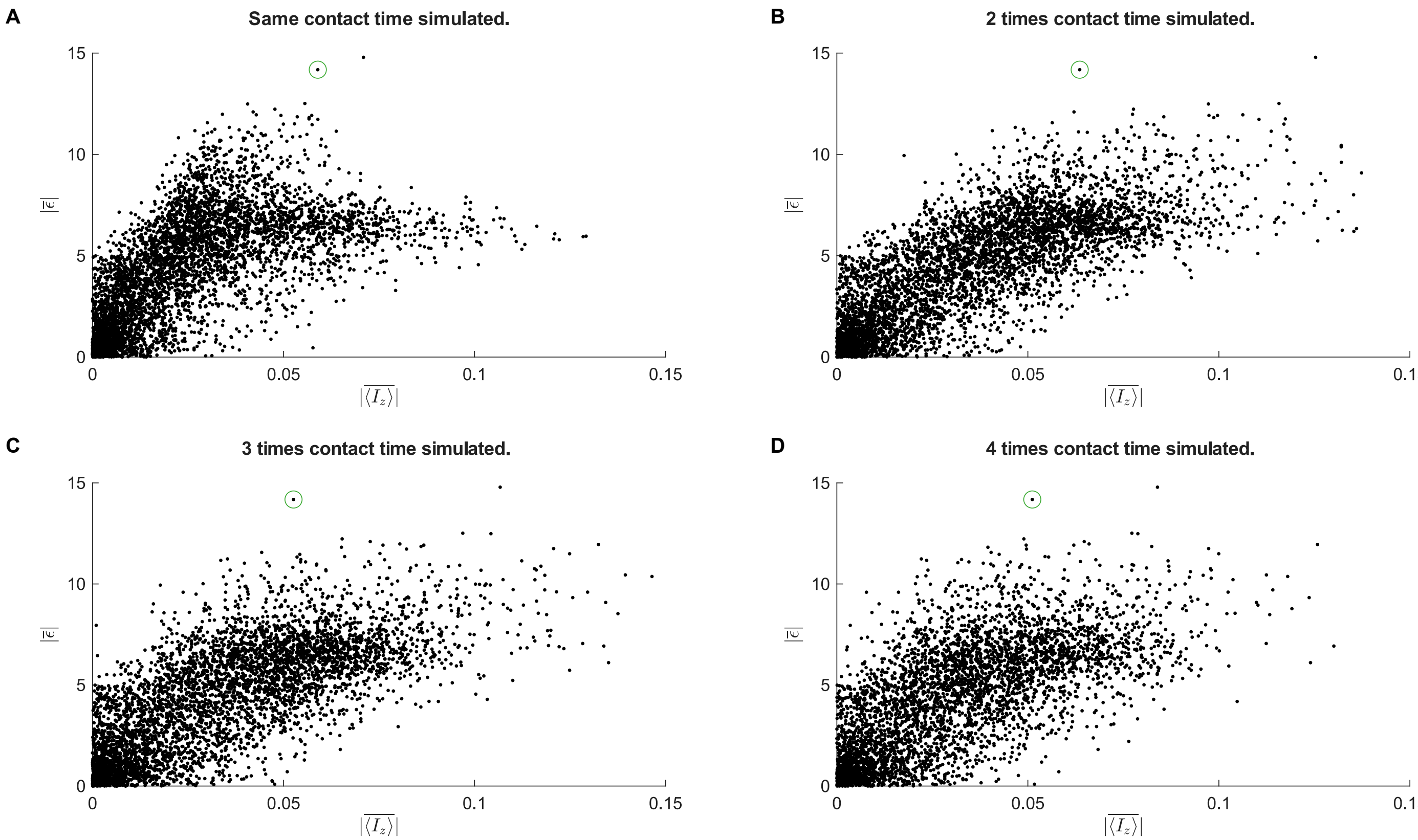

Figure S9: ***In situ* and *in silico* comparison of transfer element optimization for trityl DNP.**

Comparison of *in silico* and *in situ* performance for the DNP pulse sequences derived in Figs. 2A-C,E. The average of the *in situ* enhancements were taken over the electron spin offsets at $\Delta\omega_S/(2\pi)$ = -60, -40, -20, 0, 20, 40, and 60 MHz. For each of the experimental offsets, a range of 5 offsets were simulated with a difference of $\delta\omega_S/(2\pi)$ = -4, -1.5, 0, 1.5, and 4 MHz to the experimental offset. The polarization from these 5 offsets were weighted by 0.0947, 0.2621, 0.3145, 0.2599, and 0.0688 corresponding to the intensity profile of the trityl EPR line. The offset average of the *in silico* polarizations was then found as a weighted average over the resulting 35 offsets. The point corresponding to the 24-pulse BayesOpt sequence analyzed in Fig. 2F is highlighted by a green circle. Each of the four panels use different scaling of the simulated contact time: (A) Same contact time, (B) double contact time, (C) triple contact time, and (D) quadruple contact time.

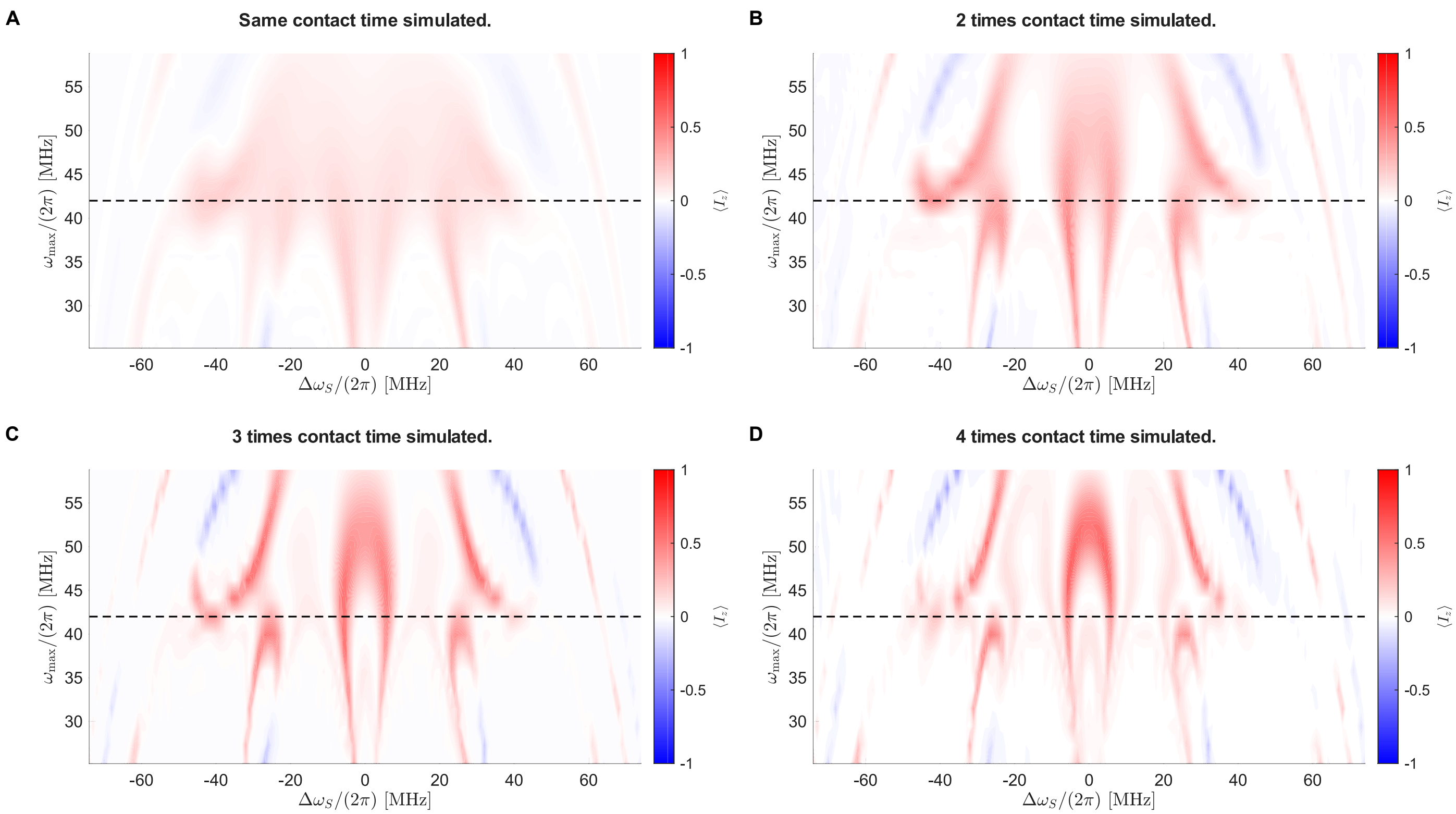

Figure S10: **Electron offset and MW inhomogeneity analysis for the 24-pulse BayesOpt sequence.** Simulated transfer efficiency as function of electron-spin offset and variations in the maximum MW amplitude for the 24-pulse BayesOpt sequence analyzed in Fig. 2F. The sequence as given in Table S6 uses $\omega_{\max}/(2\pi) = 42$ MHz which is indicated by the dashed line. Moving along the vertical axis the pulse amplitudes in the simulation are scaled accordingly. The four panels show the polarization at different contact times: (A) Same as experiment (720 ns), (B) double contact time (1440 ns), (C) triple contact time (2160 ns), and (D) quadruple contact time (2880 ns).

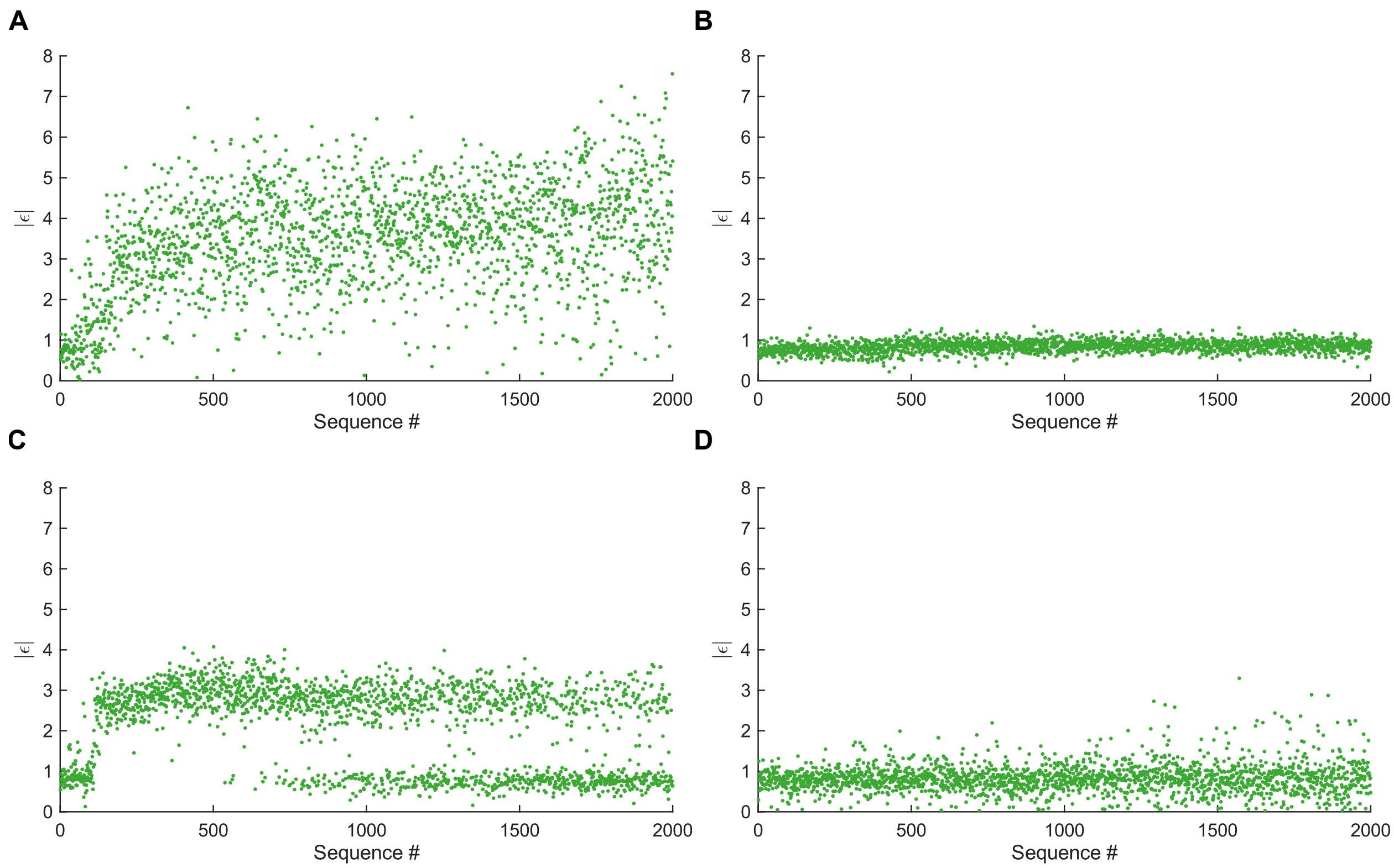

Figure S11: **Investigation of different pulse sequence designs for DNP using TEMPO.**

in situ (experimental) Bayesian optimization of DNP enhancement with 2 mM 4-Oxo TEMPO using different pulse sequence designs. (A) 4-pulse excitation and 4-pulse DNP transfer element, both with arbitrary amplitude and phase. Excitation pulses had the same duration in the range of 0 - 5 ns. The DNP transfer element was allowed to be repeated 1 - 50 times, and the common duration of the transfer pulses was set to best match a total contact time of 10 μs. (B) 20-pulse DNP transfer element with arbitrary amplitude and phase and no preceding excitation (i.e., aiming at longitudinal polarization transfer DNP (*29*)). The transfer element was repeated 1 - 100 times, and the common duration of the transfer pulses was set to best match a contact time of 10 μs. (C) Fixed 52 MHz amplitude y-pulse excitation of duration of 5.5 ns combined with a 30-pulse DNP transfer element with arbitrary amplitude and $\pm x$ phase. The transfer element was repeated 1 - 100 times, and the common duration of the transfer pulses was set to best match a contact time of 10 μs. (D) 4-pulse excitation and 10-pulse DNP transfer element with arbitrary amplitude and phase. Excitation pulses had the same duration in the range 0 - 5 ns. The transfer element was repeated 1 - 100 times, and the common duration of the transfer pulses was set to best match a contact time between 0 - 10 μs. Parameters for the optimizations are listed in Tables S9 and S10.

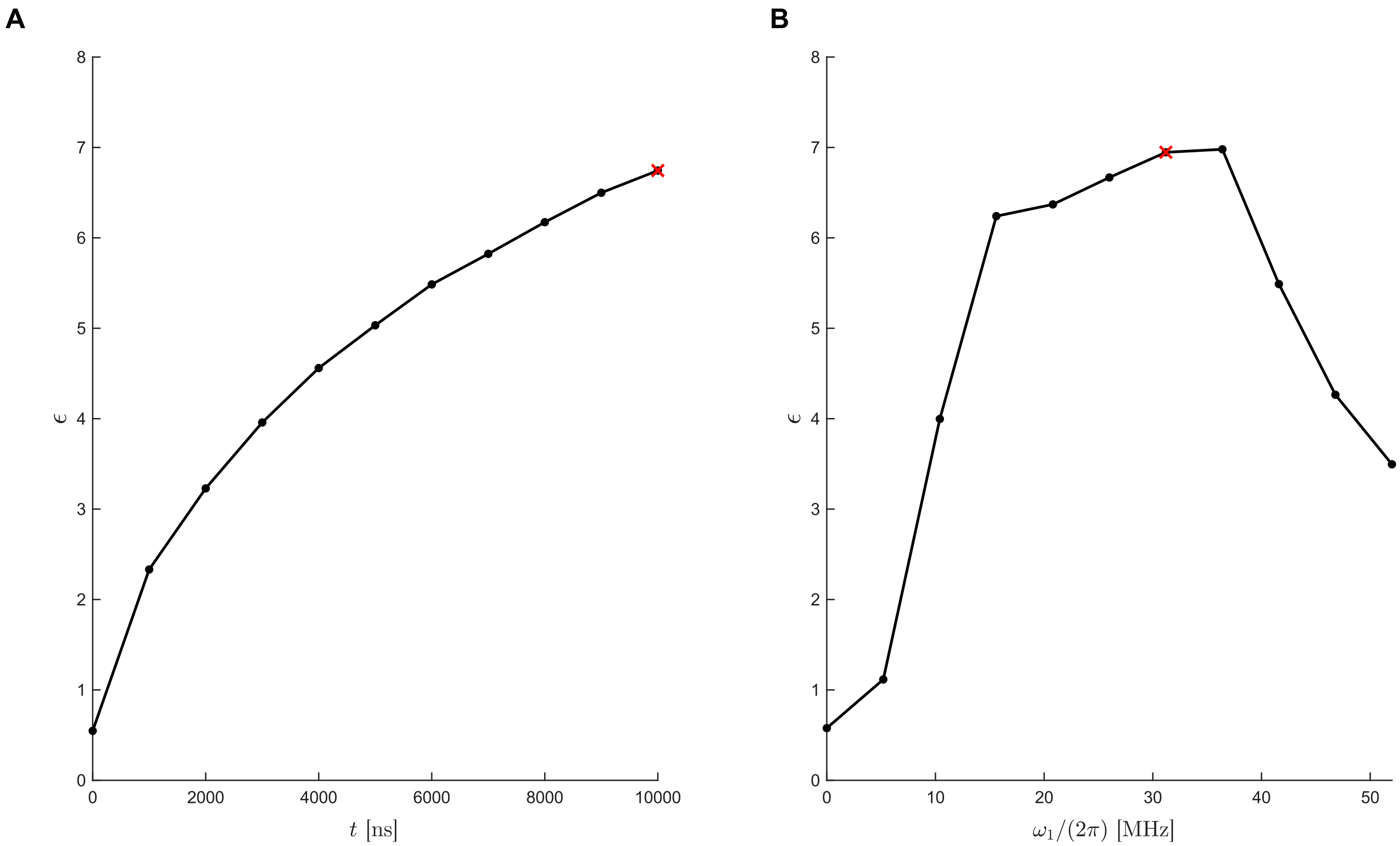

Figure S12: **Lock-pulse optimization for DNP using TEMPO.**

in situ (experimental) optimization of constant amplitude transfer using 4-Oxo-TEMPO with the red crosses marking the values used in Fig. 4: (A) Contact time. (B) MW nutation frequency.

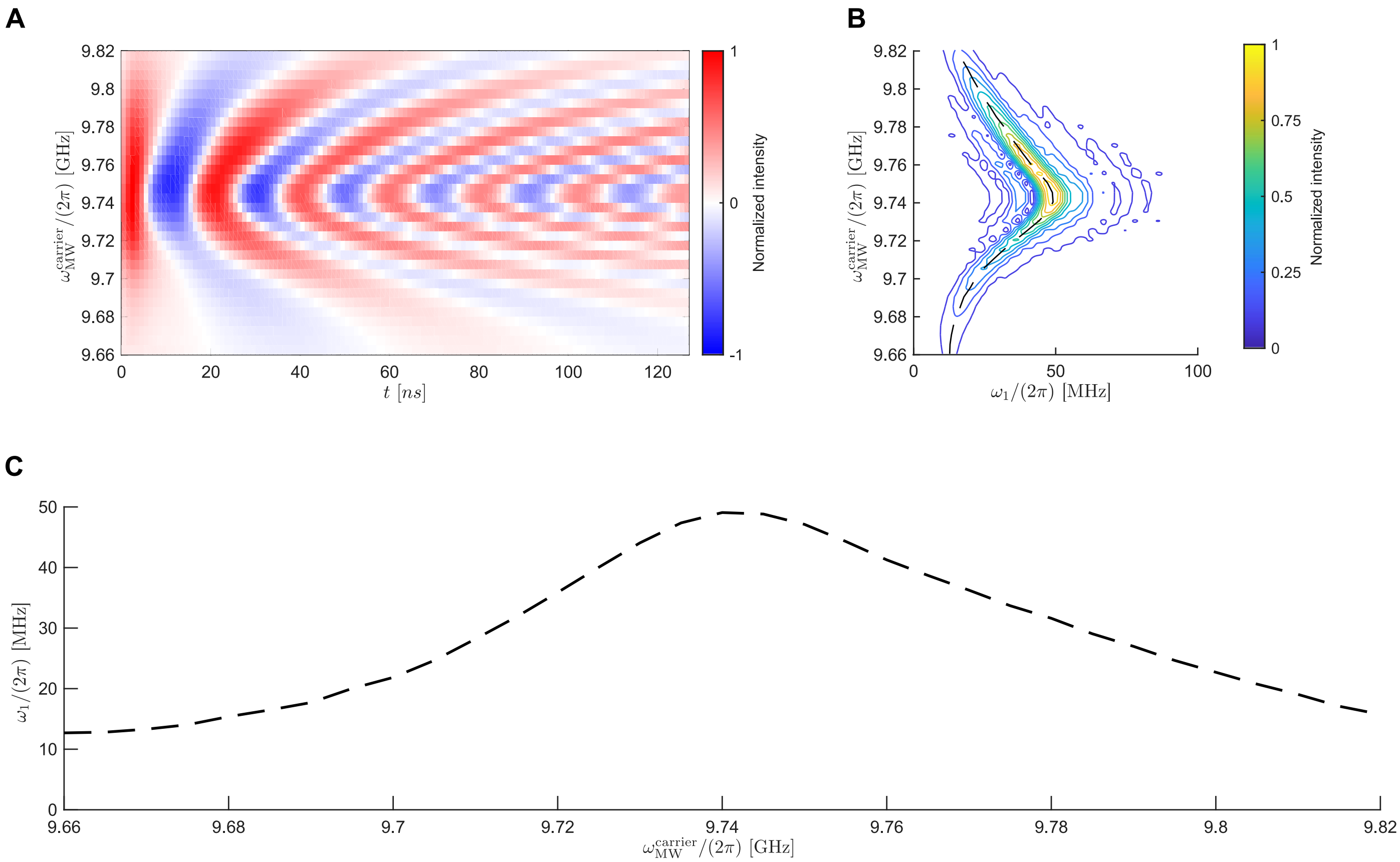

Figure S13: **MW resonator profile.**

(A) Nutation curves for different MW carrier frequencies. The experiments used a 100% amplitude pulse, in a digital scale, with the duration shown on the horizontal axis. The echo detection occured 10 μs after the start of the pulse, with pulse durations of 16 ns for the $\pi/2$-pulse and 32 ns for the π-pulse and a delay of 384 ns between them. The vertical axis indicates the carrier frequency of each nutation curve. To stay on resonance, the magnetic field followed the carrier $B_0 = \omega_{MW}^{\text{carrier}}/\gamma_{\text{exp}}$ with a gyromagnetic ratio of $\gamma_{\text{exp}} = 2.849$ MHz/G. (B) Fourier transform of the nutation curves in (A)). The dashed line indicates the maximal intensity frequency for each carrier, which defines the resonator profile shown in (C).

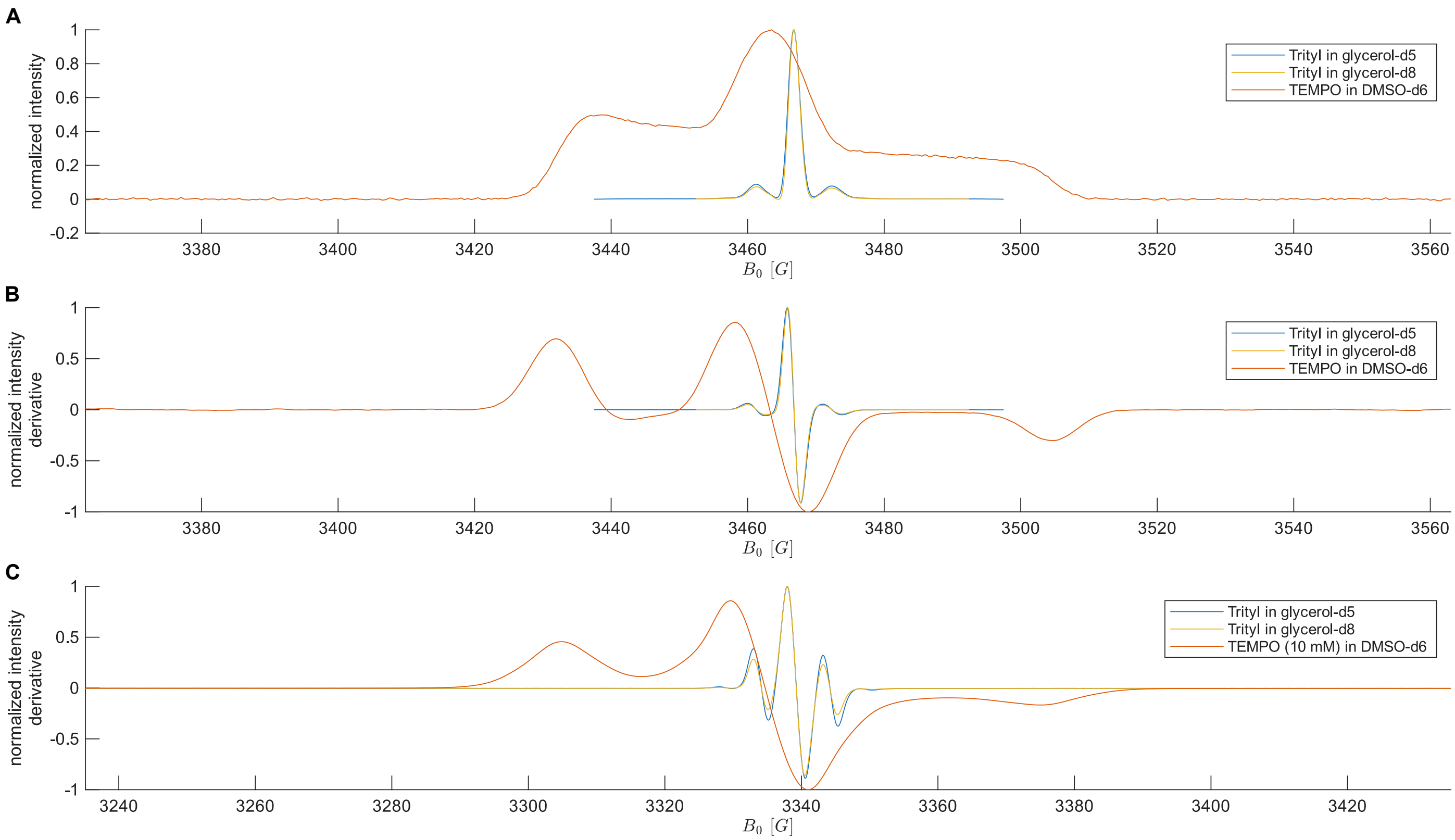

Figure S14: **EPR profiles.**

Normalized EPR profiles for trityl OX063 in glycerol-d_5 and glycerol-d_8 and 4-Oxo-TEMPO in DMSO-d_6 obtained from field sweeps. (A) EPR-echo intensities measured at different magnetic fields with the MW carrier at 9.72 GHz. (B) Intensity derivatives calculated from local linear fits of the experiment data using a window of 2 G (21 points) for trityl in glycerol-d_5, 2 G (41 points) for trityl in glycerol-d_8 and 10 G (21 points) for TEMPO in DMSO-d_6. (C) Intensity derivatives measured directly using a CW EPR spectrometer with the MW carrier at 9.36 MHz. Notice the higher concentration of 10 mM TEMPO and that the limits of the B_0-axis have changed.

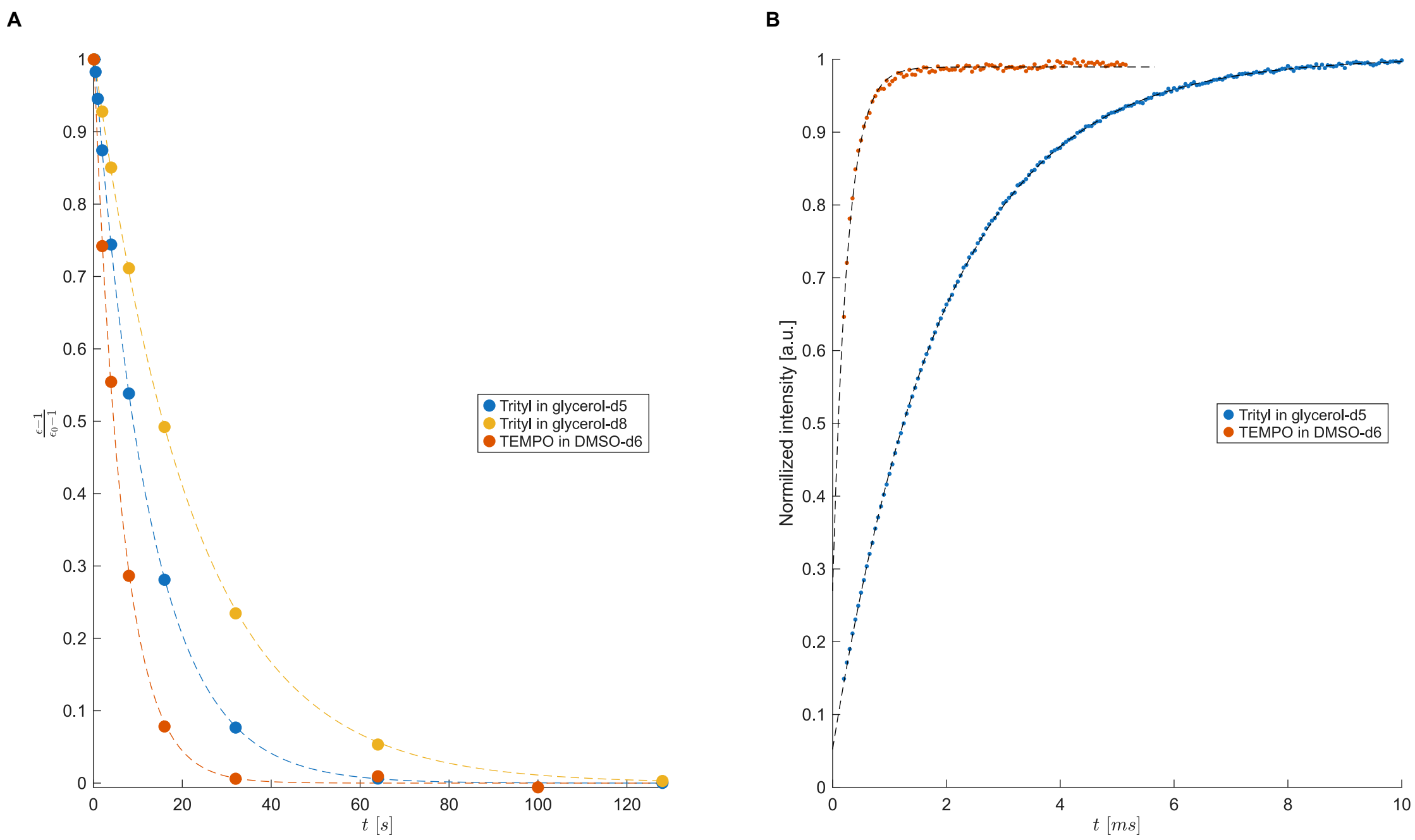

Figure S15: **Relaxation properties.**

(A) Proton T_1 relaxation behavior after NOVEL DNP for the three different samples investigated in this study. The horizontal axis indicates the delay between DNP and the NMR solid echo and the vertical axis indicates the normalized enhancements after subtraction of the reference signal. Each sample uses a different normalization implying that the normalized enhancement is not comparable in-between different samples. The dashed lines indicate a fit to an exponential model Ae^{-t/T_1} with nuclear T_1 values of 12.4 s for trityl in glycerol-d_5, 22 s for trityl in glycerol-d_8 and 6 s for TEMPO in DMSO-d_6. For trityl in glycerol-d_5, the experiment used a 5.5 ns flip pulse of 45 MHz, and a 2 μs lock pulse of 19 MHz with a buildup of 64 s. For trityl in glycerol-d_8, the experiment used a 5.5 ns flip pulse of 40 MHz and a 8 μs lock pulse of 14.4 MHz with a buildup of 128 s. For TEMPO in DMSO-d_6, the experiment used a 4 ns flip pulse of 52 MHz and a 10 μs lock pulse of 26 MHz with a buildup of 24 s. (B) Measurement of electron recovery time as an indication of electron T_1 for samples of trityl and TEMPO. The vertical axis shows the normalized intensity of the EPR echo with the signal summed over 16 shots and the horizontal axis indicates the repetition time of each shot. The dashed lines indicate a fit to an exponential model $A - Be^{-t/T_1}$ with electron T_1 values of 1.95 ms for trityl in glycerol-d_5 and 0.26 ms for TEMPO in DMSO-d_6. For trityl, the experiment used 15 MHz echo pulses with a duration of 16 ns for the $\pi/2$-pulse and 32 ns for the π-pulse and a delay of 384 ns between them. For TEMPO, the experiment used 17 MHz echo pulses with duration 16 ns for the $\pi/2$-pulse and 32 ns for the π-pulse and a delay of 334 ns between them.

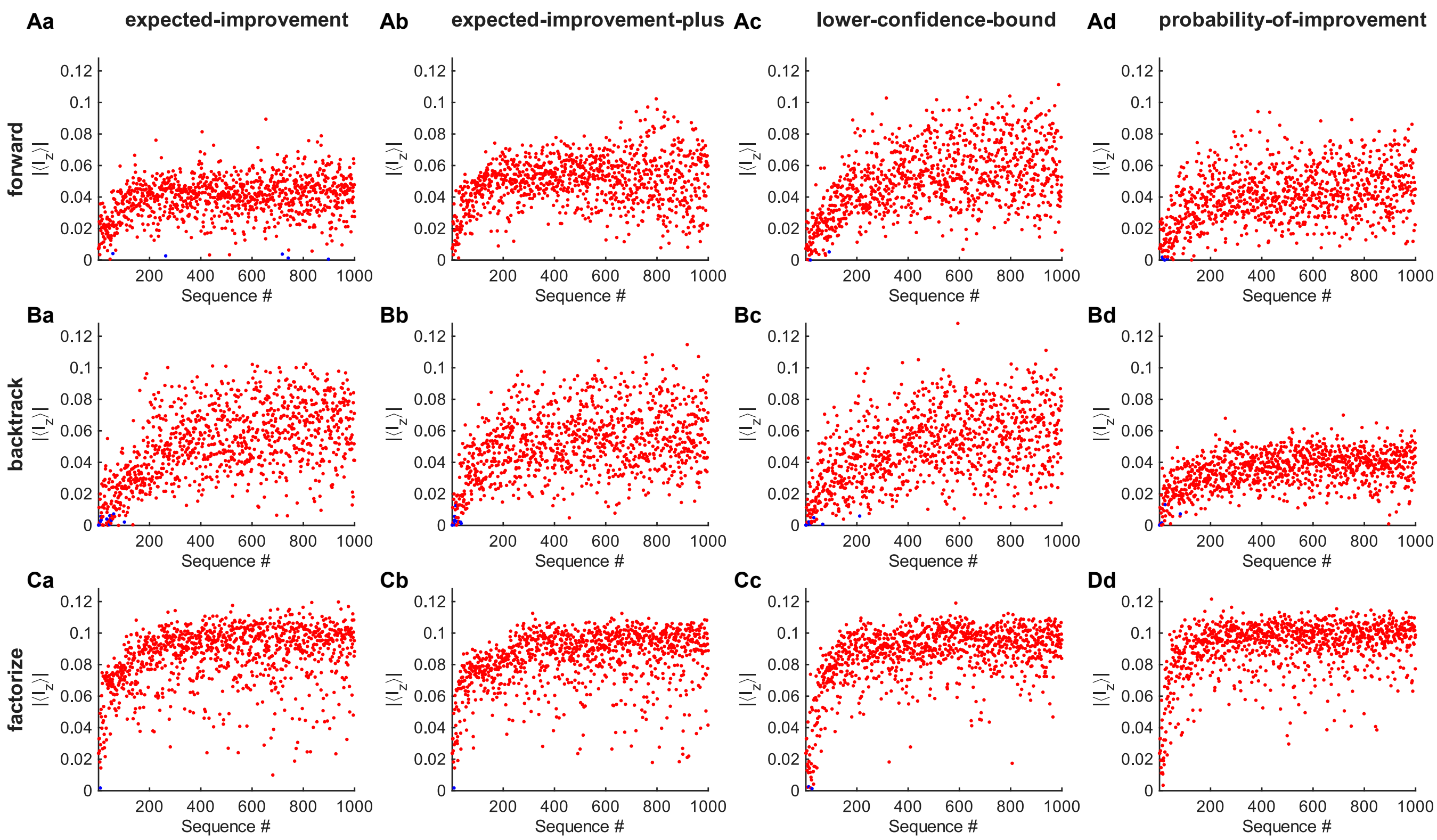

Figure S16: **Choice of acquisition function and cRW order.**

in silico analysis of different acquisition functions and cRW orders for a constrained Bayesian optimization of a 12-pulse DNP transfer element. The rows represent different ways of constructing the cRW: (A) Accumulated nutation angles fixed in chronological order. (B) Accumulated nutation angles fixed in reverse order. (C) Accumulated nutation angles most distant in time to the currently fixed angles, are fixed first. The columns represent different acquisition functions: (a) expected-improvement, (b) expected-improvement-plus, (c) lower-confidence-bound, and (d) probability-of-improvement. Parameters for the optimizations are listed in Tables S13 to S16.

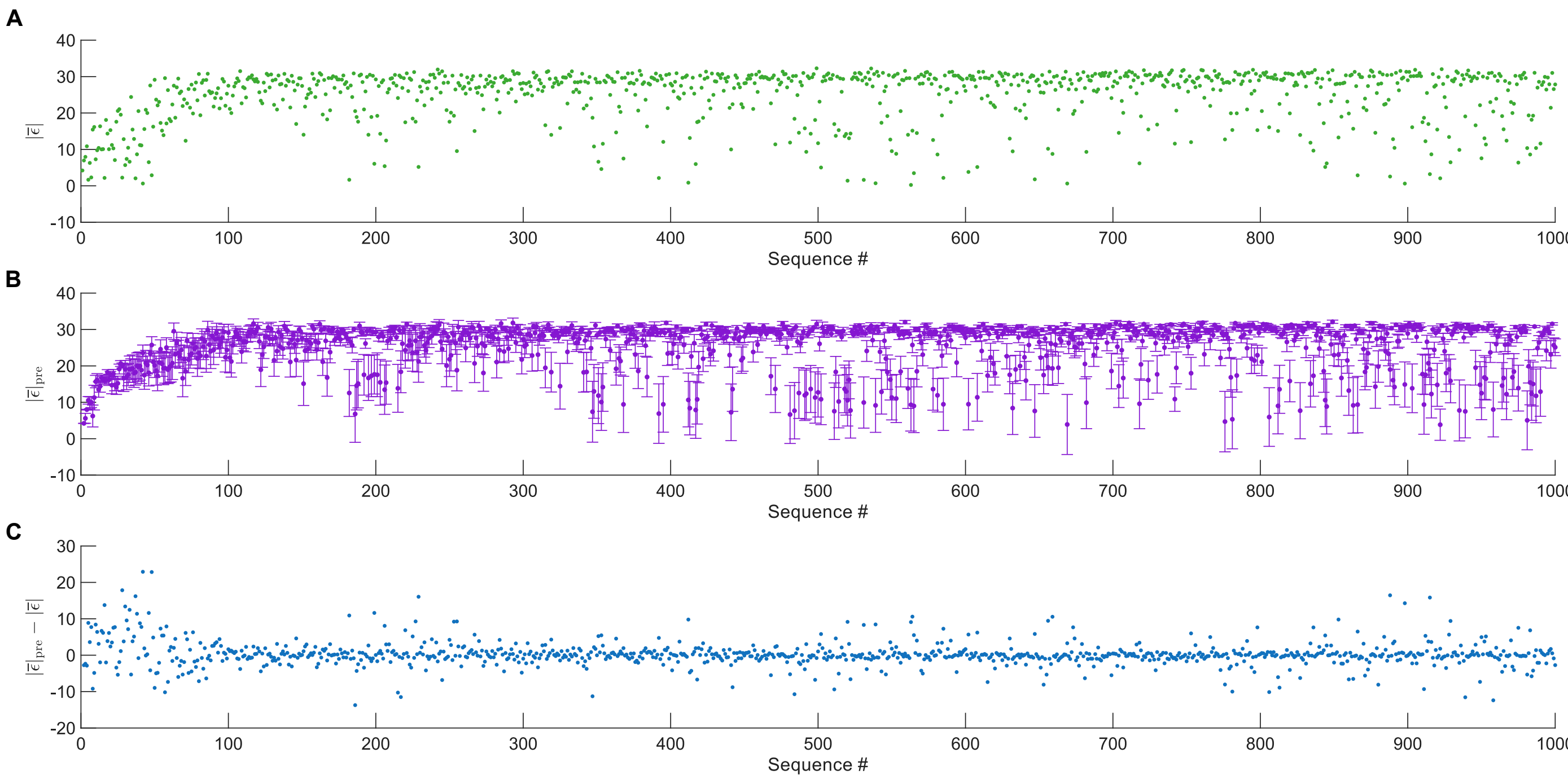

Figure S17: **Analysis of learning for Bayesian optimization of flip pulse for DNP using trityl.**

(A) Bayesian *in situ* optimization as shown in Fig. 3A. (B) Surrogate model prediction, $|\overline{\epsilon}|_{\text{pre}}$, based on previous sequence evaluations. The error bars indicate the standard deviation of the prediction. (C) Model error given as the difference between the predicted mean and the experiment feedback.

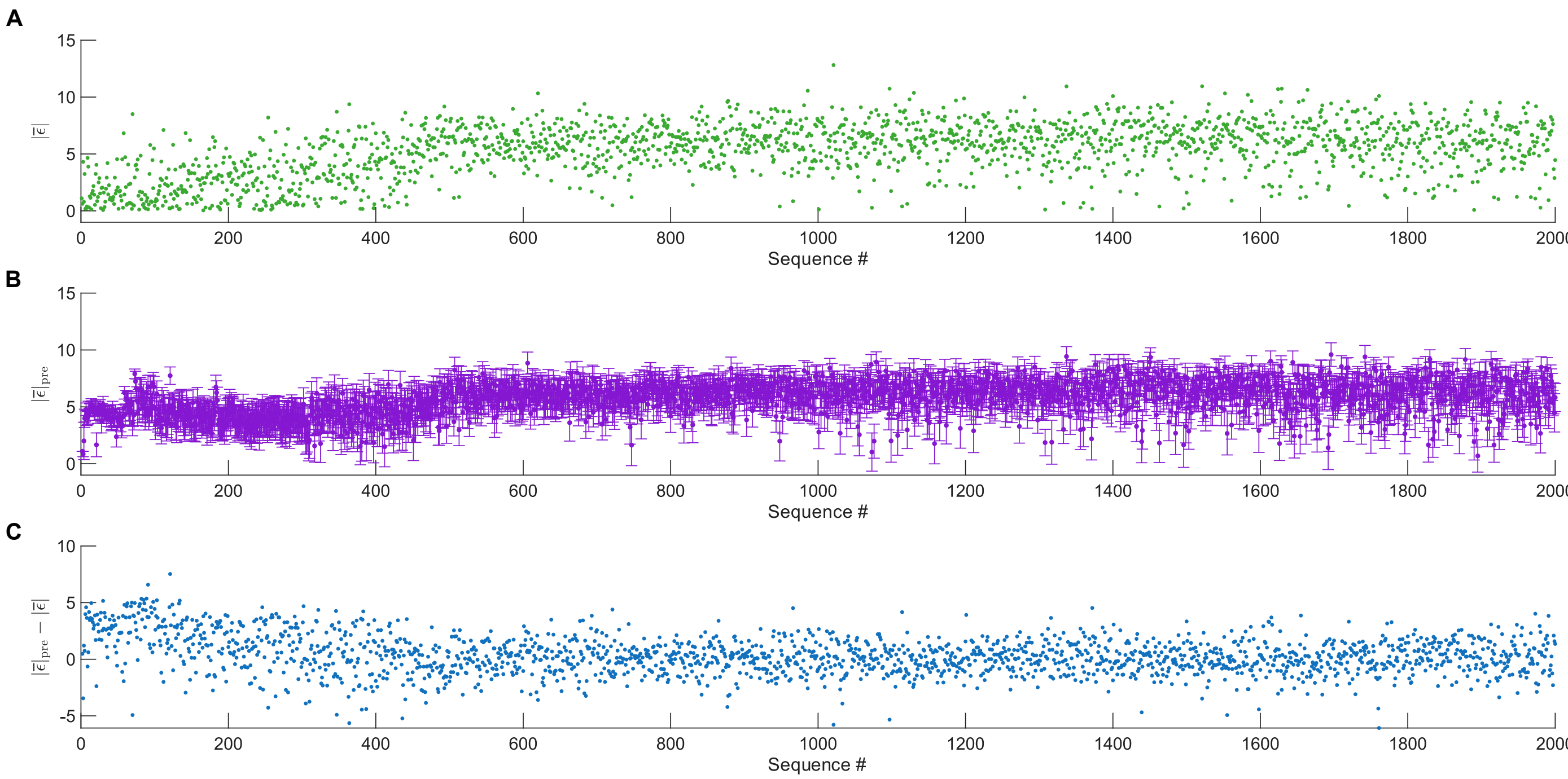

Figure S18: **Analysis of learning for Bayesian optimization of DNP using TEMPO.**

(A) Bayesian *in situ* optimization as shown in Fig. 4B. (B) Surrogate model prediction, $|\overline{\epsilon}|_{\mathrm{pre}}$, based on previous sequence evaluations. The error bars indicate the standard deviation of the prediction. (C) Model error given as the difference between the predicted mean and the experiment feedback.

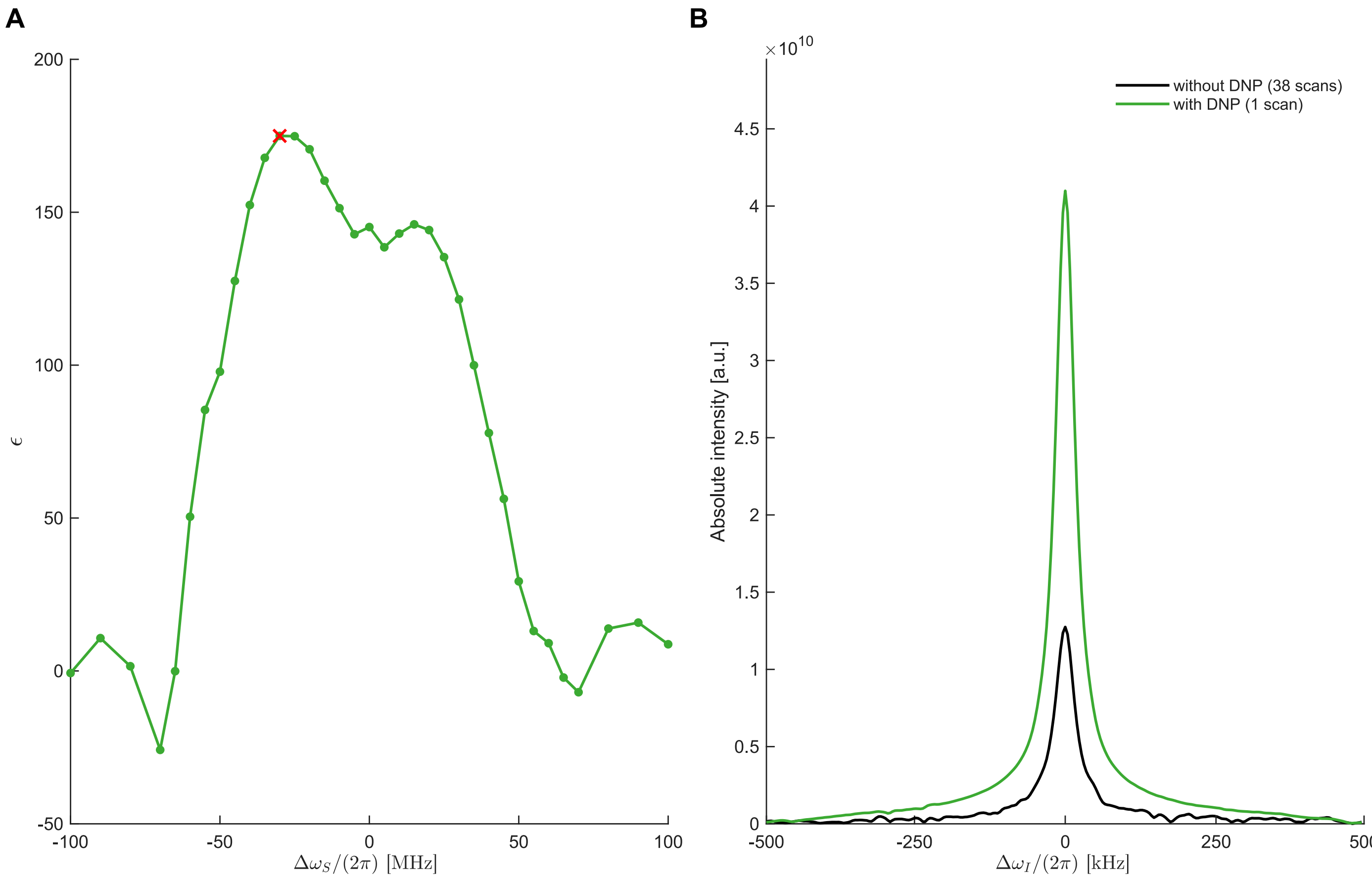

Figure S19: **Enhancement of proton signal.**

(A) Offset profile of the *in situ* Bayesian optimized 4-pulse excitation pulse followed by a cRW-OPT1 DNP transfer element (*28*) repeated 5 times as in Fig. 3C. The profiles used the same pulse sequences employed in Fig. 3C but on trityl OX063 in glycerol-d_5 and with 5 s buildup time and were compared with corresponding profiles for NOVEL, PLATO, and cRW-OPT1. (B) Comparison of ^{1}H NMR spectra with (1 scan) and without (38 scans, 128 s repetition delay) DNP for trityl OX063 in glycerol-d_8 using the DNP experiment marked by a red cross in panel (A)).

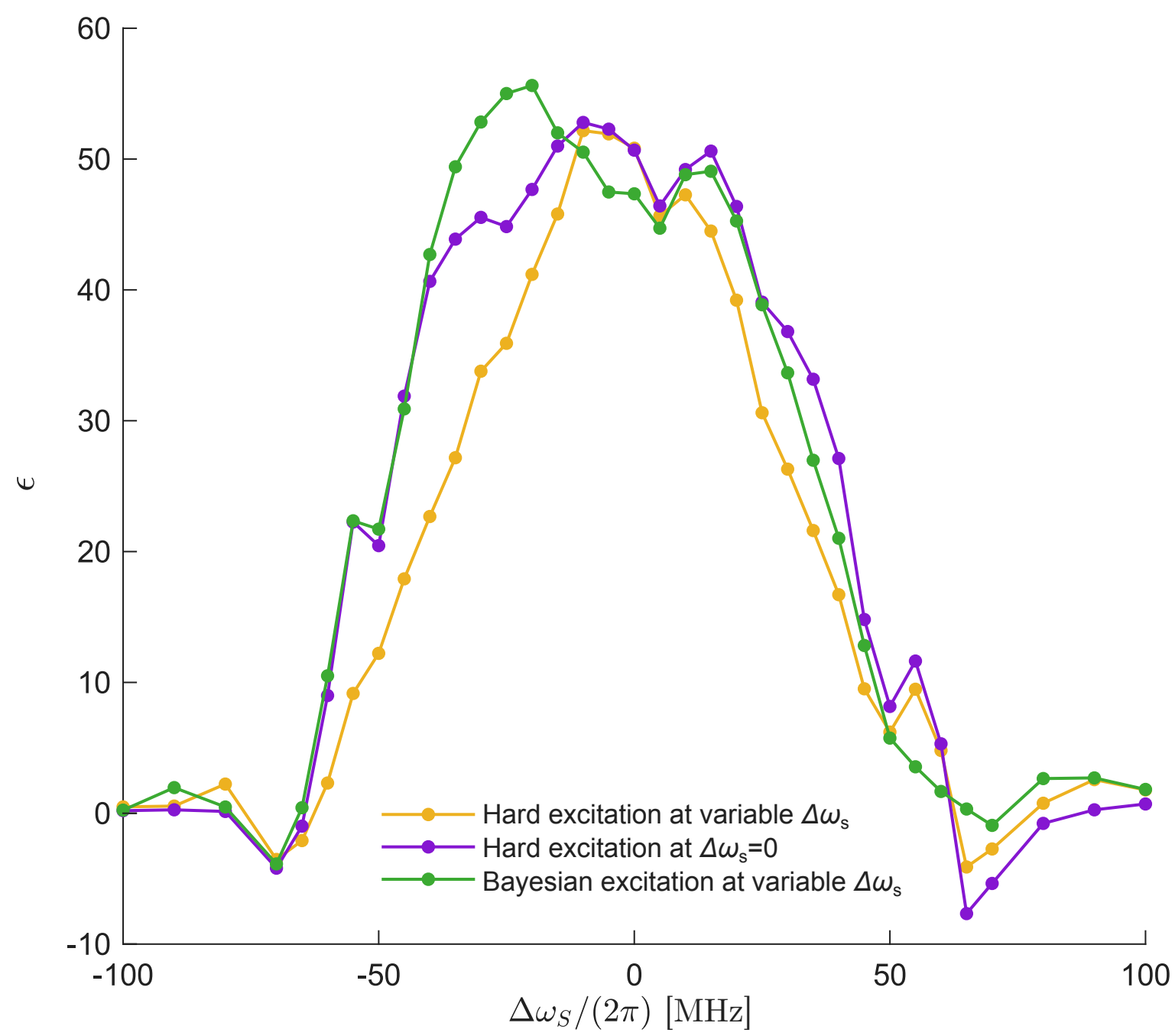

Figure S20: **Offset profiles for different flip pulses using trityl in glycerol-d5.**

Experimental *in situ* polarization enhancements as a function of electron-spin offset using the same pulse sequences employed in Fig. 2F but on a sample of trityl in glycerol-d_5 and with 2 s buildup time.

Supplementary Tables

Figure	2 Aa	2 Ba	2 Ca
Model	In-situ (Trityl)	In-situ (Trityl)	In-situ (Trityl)
acFunc	monte-carlo	expected-improvement-plus	expected-improvement-plus
deterministic	-	true	true
B0 [T]	3.4197	3.4197	3.4197
nuS [GHz]	9.74	9.74	9.74
nu1max [MHz]	49	49	49
nuI [MHz]	14.795	14.795	14.795
NMRpulseTime [μs]	1.1	1.1	1.1
eOffsets [MHz]	-60,-40,. . . ,60	-60,-40,. . . ,60	-60,-40,. . . ,60
excitePulseNum	1	1	1
exciteAmpScale	0.96	0.96	0.96
excitePhase [rad]	$\pi/2$	$\pi/2$	$\pi/2$
excitePulseTime [ns]	5	5	5
transPulseNum	6	6	6
transAmpScale	-1 to 1	-1 to 1	-1 to 1
transPhase [rad]	0	0	0
transPulseTime [ns]	20	20	20
transRepeat	1-30	1-30	1-30
RWorder	-	-	forward
constrained	false	false	true
DNPres	-	-	DQ, $k_I = 2$
DNPoffset [deg]	-	-	-5 to 5
buildup [s]	2	2	2

Table S1: Overview of settings used in Figs. 2A-C. Each setting is explained in Supplementary Text Section 1.a.

Figure	2 E
Model	In-situ (Trityl)
acFunc	expected-improvement-plus
deterministic	false
B0 [T]	3.4142
nuS [GHz]	9.72
nu1max [MHz]	42
nuI [MHz]	14.7725
NMRpulseTime [μs]	1.0
eOffsets [MHz]	-60,-40,. . . ,60
excitePulseNum	1
exciteAmpScale	1
excitePhase [rad]	$\pi/2$
excitePulseTime [ns]	5.25
transPulseNum	24
transAmpScale	-1 to 1
transPhase [rad]	0
transPulseTime [ns]	5
transRepeat	6
RWorder	factorize
constrained	true
DNPres	DQ, $k_I = 2$
DNPoffset [deg]	-16.3 to 19.3
buildup [s]	2

Table S2: Overview of settings used in Fig. 2E. Each setting is explained in Supplementary Text Section 1.a.

Figure	S6Aa	S6Ba	S6Ca
Model	In-silico (2-spin)	In-silico (2-spin)	In-silico (2-spin)
acFunc	monte-carlo	expected-improvement-plus	expected-improvement-plus
deterministic	true	true	true
nu1max [MHz]	32	32	32
nuI [MHz]	14.8	14.8	14.8
eOffsets [MHz]	-60,-59.5,. . . ,60	-60,-59.5,. . . ,60	-60,-59.5,. . . ,60
excitation	ideal	ideal	ideal
transPulseNum	6	6	6
transAmpScale	-1 to 1	-1 to 1	-1 to 1
transPhase [rad]	0	0	0
transPulseTime [ns]	20	20	20
maxContact [ns]	10e3	10e3	10e3
RWorder	-	-	forward
constrained	false	false	true
DNPres	-	-	DQ, $k_I = 2$
DNPoffset [deg]	-	-	-5 to 5
epDist [Å]	4.5	4.5	4.5
polAngles	20	20	20
MWinhom	false	false	false

Table S3: Overview of settings used in Fig. S6. Each setting is explained in Supplementary Text Section 1.a.

Figure	S7Aa	S7Ba	S7Ca
Model	In-silico (2-spin)	In-silico (2-spin)	In-silico (2-spin)
acFunc	expected-improvement-plus	expected-improvement-plus	expected-improvement-plus
deterministic	true	true	true
nu1max [MHz]	32	32	32
nuI [MHz]	14.8	14.8	14.8
eOffsets [MHz]	-60,-59.5,. . . ,60	-60,-59.5,. . . ,60	-60,-59.5,. . . ,60
excitation	ideal	ideal	ideal
transPulseNum	6	12	24
transAmpScale	-1 to 1	-1 to 1	-1 to 1
transPhase [rad]	0	0	0
transPulseTime [ns]	20	10	5
maxContact [ns]	10e3	10e3	10e3
RWorder	forward	forward	forward
constrained	true	true	true
DNPres	DQ, $k_I = 2$	DQ, $k_I = 2$	DQ, $k_I = 2$
DNPoffset [deg]	-5 to 5	-5 to 5	-5 to 5
epDist [Å]	4.5	4.5	4.5
polAngles	20	20	20
MWinhom	false	false	false

Table S4: Overview of settings used in Fig. S7. Each setting is explained in Supplementary Text Section 1.a.

Figure	S8Aa	S8Ba	S8Ca
Model	In-situ (Trityl)	In-situ (Trityl)	In-situ (Trityl)
acFunc	expected-improvement-plus	expected-improvement-plus	expected-improvement-plus
deterministic	true	true	true
B0 [T]	3.4197	3.4197	3.4197
nuS [GHz]	9.74	9.74	9.74
nu1max [MHz]	49	49	49
nuI [MHz]	14.795	14.795	14.795
NMRpulseTime [μs]	1.1	1.1	1.1
eOffsets [MHz]	-60,-40,. . . ,60	-60,-40,. . . ,60	-60,-40,. . . ,60
excitePulseNum	1	1	1
exciteAmpScale	0.96	0.96	0.96
excitePhase [rad]	$\pi/2$	$\pi/2$	$\pi/2$
excitePulseTime [ns]	5	5	5
transPulseNum	6	12	24
transAmpScale	-1 to 1	-1 to 1	-1 to 1
transPhase [rad]	0	0	0
transPulseTime [ns]	20	10	5
transRepeat	1-30	1-30	1-30
RWorder	factorize	factorize	factorize
constrained	true	true	true
DNPres	DQ, $k_I = 2$	DQ, $k_I = 2$	DQ, $k_I = 2$
DNPoffset [deg]	-5 to 5	-5 to 5	-5 to 5
buildup [s]	2	2	2

Table S5: Overview of settings used in Fig. S8. Each setting is explained in Supplementary Text Section 1.a.

Pulse	$\omega_1/(2\pi)$ [MHz]	Δt [ns]
1	10.5946	5
2	-30.2172	5
3	-17.0894	5
4	-8.83172	5
5	-41.2186	5
6	-37.1327	5
7	39.1153	5
8	25.697	5
9	-26.7359	5
10	40.6605	5
11	38.4145	5
12	26.2706	5
13	-39.0101	5
14	-41.0916	5
15	-40.6515	5
16	-41.3883	5
17	-41.9525	5
18	-41.9024	5
19	34.6693	5
20	40.1102	5
21	36.8637	5
22	7.64619	5
23	29.6807	5
24	41.5037	5

Table S6: DNP transfer sequence #1918 from Fig. 2E. The pulses are $\pm x$-phase with the sign of the amplitude defining sign of the phase. The element was repeated 6 times.

Pulse	$\omega_1/(2\pi)$ [MHz]	ϕ [Radians]	Δt [ns]
1	5.79478	5.83871	3.375
2	26.352	5.25783	3.375
3	29.4872	1.9517	3.375
4	31.8089	1.83484	3.375

Table S7: DNP excitation sequence #499 from Fig. 3A. cRW-OPT1 used $\pm x$-phase with the sign of the amplitude defining sign of the phase.

Figure	3 A
Model	In-situ (Trityl)
acFunc	expected-improvement-plus
deterministic	false
B0 [T]	3.4125
nuS [GHz]	9.72
nu1max [MHz]	45
nuI [MHz]	14.765
NMRpulseTime [μs]	0.95
eOffsets [MHz]	-60,-40,. . . ,60
excitePulseNum	4
exciteAmpScale	0 to 1
excitePhase [rad]	0 to 2π
excitePulseTime [ns]	1.25-5
transPulseNum	30
transAmpScale	cRW-OPT1
transPhase [rad]	0
transPulseTime [ns]	5
transRepeat	5
RWorder	forward
constrained	false
DNPres	DQ, $k_I = 2$
DNPoffset [deg]	0.8
buildup [s]	5

Table S8: Overview of settings used in Fig. 3A. Each setting is explained in Supplementary Text Section 1.a.

Figure	S11A	S11B
Model	In-situ (TEMPO)	In-situ (TEMPO)
acFunc	expected-improvement-plus	expected-improvement-plus
deterministic	false	false
B0 [T]	3.408	3.408
nuS [GHz]	9.72	9.72
nu1max [MHz]	52	52
nuI [MHz]	14.745	14.745
NMRpulseTime [μs]	1.1	1.1
eOffsets [MHz]	0	0
excitePulseNum	4	0
exciteAmpScale	0 to 1	-
excitePhase [rad]	0 to 2π	-
excitePulseTime [ns]	0 to 5	-
transPulseNum	4	20
transAmpScale	0 to 1	0 to 1
transPhase [rad]	0 to 2π	0 to 2π
transContact [ns]	10e3	10e3
transRepeat	1 to 50	1 to 100
constrained	false	false
buildup [s]	8	8

Table S9: Overview of settings used in Figs. S11A-B. Each setting is explained in Supplementary Text Section 1.a

Figure	S11C	S11D
Model	In-situ (TEMPO)	In-situ (TEMPO)
acFunc	expected-improvement-plus	expected-improvement-plus
deterministic	false	false
B0 [T]	3.408	3.408
nuS [GHz]	9.72	9.72
nu1max [MHz]	52	52
nuI [MHz]	14.745	14.745
NMRpulseTime [μs]	1.1	1.1
eOffsets [MHz]	0	0
excitePulseNum	1	4
exciteAmpScale	1	0 to 1
excitePhase [rad]	$\pi/2$	0 to 2π
excitePulseTime [ns]	5.5	0 to 5
transPulseNum	30	10
transAmpScale	-1 to 1	0 to 1
transPhase [rad]	0	0 to 2π
transContact [ns]	10e3	0 to 10e3
transRepeat	1 to 100	1 to 100
constrained	false	false
buildup [s]	8	8

Table S10: Overview of settings used in Figs. S11C-D. Each setting is explained in Supplementary Text Section 1.a.

Pulse	$\omega_1/(2\pi)$ [MHz]	ϕ [Radians]	Δt [ns]
1	42.0768	0	4
2	46.6575	4.43443	4
3	47.5536	4.02352	4
4	49.6457	3.08115	4
5	25.9887	2.52414	1250
6	23.6053	2.11097	1250
7	9.33832	2.9136	1250
8	27.4136	3.87421	1250
9	21.8394	2.41519	1250
10	23.3238	2.08272	1250
11	42.2376	1.97648	1250
12	22.9295	2.7669	1250

Table S11: DNP sequence #1021 from Fig. 4B. Transfer sequence was not repeated.

Figure	4 A	4 B
Model	In-situ (TEMPO)	In-situ (TEMPO)
acFunc	monte-carlo	expected-improvement-plus
deterministic	-	false
B0 [T]	3.408	3.408
nuS [GHz]	9.72	9.72
nu1max [MHz]	52	52
nuI [MHz]	14.745	14.745
NMRpulseTime [μs]	1.1	1.1
eOffsets [MHz]	0	0
excitePulseNum	4	4
exciteAmpScale	0 to 1	0 to 1
excitePhase [rad]	0 to 2π	0 to 2π
excitePulseTime [ns]	0 to 5	0 to 5
transPulseNum	8	8
transAmpScale	0 to 1	0 to 1
transPhase [rad]	0 to 2π	0 to 2π
transContact [ns]	10e3	10e3
transRepeat	1	1
constrained	false	false
buildup [s]	8	8

Table S12: Overview of settings used in Fig. 4. Each setting is explained in Supplementary Text Section 1.a.

Figure	S16Aa	S16Ba	S16Ca
Model	In-silico (2-spin)	In-silico (2-spin)	In-silico (2-spin)
acFunc	expected-improvement	expected-improvement	expected-improvement
deterministic	true	true	true
nu1max [MHz]	32	32	32
nuI [MHz]	14.8	14.8	14.8
eOffsets [MHz]	-60,-59.5,. . . ,60	-60,-59.5,. . . ,60	-60,-59.5,. . . ,60
excitation	ideal	ideal	ideal
transPulseNum	12	12	12
transAmpScale	-1 to 1	-1 to 1	-1 to 1
transPhase [rad]	0	0	0
transPulseTime [ns]	10	10	10
maxContact [ns]	10e3	10e3	10e3
RWorder	forward	backtrack	factorize
constrained	true	true	true
DNPres	DQ, $k_I = 2$	DQ, $k_I = 2$	DQ, $k_I = 2$
DNPoffset [deg]	-5 to 5	-5 to 5	-5 to 5
epDist [Å]	4.5	4.5	4.5
polAngles	20	20	20
MWinhom	false	false	false

Table S13: Overview of settings used in the first column of Fig. S16. Each setting is explained in Supplementary Text Section 1.a.

Figure	S16Ab	S16Bb	S16Cb
Model	In-silico (2-spin)	In-silico (2-spin)	In-silico (2-spin)
acFunc	expected-improvement-plus	expected-improvement-plus	expected-improvement-plus
deterministic	true	true	true
nu1max [MHz]	32	32	32
nuI [MHz]	14.8	14.8	14.8
eOffsets [MHz]	-60,-59.5,. . . ,60	-60,-59.5,. . . ,60	-60,-59.5,. . . ,60
excitation	ideal	ideal	ideal
transPulseNum	12	12	12
transAmpScale	-1 to 1	-1 to 1	-1 to 1
transPhase [rad]	0	0	0
transPulseTime [ns]	10	10	10
maxContact [ns]	10e3	10e3	10e3
RWorder	forward	backtrack	factorize
constrained	true	true	true
DNPres	DQ, $k_I = 2$	DQ, $k_I = 2$	DQ, $k_I = 2$
DNPoffset [deg]	-5 to 5	-5 to 5	-5 to 5
epDist [Å]	4.5	4.5	4.5
polAngles	20	20	20
MWinhom	false	false	false

Table S14: Overview of settings used in the second column of Fig. S16. Each setting is explained in Supplementary Text Section 1.a.

Figure	S16Ac	S16Bc	S16Cc
Model	In-silico (2-spin)	In-silico (2-spin)	In-silico (2-spin)
acFunc	lower-confidence-bound	lower-confidence-bound	lower-confidence-bound
deterministic	true	true	true
nu1max [MHz]	32	32	32
nuI [MHz]	14.8	14.8	14.8
eOffsets [MHz]	-60,-59.5,. . . ,60	-60,-59.5,. . . ,60	-60,-59.5,. . . ,60
excitation	ideal	ideal	ideal
transPulseNum	12	12	12
transAmpScale	-1 to 1	-1 to 1	-1 to 1
transPhase [rad]	0	0	0
transPulseTime [ns]	10	10	10
maxContact [ns]	10e3	10e3	10e3
RWorder	forward	backtrack	factorize
constrained	true	true	true
DNPres	DQ, $k_I = 2$	DQ, $k_I = 2$	DQ, $k_I = 2$
DNPoffset [deg]	-5 to 5	-5 to 5	-5 to 5
epDist [Å]	4.5	4.5	4.5
polAngles	20	20	20
MWinhom	false	false	false

Table S15: Overview of settings used in the third column of Fig. S16. Each setting is explained in Supplementary Text Section 1.a.

Figure	S16Ad	S16Bd	S16Cd
Model	In-silico (2-spin)	In-silico (2-spin)	In-silico (2-spin)
acFunc	probability-of-improvement	probability-of-improvement	probability-of-improvement
deterministic	true	true	true
nu1max [MHz]	32	32	32
nuI [MHz]	14.8	14.8	14.8
eOffsets [MHz]	-60,-59.5,. . . ,60	-60,-59.5,. . . ,60	-60,-59.5,. . . ,60
excitation	ideal	ideal	ideal
transPulseNum	12	12	12
transAmpScale	-1 to 1	-1 to 1	-1 to 1
transPhase [rad]	0	0	0
transPulseTime [ns]	10	10	10
maxContact [ns]	10e3	10e3	10e3
RWorder	forward	backtrack	factorize
constrained	true	true	true
DNPres	DQ, $k_I = 2$	DQ, $k_I = 2$	DQ, $k_I = 2$
DNPoffset [deg]	-5 to 5	-5 to 5	-5 to 5
epDist [Å]	4.5	4.5	4.5
polAngles	20	20	20
MWinhom	false	false	false

Table S16: Overview of settings used in the fourth column of Fig. S16. Each setting is explained in Supplementary Text Section 1.a.

Amplitude factor	Weight [%]
0.65	4.84
0.70	5.29
0.75	5.86
0.80	6.62
0.85	7.69
0.90	9.39
0.95	12.73
1.00	32.88
1.05	14.68

Table S17: The MW inhomogeneity profile used in Figs. 3E and 3F. For each row, the simulation using inhomogeneity averaging was run with the MW amplitudes multiplied by the amplitude factor. (*57*) The weighted average was then taken using the specified weight from each row.

REFERENCES

1. F. Bloch, Nuclear induction. *Phys. Rev.* **70**, 460–474 (1946).

2. E. L. Hahn, Spin echoes. *Phys. Rev.* **80**, 580–594 (1950).

3. N. F. Ramsey, A molecular beam resonance method with separated oscillating fields. *Phys. Rev.* **78**, 695–699 (1950).

4. N. A. Kurnit, I. D. Abella, S. R. Hartmann, Observation of a photon echo. *Phys. Rev. Lett.* **13**, 567–568 (1964).

5. J. H. Freed, Theory of saturation and double-resonance effects in ESR spectra. *J. Chem. Phys.* **43**, 2312–2332 (1965).

6. A. Abragam, *The Principles of Nuclear Magnetism*, International Series of Monographs on Physics (Clarendon Press, 1961).

7. R. R. Ernst, G. Bodenhausen, A. Wokaun, *Principles of Nuclear Magnetic Resonance in One and Two Dimensions* (Oxford Univ. Press, 1990).

8. A. Schweiger, G. Jeschke, *Principles of Pulse Electron Paramagnetic Resonance* (Oxford Univ. Press, 2001).

9. U. Haeberlen, J. S. Waugh, Coherent averaging effects in magnetic resonance. *Phys. Rev.* **175**, 453–467 (1968).

10. M. Hohwy, N. C. Nielsen, Systematic design and evaluation of multiple-pulse experiments in nuclear magnetic resonance spectroscopy using a semi-continuous Baker–Campbell–Hausdorff expansion. *J. Chem. Phys.* **109**, 3780–3791 (1998).

11. T. S. Untidt, N. C. Nielsen, Closed solution to the Baker-Campbell-Hausdorff problem: Exact effective Hamiltonian theory for analysis of nuclear-magnetic-resonance experiments. *Phys. Rev. E* **65**, 021108 (2002).

12. D. Siminovitch, T. Untidt, N. C. Nielsen, Exact effective Hamiltonian theory. II. Polynomial expansion of matrix functions and entangled unitary exponential operators. *J. Chem. Phys.* **120**, 51–66 (2004).

13. R. Shankar, M. Ernst, P. K. Madhu, T. Vosegaard, N. C. Nielsen, A. B. Nielsen, A general theoretical description of the influence of isotropic chemical shift in dipolar recoupling experiments for solid-state NMR. *J. Chem. Phys.* **146**, 134105 (2017).

14. A. B. Nielsen, M. R. Hansen, J. E. Andersen, T. Vosegaard, Single-spin vector analysis of strongly coupled nuclei in TOCSY NMR experiments. *J. Chem. Phys.* **151**, 134117 (2019).

15. A. B. Nielsen, N. C. Nielsen, Accurate analysis and perspectives for systematic design of magnetic resonance experiments using single-spin vector and exact effective Hamiltonian theory. *J. Magn. Reson. Open* **12-13**, 100064 (2022).

16. N. Khaneja, T. Reiss, C. Kehlet, T. Schulte-Herbrüggen, S. J. Glaser, Optimal control of coupled spin dynamics: Design of NMR pulse sequences by gradient ascent algorithms. *J. Magn. Reson.* **172**, 296–305 (2005).

17. I. I. Maximov, Z. Tošner, N. C. Nielsen, Optimal control design of NMR and dynamic nuclear polarization experiments using monotonically convergent algorithms. *J. Chem. Phys.* **128**, 184505 (2008).

18. Z. Tošner, T. Vosegaard, C. Kehlet, N. Khaneja, S. J. Glaser, N. C. Nielsen, Optimal control in NMR spectroscopy: Numerical implementation in SIMPSON. *J. Magn. Reson.* **197**, 120–134 (2009).

19. I. I. Maximov, J. Salomon, G. Turinici, N. C. Nielsen, A smoothing monotonic convergent optimal control algorithm for nuclear magnetic resonance pulse sequence design. *J. Chem. Phys.* **132**, 084107 (2010).

20. D. L. Goodwin, I. Kuprov, Modified Newton-Raphson GRAPE methods for optimal control of spin systems. *J. Chem. Phys.* **144**, 204107 (2016).

21. J. P. Carvalho, D. L. Goodwin, N. Wili, A. B. Nielsen, N. C. Nielsen, Optimal control design strategies for pulsed dynamic nuclear polarization. *J. Chem. Phys.* **162**, 054111 (2025).

22. D. L. Goodwin, W. K. Myers, C. R. Timmel, I. Kuprov, Feedback control optimisation of ESR experiments. *J. Magn. Reson.* **297**, 9–16 (2018).

23. G. De Paëpe, P. Hodgkinson, L. Emsley, Improved heteronuclear decoupling schemes for solid-state magic angle spinning NMR by direct spectral optimization. *Chem. Phys. Lett.* **376**, 259–267 (2003).

24. A. Marshall, T. Reisser, P. Rembold, C. Müller, J. Scheuer, M. Gierse, T. Eichhorn, J. M. Steiner, P. Hautle, T. Calarco, F. Jelezko, M. B. Plenio, S. Montangero, I. Schwartz, M. M. Müller, P. Neumann, Macroscopic hyperpolarization enhanced with quantum optimal control. *Phys. Rev. Res.* **4**, 043179 (2022).

25. A. Doll, G. Jeschke, Wideband frequency-swept excitation in pulsed EPR spectroscopy. *J. Magn. Reson.* **280**, 46–62 (2017).

26. N. Wili, A. B. Nielsen, L. A. Völker, L. Schreder, N. C. Nielsen, G. Jeschke, K. O. Tan, Designing broadband pulsed dynamic nuclear polarization sequences in static solids. *Sci. Adv.* **8**, eabq0536 (2022).

27. A. B. Nielsen, J. P. A. Carvalho, D. L. Goodwin, N. Wili, N. C. Nielsen, Dynamic nuclear polarization pulse sequence engineering using single-spin vector effective Hamiltonians. *Phys. Chem. Chem. Phys.* **26**, 28208–28219 (2024).

28. A. B. Nielsen, J. P. Carvalho, N. Wili, F. V. Jensen, D. L. Goodwin, T. S. Untidt, Z. Tošner, N. C. Nielsen, Controlling effective Hamiltonians: Broadband pulsed dynamic nuclear polarization by constrained random walk and non-linear optimization. *J. Chem. Phys.* **163**, 144111 (2025).

29. J. Carvalho, A. B. Nielsen, D. L. Goodwin, N. Wili, N. C. Nielsen, Longitudinal pulsed dynamic nuclear polarization transfer via periodic optimal control. *J. Phys. Chem. Lett.* **17**, 2517–2523 (2026).

30. J. Stropp, N. Wili, N. C. Nielsen, D. Klose, Increased sensitivity in electron–nuclear double resonance spectroscopy with chirped radiofrequency pulses. *Magn. Reson.* **6**, 33–42 (2025).

31. N. Wili, A. B. Nielsen, J. P. Carvalho, N. C. Nielsen, Observation of dynamic nuclear polarization echoes. *Sci. Adv.* **10**, eadr2420 (2024).

32. MATLAB, *version 24.2.0 (R2024b)* (The MathWorks Inc., 2024).

33. J. A. Nelder, R. Mead, A simplex method for function minimization. *Comput. J.* **7**, 308–313 (1965).

34. W. Press, S. Teukolsky, W. Vetterling, B. Flannery, *Numerical Recipes: The Art of Scientific Computing* (Cambridge Univ. Press, ed. 3, 2007).

35. S. Kirkpatrick, C. D. Gelatt, M. P. Vecchi, Optimization by simulated annealing. *Science* **220**, 671–680 (1983).

36. Y. Jin, *Evolutionary Algorithms* (Physica-Verlag HD, 2003), pp. 49–71.

37. D. C. Sorensen, Newton's method with a model trust region modification. *SIAM J. Numer. Anal.* **19**, 409–426 (1982).

38. S. M. Goldfeld, R. E. Quandt, H. F. Trotter, Maximization by quadratic hill-climbing. *Econometrica* **34**, 541–551 (1966).

39. J. H. Ardenkjaer-Larsen, On the present and future of dissolution-DNP. *J. Magn. Reson.* **264**, 3–12 (2016).

40. G. Mathies, S. Jain, M. Reese, R. G. Griffin, Pulsed dynamic nuclear polarization with trityl radicals. *J. Phys. Chem. Lett.* **7**, 111–116 (2016).

41. A. Henstra, P. Dirksen, J. Schmidt, W. Wenckebach, Nuclear spin orientation via electron spin locking (NOVEL). *J. Magn. Reson.* **77**, 389–393 (1988).

42. S. K. Jain, G. Mathies, R. G. Griffin, Off-resonance NOVEL. *J. Chem. Phys.* **147**, 164201 (2017).

43. K. O. Tan, C. Yang, R. T. Weber, G. Mathies, R. G. Griffin, Time-optimized pulsed dynamic nuclear polarization. *Sci. Adv.* **5**, eaav6909 (2019).

44. V. S. Redrouthu, G. Mathies, Efficient pulsed dynamic nuclear polarization with the X-inverse-X sequence. *J. Am. Chem. Soc.* **144**, 1513–1516 (2022).

45. V. S. Redrouthu, S. Vinod-Kumar, G. Mathies, Dynamic nuclear polarization by two-pulse phase modulation. *J. Chem. Phys.* **159**, 014201 (2023).

46. S. A. Jegadeesan, Y. Zhao, G. M. Smith, I. Kuprov, G. Mathies, Simulation of pulsed dynamic nuclear polarization in the steady state. *J. Chem. Phys.* **163**, 034111 (2025).

47. I. Schwartz, J. Scheuer, B. Tratzmiller, S. Müller, Q. Chen, I. Dhand, Z.-Y. Wang, C. Müller, B. Naydenov, F. Jelezko, M. B. Plenio, Robust optical polarization of nuclear spin baths using Hamiltonian engineering of nitrogen-vacancy center quantum dynamics. *Sci. Adv.* **4**, eaat8978 (2018).

48. P. I. Frazier, A tutorial on Bayesian optimization. arXiv:1807.02811 [stat.ML] (2018).

49. S. Greenhill, S. Rana, S. Gupta, P. Vellanki, S. Venkatesh, Bayesian optimization for adaptive experimental design: A review. *IEEE Access* **8**, 13937–13948 (2020).

50. C. E. Rasmussen, C. K. I. Williams, *Gaussian Processes for Machine Learning* (The MIT Press, 2005).

51. J. Snoek, H. Larochelle, R. P. Adams, "Practical Bayesian optimization of machine learning algorithms" in *Proceedings of the 26th International Conference on Neural Information Processing Systems—Volume 2, NIPS'12*. (Curran Associates Inc., 2012), pp. 2951–2959.

52. B. Letham, B. Karrer, G. Ottoni, E. Bakshy, Constrained Bayesian optimization with noisy experiments. *Bayesian Anal.* **14**, 495–519 (2019).

53. H. B. Moss, D. S. Leslie, J. Gonzalez, P. Rayson, GIBBON: General-purpose information-based Bayesian optimisation. *J. Mach. Learn. Res.* **22**, 1–49 (2021).

54. M. Binois, N. Wycoff, A survey on high-dimensional gaussian process modeling with application to bayesian optimization. *ACM Trans. Evol. Learn. Optim.* **2**, 1–26 (2022).

55. J. P. Carvalho, A. B. Nielsen, E. Baligács, N. Wili, N. C. Nielsen, Bridging dynamic nuclear polarization and solid-state NMR dipolar recoupling: From static single crystal to spinning powders. *J. Phys. Chem. Lett.* **16**, 4363–4371 (2025).

56. D. L. Goodwin, J. P. Carvalho, A. B. Nielsen, N. Wili, A. Brinkmann, T. Vosegaard, Z. Tošner, N. C. Nielsen, Extending numerical simulations in SIMPSON: Electron paramagnetic resonance, dynamic nuclear polarisation, propagator splitting, pulse transients, and quadrupolar cross terms. *J. Magn. Reson. Open* **27**, 100218 (2026).

57. R. Gupta, G. Hou, T. Polenova, A. J. Vega, RF inhomogeneity and how it controls CPMAS. *Solid State Nucl. Magn. Reson.* **72**, 17–26 (2015).

58. Matlab documentation on Bayesian Optimization Algorithm; https://se.mathworks.com/help/stats/bayesian-optimization-algorithm.html#bvaz8tr-1.

59. A. D. Bull, Convergence rates of efficient global optimization algorithms. *J. Mach. Learn. Res.* **12**, 2879–2904 (2011).

60. M. A. Gelbart, J. Snoek, R. P. Adams, Bayesian optimization with unknown constraints. arXiv:1403.5607 [stat.ML] (2014).
